# Complex wireframe DNA nanostructures from simple building blocks

**DOI:** 10.1038/s41467-019-08647-7

**Published:** 2019-03-06

**Authors:** Wen Wang, Silian Chen, Byoungkwon An, Kai Huang, Tanxi Bai, Mengyuan Xu, Gaëtan Bellot, Yonggang Ke, Ye Xiang, Bryan Wei

**Affiliations:** 1 School of Life Sciences, Tsinghua University-Peking University Center for Life Sciences, Center for Synthetic and Systems Biology, Tsinghua University, 100084 Beijing, China; 2 Center for Infectious Disease Research, Collaborative Innovation Center for Diagnosis and Treatment of Infectious Diseases, Beijing Advanced Innovation Center for Structural Biology, Department of Basic Medical Sciences, School of Medicine, Tsinghua University, 100084 Beijing, China; 3 School of Life Sciences, Peking University, 100084 Beijing, China; 4 BioNano Research Group, Autodesk Life Sciences, Pier 9, San Francisco, CA 94111 USA; 5 Centre de Biochimie Structurale, Montpellier, France; 6 Wallace H. Coulter Department of Biomedical Engineering, Georgia Institute of Technology and Emory University, 30322 Atlanta, GA USA

## Abstract

DNA nanostructures with increasing complexity have showcased the power of programmable self-assembly from DNA strands. At the nascent stage of the field, a variety of small branched objects consisting of a few DNA strands were created. Since then, a quantum leap of complexity has been achieved by a scaffolded ‘origami’ approach and a scaffold-free approach using single-stranded tiles/bricks—creating fully addressable two-dimensional and three-dimensional DNA nanostructures designed on densely packed lattices. Recently, wireframe architectures have been applied to the DNA origami method to construct complex structures. Here, revisiting the original wireframe framework entirely made of short synthetic strands, we demonstrate a design paradigm that circumvents the sophisticated routing and size limitations intrinsic to the scaffold strand in DNA origami. Under this highly versatile self-assembly framework, we produce a myriad of wireframe structures, including 2D arrays, tubes, polyhedra, and multi-layer 3D arrays.

## Abstract (web summary)

DNA nanostructures of increasing complexity have been designed using scaffolded origami or single-stranded tiles and bricks. Here the authors demonstrate the construction of complex structures using a wireframe approach that overcomes limitations of using scaffolding.

## Introduction

**Fig. 1:**
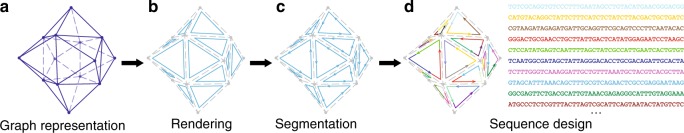
Design pipeline for a typical wireframe structure composed of synthetic strands. **a** A specific two-dimensional (2D) or three-dimensional (3D) graph representation of a targeted structure. **b** A node-edge network rendering. Nodes represent vertices of different numbers of arms and the edges represent duplexes of variable lengths. **c** Segmentation of edges into complementary domains. **d** Sequence generation of distinct component strands

**Fig. 2:**
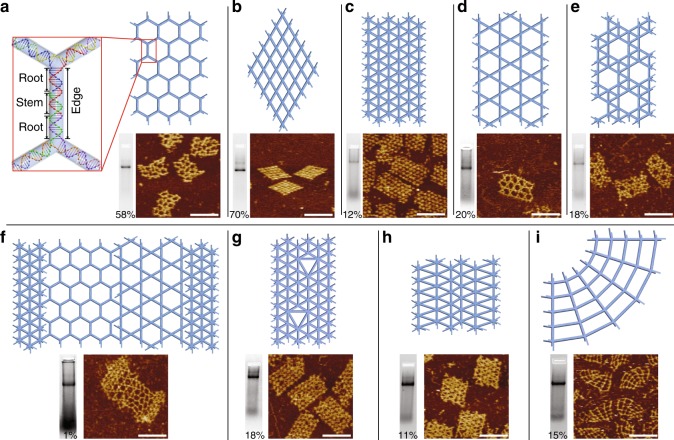
Two-dimensional (2D) wireframe arrays of different tessellation patterns. **a** Hexagonal tessellation pattern with 3-arm verties. Inset shows a strand diagram of a typical edge (a stem domain sandwiched by two root domains). **b** Square tessellation pattern with 4-arm vertices. **c** Triangular tessellation pattern with 6-arm vertices. **d** Trihexagonal tessellation pattern with 4-arm vertices. **e** Snub trihexagonal tessellation pattern with 5-arm vertices. **f** Chimeric pattern with individual blocks composed of 3-arm, 4-arm, and 6-arm vertices. **g**, **h** Two irregular triangular patterns with 6-arm vertices. **i** Cobweb-like pattern with 4-arm vertices. Top: schematic diagrams of the wireframe structures; bottom: native agarose gel electrophoresis results (bottom left, numbers indicate assembly yields) and AFM images (bottom right, scale bars: 100 nm)

**Fig. 3:**
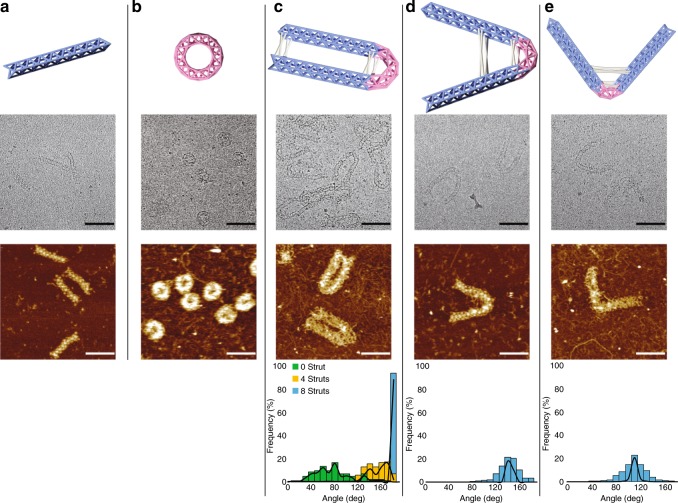
Wireframe tubes. Schematic diagrams of straight tube (**a**), donut (**b**), U-bent (180°-bent) (**c**), 135°-bent (**d**), and 90°-bent (**e**) tubes on the top row; cryo-EM images (scale bars: 100 nm) on the second row; AFM images (scale bars: 100 nm) on the third row; histograms of bending angles (solid curves represent normal distribution) on the bottom row of **c**–**e**

The field of DNA nanotechnology has enjoyed extraordinary success in constructing structures with ever increasing complexity, as defined by the number of addressable components^1–4^. Two methods have stood out: the scaffolded origami approach of folding a long scaffold with help of many short staple strands^5–16^ and the scaffold-free approach with locally connected DNA LEGO™ bricks to determine global shape^17–21^. Phenomenal progress has been made in several recent studies to upgrade both size and complexity of the DNA structures, including two-dimensional (2D) and three-dimensional (3D) arrays with an unprecedented number of addressable components^15,16,21^. Recently, wireframe architecture has also been introduced to the origami approach to make structures using sophisticated scaffold routing^12–14^. On the other hand, despite scaffold-free assembly’s capacity to employ up to 33,000 distinct strands^21^ as compared to DNA origami’s 1600 distinct strands^22^, complex wireframe architectures have not been fully demonstrated with the scaffold-free approach^23–33^. One of the challenges was believed to be limited structural stability in the absence of a long scaffold^34^. However, the advantages of using a scaffold-free approach to assemble complex wireframe structures entirely out of short DNA strands are clear: the design process would be streamlined without having to route a long scaffold, and structure size would no longer be constrained by scaffold length. Therefore, the scaffold-free wireframe framework potentially provides more design freedom and is a more generalizable design platform for arbitrary structures. A typical process in designing a wireframe structure from short synthetic strands is straightforward^35^, and it is pipelined as: (1) rendering a specific 2D or 3D geometry (Fig. 1a) as a node-edge network with DNA junctions of different numbers of arms as nodes and DNA duplexes of variable lengths as edges (Fig. 1b), (2) segmenting edges to complementary domains to ensure that corresponding strands with multiple domains satisfy synthesis and self-assembly requirements (Fig. 1c), and (3) populating strands with sequences to meet certain sequence generation criteria^36^ (Fig. 1d). Facilitated by our design program, we have designed, constructed, and characterized many wireframe structures. This includes 2D arrays of regular, quasi-regular or irregular tessellation patterns with vertices of different numbers of arms, tubes of different shapes, multiple polyhedra with more sophisticated tessellation connectivity, and most importantly, multi-layer 3D arrays with 4-arm or 6-arm junctions acting as vertices. The robust self-assembly proves that the local interactions among short strands can collectively provide sufficient overall structural integrity and while precisely defining a complex geometry. A reincarnation of the original blueprint of DNA nanotechnology, our findings in this study redefine the feasibility of using flexible components to self-assemble DNA nanostructures in an adaptive fashion.

## Results

### Structural design

To design a typical wireframe structure entirely from synthetic strands, a 2D or 3D graph is first generated (Fig. 1a). Such a graph, composed of nodes and edges with a specific node-edge geometry, is rendered so that the nodes represent vertices of different numbers of arms and the edges represent simple DNA duplexes of variable lengths (Fig. 1b). The number of edges connected to a certain node is arbitrary, as are the numbers of base pairs of constituent edges. Arbitrary shapes can be designed by defining a full set of node-node connectivity and the corresponding edges of variable lengths. For purposes of practicality, the number of edges connecting to a certain vertex is set to be no more than seven and the edge lengths are set to be full turns (rounded multiples of 10.5 base pairs) in this study. Afterwards, edges are segmented into complementary domains to ensure that the corresponding strands with multiple domains satisfy synthesis (e.g., a typical strand is less than 100 nt and preferably less than 80 nt) and self-assembly (e.g., a paired complementary domain is no less than 6 bp and preferably no less than 10 bp) requirements (Fig. 1c). Although the segmentation could also be arbitrary, a typical edge in this study is segmented in a standardized fashion into two root domains of the same length and a stem domain (Fig. 2a, inset and Supplementary Fig. 1). To relax tension around the crossovers at a certain vertex (e.g., a vertex of 5 or 6 arms), extra unpaired base(s) (e.g., 1T, 2T, or 3T) can be added as spacers across arms (Supplementary Table 1). After a specific strand arrangement is laid out, the final step is to populate strands with sequences to meet certain sequence generation criteria^36^ (e.g., base-pairing and mismatch prevention). As colors indicate, a distinct DNA sequence was appointed to each component strand (Fig. 1d).

### Two-dimensional (2D) arrays

We first designed five addressable 2D wireframe structures of different patterns: three regular tessellation patterns containing 3-arm, 4-arm, and 6-arm vertices, respectively (Fig. 2a–c); and two quasi-regular tessellation patterns containing 4-arm and 5-arm vertices, respectively (Fig. 2d, e). Besides the typical arrangement of 11-bp root domains and 10-bp stem domains (inset of Fig. 2a), other segmentation arrangements (e.g., 13-bp root domains with 6-bp stem domains, and 16-bp root domains with 10-bp stem domains) also led to successful assembly of 2D wireframe structures (Supplementary Fig. 12 and Supplementary Table 3). T2 linkers were added to crossover points at 5-arm or 6-arm vertices to mitigate the stronger electrostatic repulsion caused by the proximity of neighboring arms. Such design strategy was also implemented in the structures shown later in this article (Supplementary Table 1) and similar implementation can also be found in earlier studies of DNA wireframe structures^12,26,32^.

The structures of regular or quasi-regular patterns described above have uniform edge length (e.g., 32 bp). We next designed four structures with variable connectivity patterns and edge lengths. In the first example, we constructed a chimeric pattern by stitching individual blocks of regular patterns together (Fig. 2f). In the second example, we modified two sites of the triangular pattern as shown in Fig. 2c, and on each site a vertex was removed and the surrounding 6-arm vertices were replaced with 5-arm or 7-arm vertices to form a larger triangle with an edge length of 54 bp (Fig. 2g). In the third example, we designed variable edge lengths (from 32 bp to 52 bp) in different rows of a triangular pattern (Fig. 2h). In the last example, we progressively increased the latitude edge lengths (from 30 to 70 bp) in a square pattern while keeping the lengths of longitude edges unchanged to obtain a cobweb shaped structure (Fig. 2i). The design details of variable edge lengths are shown in Supplementary Fig. 13 and Supplementary Table 4.

The formation of the 2D arrays was first confirmed by native agarose gel electrophoresis (bottom left panels in Fig. 2a–i, Supplementary Fig. 2, and Supplementary Table 2, assembly yields from 11% to 70%, except for the chimeric pattern with a low assembly yield of 1% due to the structural complexity) and then structural details were characterized by Atomic Force Microscopy (AFM) (bottom right panels in Fig. 2a–i and Supplementary Figs. 3–11).

### Tubes

We then designed and constructed a set of wireframe tubes with 6-arm vertices, including a straight tube, a cyclized tube (donut) and three bent tubes. The curvatures in the donut and bent tubes were generated by introducing shorter concave edges and longer convex edges (Supplementary Figs. 14 and 15). The formation of the tubes was confirmed by native agarose gel electrophoresis (Supplementary Fig. 17 and Supplementary Table 5) and the morphologies were characterized by AFM, TEM, and cryo-EM (second and third rows in Fig. 3a–e and Supplementary Figs. 18–32). Reinforcement struts made of simple DNA duplexes were implemented to reduce flexibility of bent tubes (Supplementary Fig. 16). For U-bent (180°-bent) tube as an example, when the number of reinforcement struts increased from 0 to 4 to 8, the distribution of bending angle improved to a sharper peak at the desired angle (Fig. 3c bottom row, angle measurements are shown in Supplementary Table 6). Designs with eight reinforcement struts were hence adopted in 135°- and 90°-bent tubes to achieve more precise angle control. The bending angles of 180°-, 135°-, and 90°-bent tubes with eight struts were measured as 177° ± 6° (mean ± SD, *n* = 100), 137° ± 18° (mean ± SD, *n* = 195) and 104° ± 19° (mean ± SD, *n* = 300) under AFM, respectively.

### Polyhedra

**Fig. 4:**
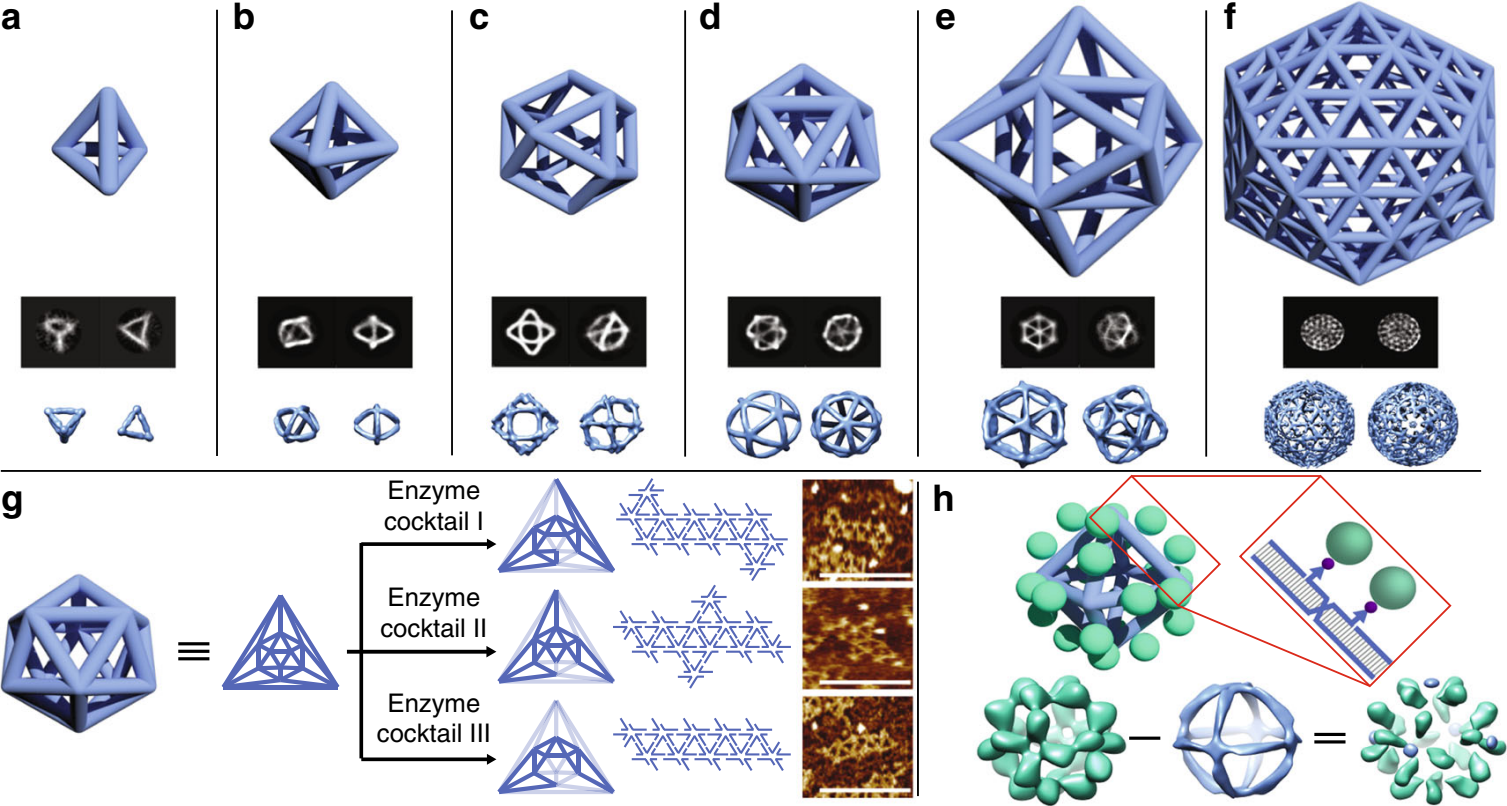
Wireframe polyhedra. **a** Tetrahedron with 3-arm vertices. **b** Octahedron with 4-arm vertices. **c** Cuboctahedron with 4-arm vertices. **d** Icosahedron with 5-arm vertices. **e** Triangulated cube with 6-arm vertices. **f** Triangulated Buckyball with 5-arm and 6-arm vertices. Panels from top to bottom of **a** to **f**: schematic diagrams of polyhedral structures, examples of two-dimensional (2D) classification results and three-dimensional (3D) maps. **g** Enzymatic cleavage of an icosahedron. Panels from left to right: 3D configuration of an icosahedron, Schlegel diagram of an icosahedron, cleavage patterns (light blue edges depicting the edges to be cleaved) for respective enzyme cocktails and the corresponding AFM images. **h** Protein display on an octahedron. Top: schematic diagram of the protein display. Zoomed-in view shows the designated sites of biotin groups (purple) binding to the fusion protein (green); Bottom: 3D maps of octahedron with MBP displayed (left), plain octahedron (middle), and the decorated protein molecules (right) resulted from density subtraction. The details of density subtraction are included in Supplementary Note 1

A variety of polyhedra, such as tetrahedron, octahedron, cuboctahedron, and icosahedron, were generated under the scaffold-free wireframe framework (Fig. 4a–d). In addition to a typical edge length of 32 bp, we also implemented other edge lengths (e.g., 42 and 52 bp) with successful polyhedral formation (Supplementary Figs. 40–42). These polyhedral structures were confirmed by native agarose gel electrophoresis (Supplementary Fig. 33 and Supplementary Table 7). Cryo-EM was then used to characterize the structural details of the polyhedra (see Methods section). The averaged 2D images and reconstructed 3D maps from cryo-EM showed that desired 3D nanostructures self-assembled with good agreement with the designs (Fig. 4a–f and Supplementary Figs. 34–39; Supplementary Table 8). The structural flexibility of the DNA polyhedra increased particle heterogeneity and resulted in relatively low resolution in the corresponding 3D reconstruction results. Taking the set of octahedra of different edge lengths as an example, the increase of edge lengths from 32 bp to 42 bp to 52 bp led to the resolution decay due to the elevated structural flexibility (Supplementary Figs. 40–42). We found that polyhedra without triangular faces (e.g., cube with square faces, and Buckyball with pentagonal and hexagonal faces) were less rigid and prone to deformation. When triangulation was applied to any non-triangular faces, the corresponding structures (e.g., triangulated cube and triangulated Buckyball) showcased significant rigidification (Fig. 4e, f). The set of polyhedra presented in this work range in diameter from 12 to 52 nm with molecular weight from 120 kDa to 5 MDa; the largest being a triangulated Buckyball with 92 vertices, 180 faces, 270 edges. This is an artificial DNA object with the highest geometric complexity to date.

Because the polyhedra are fully addressable with de novo designed DNA sequences, restriction enzyme sites can be incorporated at any given edges. To demonstrate controlled enzymatic cleavage, we programmed 13 restriction enzyme recognition sites for six unique restriction enzymes into a DNA icosahedron (Supplementary Fig. 43). The samples were treated with variable restriction enzyme cocktails and AFM images of the resulting structures revealed the expected patterns (Fig. 4g and Supplementary Figs. 43–48).

The DNA polyhedra can be used to display protein molecules at designated sites. As an example, maltose-binding protein (MBP) was fused with engineered streptavidin monomer to bind to biotin groups placed at the chosen sites of an octahedron (two sites per edge facing outside of the octahedron). Through the strong affinity between the biotin groups and fused streptavidin monomers, the fusion protein molecules were displaced from the octahedral surface in a controlled fashion. In comparing the 3D density maps between the octahedrons with and without protein displayed, extra densities corresponding to the fused protein molecules were identified (Fig. 4h and Supplementary Fig. 49), clearly indicating the desired positioning and orientation.

### Three-dimensional (3D) arrays

**Fig. 5:**
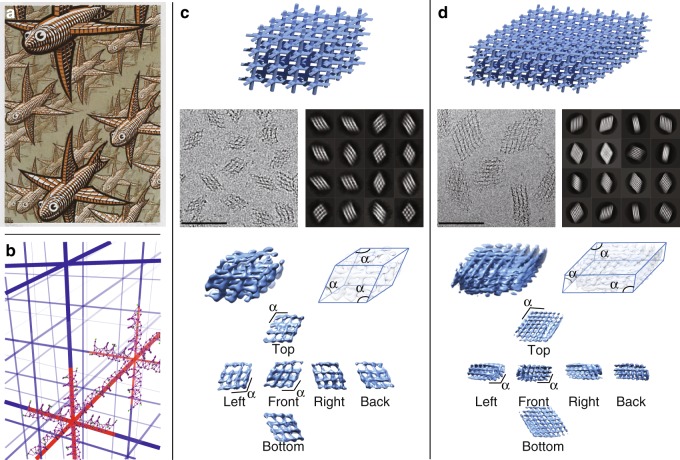
Three-dimensional (3D) multi-layer arrays with 6-arm vertices. **a** Escher’s woodcut *Depth*. **b** DNA duplexes (highlighted in red) overlaid on a grid analog to *Depth*. **c** 4 × 4 × 4 array. **d** 8 × 8 × 4 array. Panels from top to bottom (**c** and **d**): Schematic diagrams; cryo-EM images (left) and examples of two-dimensional (2D) classification results (right); 3D maps, and different views of 3D electron density maps. (M.C. Escher’s *Depth* © 2019 The M.C. Escher Company—the Netherlands. All rights reserved. Used by permission. www.mcescher.com)

The scaffold-free wireframe framework can also be readily applied to build fully addressable 3D arrays (‘‘nanocrystals’’), which are challenging with the DNA origami approach due to both the scaffold length limitations and routing difficulty. The 3D configuration of 6-arm vertices of a ‘‘nanocrystal’’ in this study dwelt in the same design spirit to what was proposed by Seeman at the dawn of DNA nanotechnology, which was inspired by Escher’s woodcut *Depth* (Fig. 5a)^37^. A repeating fish array was presented in *Depth*, with the head of each fish aligned towards the front, tail towards the back, and four fins pointing top, bottom, left, and right. A 6-arm branching vertex is analogous to the periodic 3D orientation of fish depicted in *Depth* (Fig. 5a, b).

A 4(vertex) × 4(vertex) × 4(vertex) array was produced with four virtual layers of 4 × 4 6-arm vertices with arms branching out to the front, back, left, and right connected in respective layer, while inter-layer connection resulted from association of arms branching out to the top and bottom. Besides the 4 × 4 × 4 array, we also produced an 8 × 8 × 4 array, which is four times as large as a typical origami structure, to demonstrate scalability. 3D array formation was confirmed by native agarose gel electrophoresis (Supplementary Fig. 53 and Supplementary Table 9), while morphologies were confirmed by AFM and TEM (Supplementary Figs. 59–62). Details of the parallelepiped mesh structures were revealed by cryo-EM (Fig. 4c, d, Supplementary Figs. 54 and 55, and Supplementary Table S10). Three-dimensional (3D) maps (Fig. 4c, d and Supplementary Figs. 50, 54, and 55) indicate that 4 × 4 × 4 and 8 × 8 × 4 arrays are more rhombohedral (*α* = 114–115°) than cubic. The 3D reconstruction has limited resolution (30 Å for 4 × 4 × 4 array and 38 Å for 8 × 8 × 4 array), likely resulting from sample inhomogeneity due to the structural flexibility of the 3D DNA arrays.

Likewise, we designed a diamond cubic array and a cross-like array with two types of 4-arm vertices, respectively. The diamond cubic array consisted of four layers of 7 × 4 4-arm vertices with arms of each vertex branching out tetrahedrally. Three of the four arms of a vertex connect to neighboring vertices of the same layer and the fourth arm branching out upward or downward to connect to a vertex of another layer (Supplementary Fig. 51). The cross-like array consisted of four layers of 7 × 4 planar 4-arm vertices of two classes of branching orientations in alternating columns. The four arms of a vertex in the horizontal column and two arms branching out front and back of a vertex in the vertical column connect to neighboring vertices within the same layer and the other two arms branching out upward and downward of a vertex in the vertical column connect to vertices of another layers (Supplementary Fig. 52). Successful self-assembly was verified by both agarose gel electrophoresis (Supplementary Fig. 53 and Table S9) and cryo-EM imaging (Supplementary Figs. 56 and 57). However, the four arms around each vertex were poorly confined with less preserved geometry, and hence the 3D reconstruction from cryo-EM imaging was extremely challenging for these two types of structures.

## Discussion

All wireframe structures made in this work are fully addressable. The largest 2D array, the largest 3D polyhedron, and the largest 3D array are composed of 654, 540, and 1536 strands of distinct sequences, respectively. The material efficiency of the porous wireframe structures is higher than the canonical compact DNA lattice design (Supplementary Table 11) for occupying the same area or volume. For example, scaffold-free approach already gives rise to an addressable cube structure composed of more than 33,000 distinct strands and such a massive structure could easily obtain fivefold volume expansion when migrating to wireframe framework with similar complexity. When further scaling up the self-assembly of wireframe structures from short strands, we might face the familiar challenge of using simple branched molecules to make crystals. With a better understanding of structural flexibility and long-range propagation of deformation, it might be possible to generate larger ‘‘nanocrystals’’ with addressable components. Moreover, this porosity also provides effective scaffolding for hosting guest molecules of interest (e.g., host protein molecules for structural determination)^20,28^.

The morphology and rigidity of the wireframe structures rely heavily on the connectivity pattern. We have shown that triangulation is an effective approach to rigidify the corresponding structures. The marshmallow-like polyhedra without triangular faces gained substantial structural rigidity after triangulation arrangements.

The structural flexibility indicates that the resulting structures are dynamic and ready to reconfigure when desired stimuli are available. For example, higher magnesium ion concentration leads to stronger stacking for neighboring bases across branching arms and alleviates the repulsion of adjacent DNA helices^38–40^. Consequently, a tightening trend was found with increasingly higher magnesium ion concentrations for the 2D array of 4-arm vertices (Supplementary Fig. 63 and Supplementary Table 12). With a large toolbox of dynamic elements already available^41^ and a natural working interface with other biomolecules, one can imagine sophisticated dynamic structures and devices to be engineered under such a design scheme.

The successful self-assembly in this study, together with works from recent studies, have shown that addressable mega-Dalton structures can self-assemble from a wide range of building blocks, including single-stranded tiles/bricks, double crossover motifs and derivatives, and junction motifs. We hope this work can kindle a rediscovery of rich collection of classic DNA motifs to open up new possibilities in self-assembly^26,32,42–45^.

## Methods

### DNA sequence design

With a general connectivity pattern and segmentation scheme laid out, a python script was written to generate a xml file in which a specific segment pairing map and other strand information (e.g., strand lengths and sequence exclusion) were defined. Such a xml file was used as the input file for sequence generation software Uniquimer^46^ to generate a full list of component strands of a certain wireframe structure. Then DNA sequences were generated by Uniquimer using the following rules: (1) Nucleotides (that is, A, C, G, and T) are randomly generated one by one along each oligonucleotide chain. (2) Complementary nucleotides to ones generated in (1) are matched following the base-pairing rule: A to T and vice versa; C to G and vice versa. (3) No repeating-segment beyond a certain length (seven or eight nucleotides) is allowed. When such repeating segments appear during design, the most recently generated nucleotides will be mutated until the repeating-segment requirement is satisfied. (4) No four consecutive A, C, G, or T bases are allowed. Extra unpaired base(s) (e.g., 1T, 2T, or 3T) were manually designed and added to certain strands after sequences were appointed. DNA sequences can be found in Supplementary Data 1.

### Structural assembly

DNA oligonucleotides were synthesized by Integrated DNA Technology Incorporation or Bioneer Corporation and were used without further purifications. To assemble desired structures, component strands were mixed at a roughly equal molar final concentration of 100–300 nM per strand, in 0.5 × TE buffer (5 mM Tris, pH = 7.9 and 1 mM EDTA) or 1 × TAE buffer (40 mM Tris, pH = 8.0, 20 mM acetic acid, and 1 mM EDTA), supplemented with 15–40 mM MgCl_2_. The DNA mixture was then annealed with a ‘ramp’’ annealing program cooling down from 90 to 25 °C (or 10 °C) over a period of 17–76 h for all the structures except the 8 × 8 × 4 array (mixture was annealed in a 37 °C water bath for about 3 weeks). Additional details can be found in Supplementary Methods.

### Gel electrophoresis and yield quantification

Annealed samples were subjected to 1% or 2% native agarose gel electrophoresis in an ice-water bath, and gels were prepared in 0.5 × TBE buffer (44.5 mM Tris, 44.5 mM boric acid, and 1 mM EDTA) with 10 mM MgCl_2_ and pre-stained with SYBR Safe (Thermo Fisher Scientific). To purify desired structures, target bands were excised and finely crushed in a Freeze’N Squeeze column (Bio-Rad), and then directly subjected to centrifugation at 438 × *g* for 3 min at 4 °C. Samples centrifuged through the column were collected for further analysis by AFM/TEM/cryo-EM.

To quantify the assembly yield, the intensity of the target band was compared against a standard band^17^ (e.g., 1500-base-pair band from a 1-kb DNA ladder mixture). The mass value of the target band was deduced from the intensity–mass correlation based on the standard band, and was used to calculate the yield of the desired structure.

### AFM imaging

AFM images were obtained using a SPM Multimode with Nanoscope V controller (Bruker). Forty microliters of 0.5 × TE buffer with 10 mM MgCl_2_ were applied to a freshly cleaved mica surface, and then a 5 μL droplet of purified sample (2–10 nM) was added and incubated for approximately 2 mins. Supplementary 10 μL of 10 mM NiCl_2_ was added to increase the strength of DNA–mica binding^47^. Additional dilution of the sample was possibly performed to achieve the desired sample density. Samples were imaged under liquid ScanAsyst mode, with C-type triangular tips (resonant frequency, *f*_0_ = 40–75 kHz; spring constant, *k* = 0.24 N m^−1^) from the SNL-10 silicon nitride cantilever chip (Bruker). Additional details of measurements based on AFM results can be found in Supplementary Information.

### TEM imaging

A 3.5 μL droplet of purified sample (2–10 nM) was applied to a plasma-treated, carbon-coated grid (Electron Microscopy Sciences) for 4 min and then wicked off and stained for 5 s with 3.5 μL of stain buffer (2% aqueous uranyl formate with 25 mM). Then stain buffer was blotted off by filter paper and left on the grid to be air-dried. The stained sample was analyzed by FEI Tecnai Spirit, operated at 120 kV at 26,000 to  × 63,000 magnification.

### Cryo-EM imaging

Freshly purified samples of DNA polyhedra or 3D arrays were applied onto lacey carbon grids (Ted Pella) pre-treated with 0.1 M MgCl_2_. The grids were blotted for approximate 4–7 s then frozen in liquid ethane using a cryo-plunger (Cryo-Plunger 3, Gatan). Micrographs of the DNA octahedron samples were collected using an FEI Titan Krios microscope operating at 300 kV with a Falcon II camera (FEI) in movie mode. Micrographs of other DNA polyhedra samples were collected using a FEI Tecnai Arctica operating at 200 kV with a Falcon II camera (FEI) in movie mode. A total of 88 micrographs for the DNA tetrahedron, 434 micrographs for the DNA octahedron, 352 micrographs for the DNA cuboctahedron, 184 micrographs for the DNA icosahedron, 223 micrographs for the DNA triangulated cube, 410 micrographs for the triangulated Buckyball, 120 micrographs for the protein decorated octahedron, 569 micrographs for the 4 × 4 × 4 arrays and 1978 micrographs for the 8 × 8 × 4 arrays were collected.

### Single-particle reconstruction

Raw images were processed by using the program MotionCorr^48^ to align and combine the movie stacks. CTF parameters were determined by using the program Ctffind4^(^^49^^)^. Particles were automatically picked with the program e2boxer of the EMAN2 package3. The automatically picked particles were then manually checked and adjusted. The boxed images were applied for 2D classification using the program RELION1.4 or RELION2.0^(^^50^^)^. Particles were selected by visual inspections with the 2D average images calculated using the images in each class. Initial models were generated from the 2D average images by using the python script e2initialmodel.py of the EMAN2 package. The generated initial models were used as references for 3D auto refine with RELION1.4 or RELION2.0. The models generated from the 3D auto refinements were then used as references for 3D classifications. The particles were further selected from the best classes of the 3D classification results. The selected particles were then used for the final 3D refinements yielding the final reconstructions.

### Restriction enzyme cleavage

The annealed DNA icosahedron samples were treated with different restriction enzyme cocktails for corresponding patterns (BsiWI-HF, BstZ17I-HF, MfeI-HF, MluI-HF for pattern I; AclI, BamHI-HF, MfeI-HF, MluI-HF for pattern II; AclI, BamHI-HF, BsiWI-HF, BstZ17I-HF, MfeI-HF, MluI-HF for pattern III). One unit of each enzyme was used to digest the icosahedron in 50 µL 1 × CutSmart buffer at 37 °C for 2 h. Additional details can be found in Supplementary Methods.

### Code availability

Code used in this study is available from the authors upon reasonable request.

## Supplementary information

Supplementary Information

Supplementary Movie 1

Supplementary Movie 2

Supplementary Movie 3

Supplementary Movie 4

Supplementary Movie 5

Supplementary Movie 6

Supplementary Movie 7

Supplementary Movie 8

Supplementary Movie 9

Supplementary Movie 10

Supplementary Movie 11

Description of Additional Supplementary Files

Supplementary Data 1

## Data Availability

All data supporting the findings of this study are included in the paper and its Supplementary Information or are available from the authors upon reasonable request
