# Supplementary material for: Complex wireframe DNA nanostructures from simple building blocks
Source: Nat Commun. 2019 Mar 6;10:1067. doi: 10.1038/s41467-019-08647-7 (PMC6403373; doi:10.1038/s41467-019-08647-7)
Supplement: Supplementary file 1 — Supplementary Information [file 41467_2019_8647_MOESM1_ESM.pdf]

Supplementary Information for
Wireframe DNA nanostructures composed of short synthetic strands

Wen Wang[1, †], Silian Chen[2, 3, †], Byoungkwon An[4], Kai Huang[1], Tanxi Bai[1], Mengyuan Xu[1], Gaëtan Bellot[5], Yonggang Ke[6], Ye Xiang[2, *], Bryan Wei[1, *]

Correspondence to: bw@tsinghua.edu.cn (B.W.); yxiang@tsinghua.edu.cn (Y.X.)
[†]These authors contributed equally.

This PDF file includes:

Supplementary Figures 1 to 63
Supplementary Tables 1 to 12
Supplementary Notes 1 to 3
Supplementary Reference

Other Supplementary Information for this manuscript include the following:

Sequences
Supplementary Movies 1 to 11

Supplementary Figures

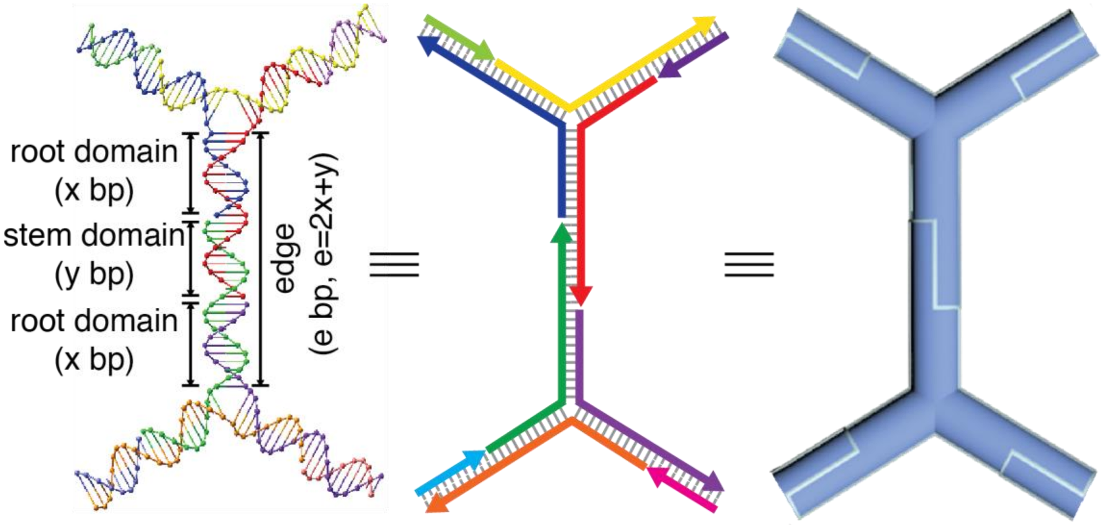

Supplementary Figure 1. Strand diagrams of a typical edge segmentation. In this study, a typical edge (e bp) is segmented into two root domains of the same lengths (x bp) and a stem domain (y bp).

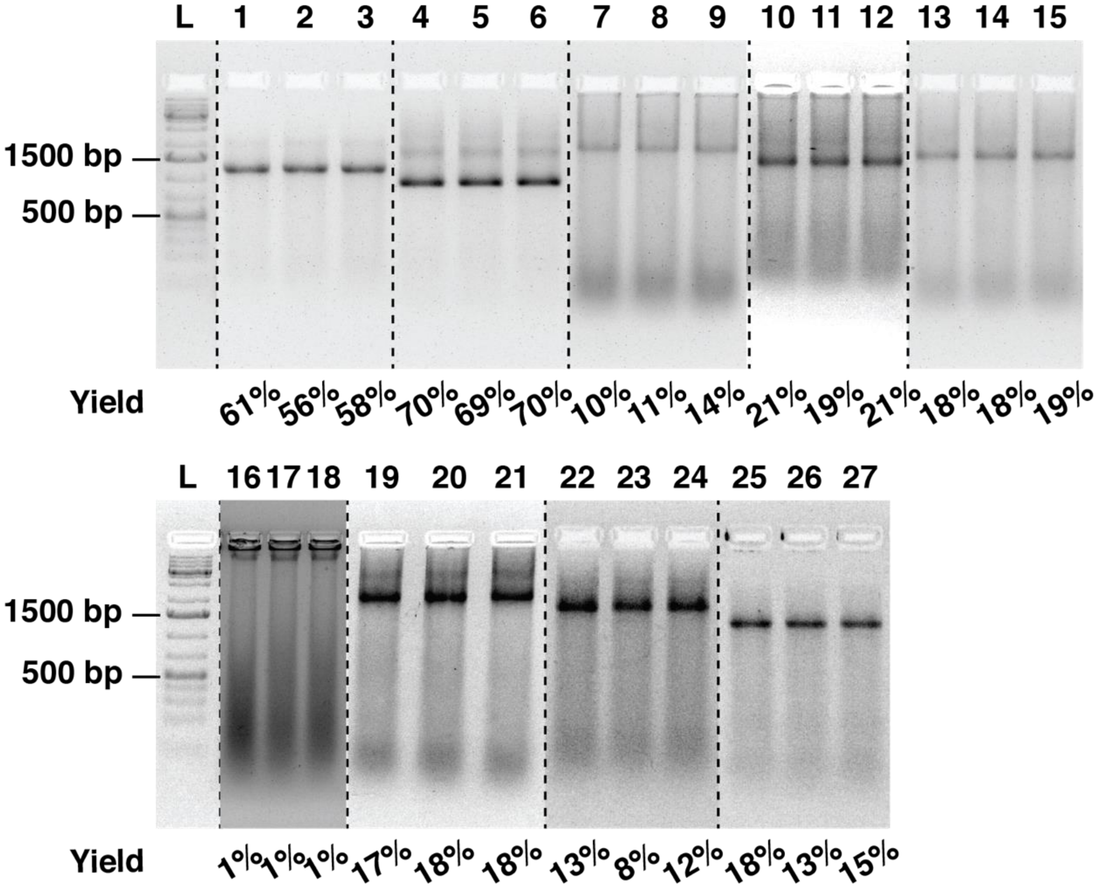

Supplementary Figure 2. Native agarose gel electrophoresis analysis for different tessellation patterns. Lane L: 1-kb DNA ladder; lanes 1-3: three replicates of hexagonal tessellation pattern with 3-arm vertices; lanes 4-6: three replicates of square tessellation pattern with 4-arm vertices; lanes 7-9: three replicates of triangular tessellation pattern with 6-arm vertices; lanes 10-12: three replicates of trihexagonal tessellation pattern with 4-arm vertices; lanes 13-15: three replicates of snub trihexagonal tessellation pattern with 5-arm vertices; lanes 16-18: three replicates of chimeric pattern with individual blocks composed of 3-arm, 4-arm

and 6-arm vertices; lanes 19-21: three replicates of irregular triangular pattern 1 with 6-arm vertices; lanes 22-24: three replicates of irregular triangular pattern 2 with 6-arm vertices; 25-27: three replicates of cobweb-like pattern with 4-arm vertices. Numbers at the bottom indicate assembly yields. (Results were merged from multiple gel images.)

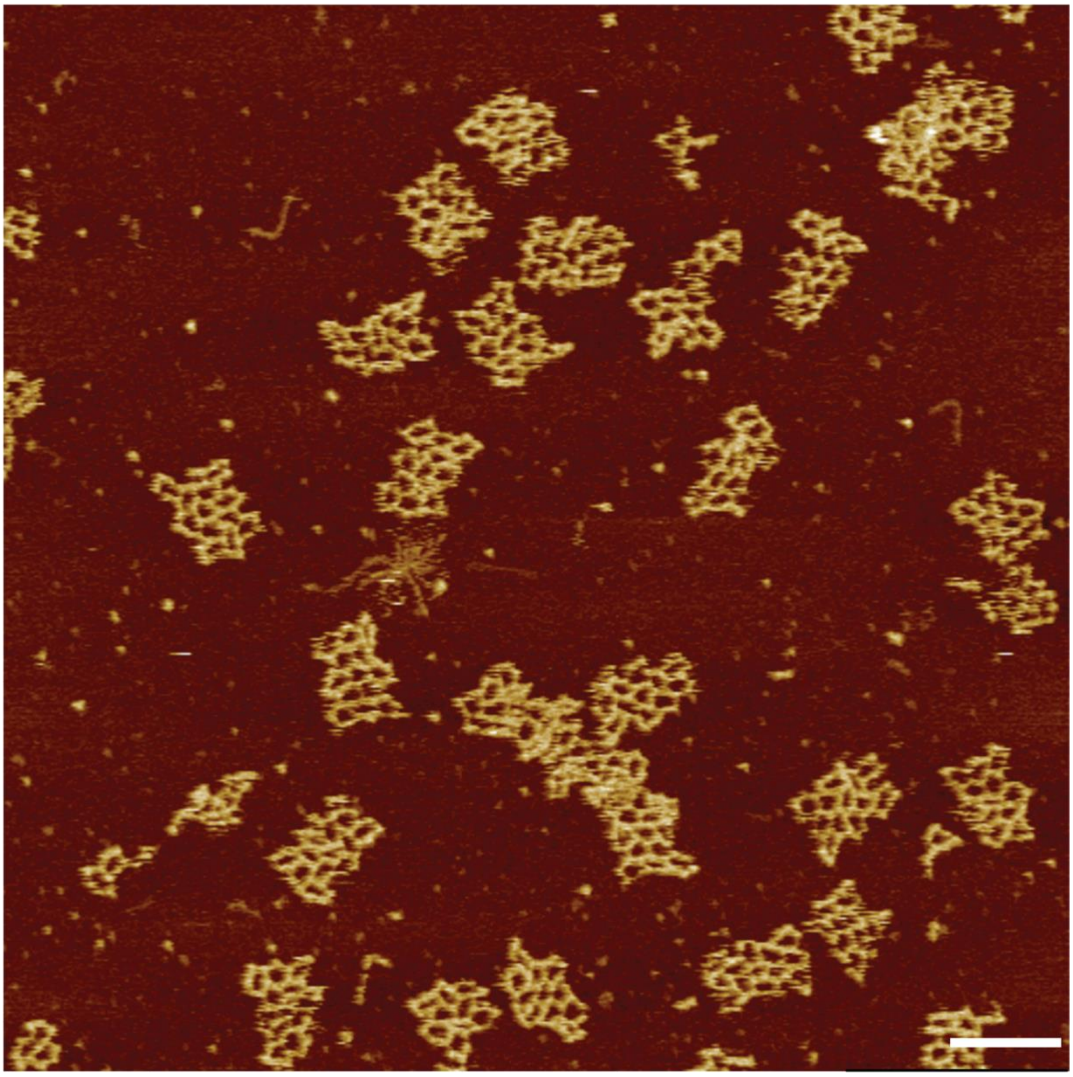

Supplementary Figure 3. Full size AFM image of hexagonal tessellation pattern with 3-arm vertices (scale bar: 100 nm).

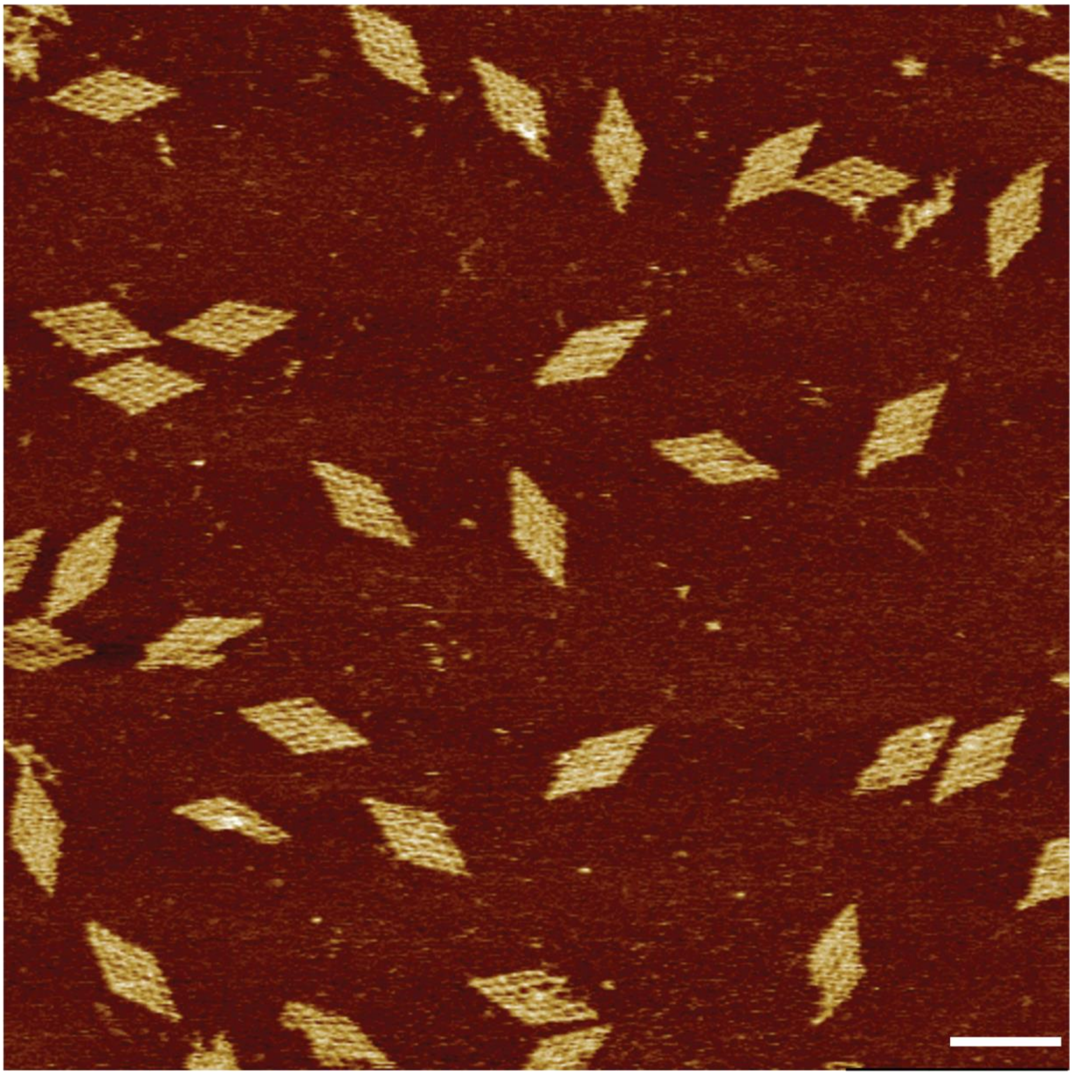

Supplementary Figure 4. Full size AFM image of square tessellation pattern with 4-arm vertices (scale bar: 100 nm).

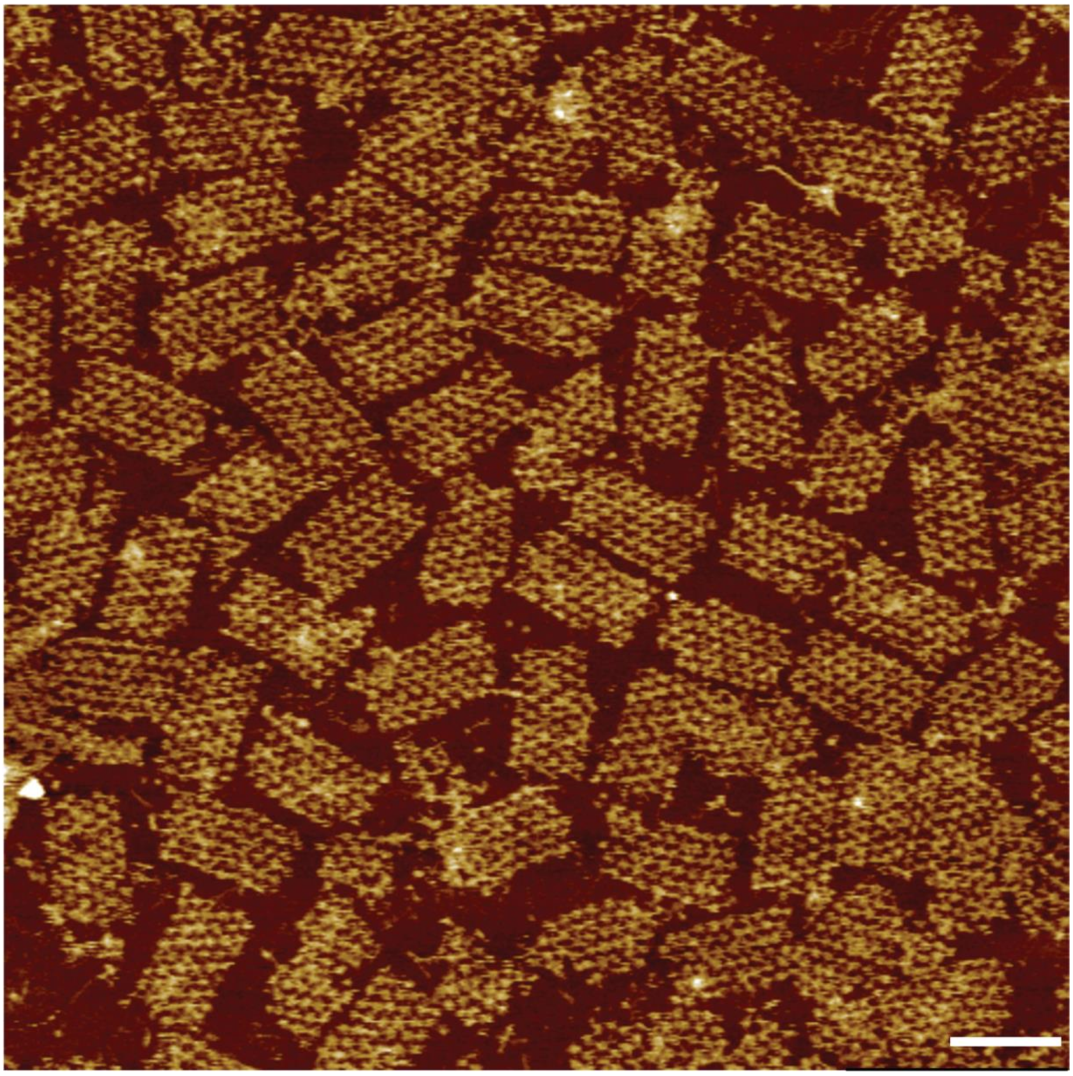

Supplementary Figure 5. Full size AFM image of triangular tessellation pattern with 6-arm vertices (scale bar: 100 nm).

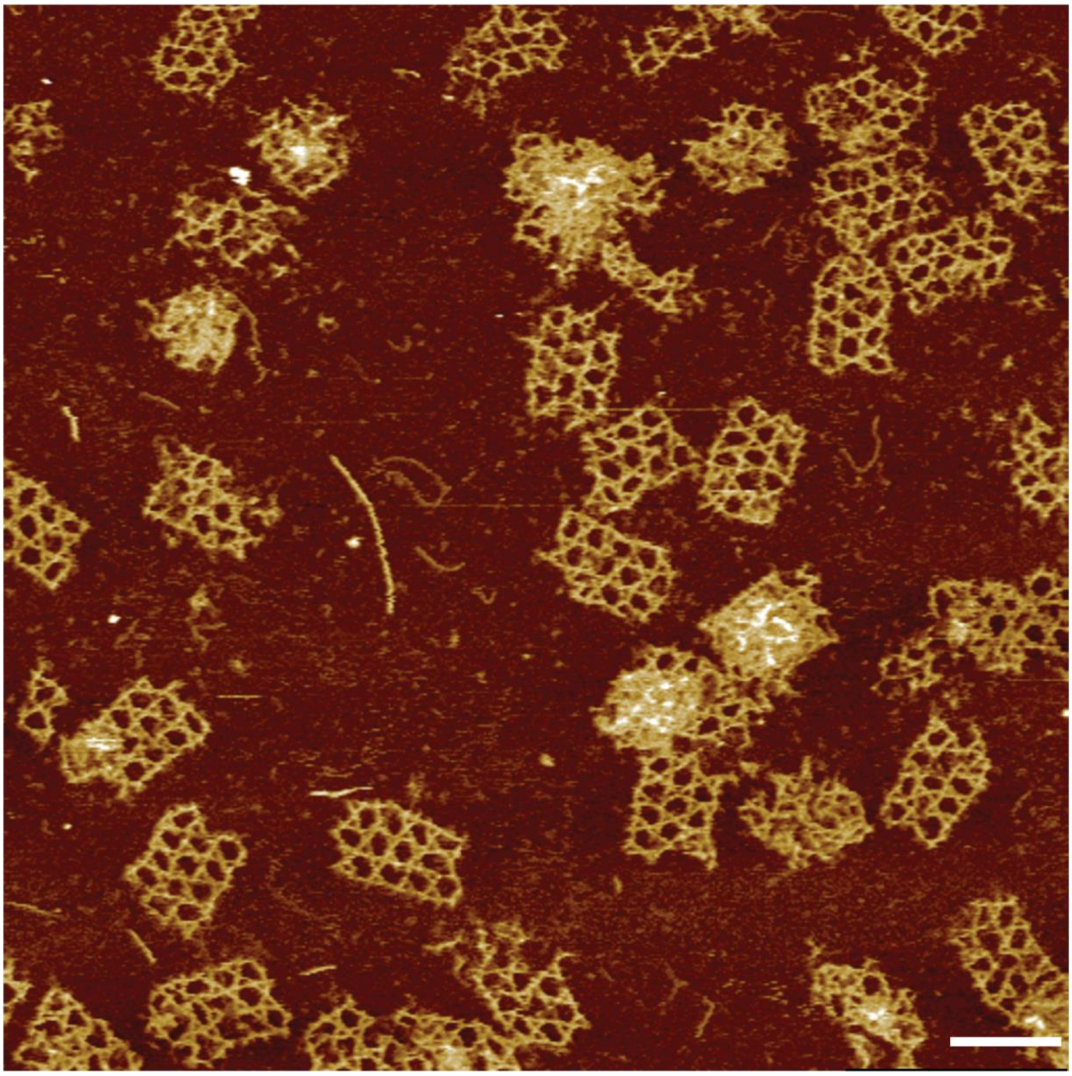

Supplementary Figure 6. Full size AFM image of trihexagonal tessellation pattern with 4-arm vertices (scale bar: 100 nm).

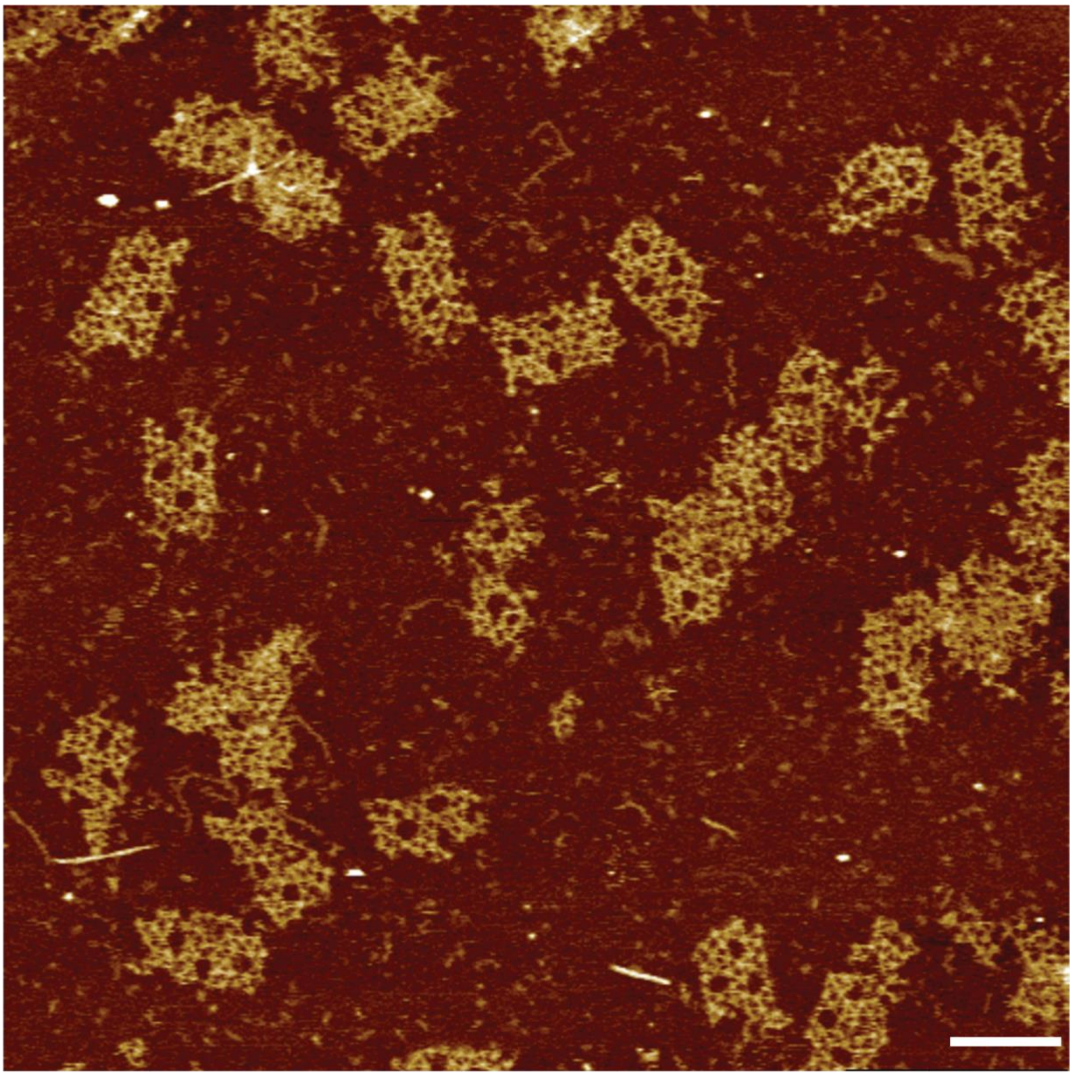

Supplementary Figure 7. Full size AFM image of snub trihexagonal tessellation pattern with 5-arm vertices (scale bar: 100 nm).

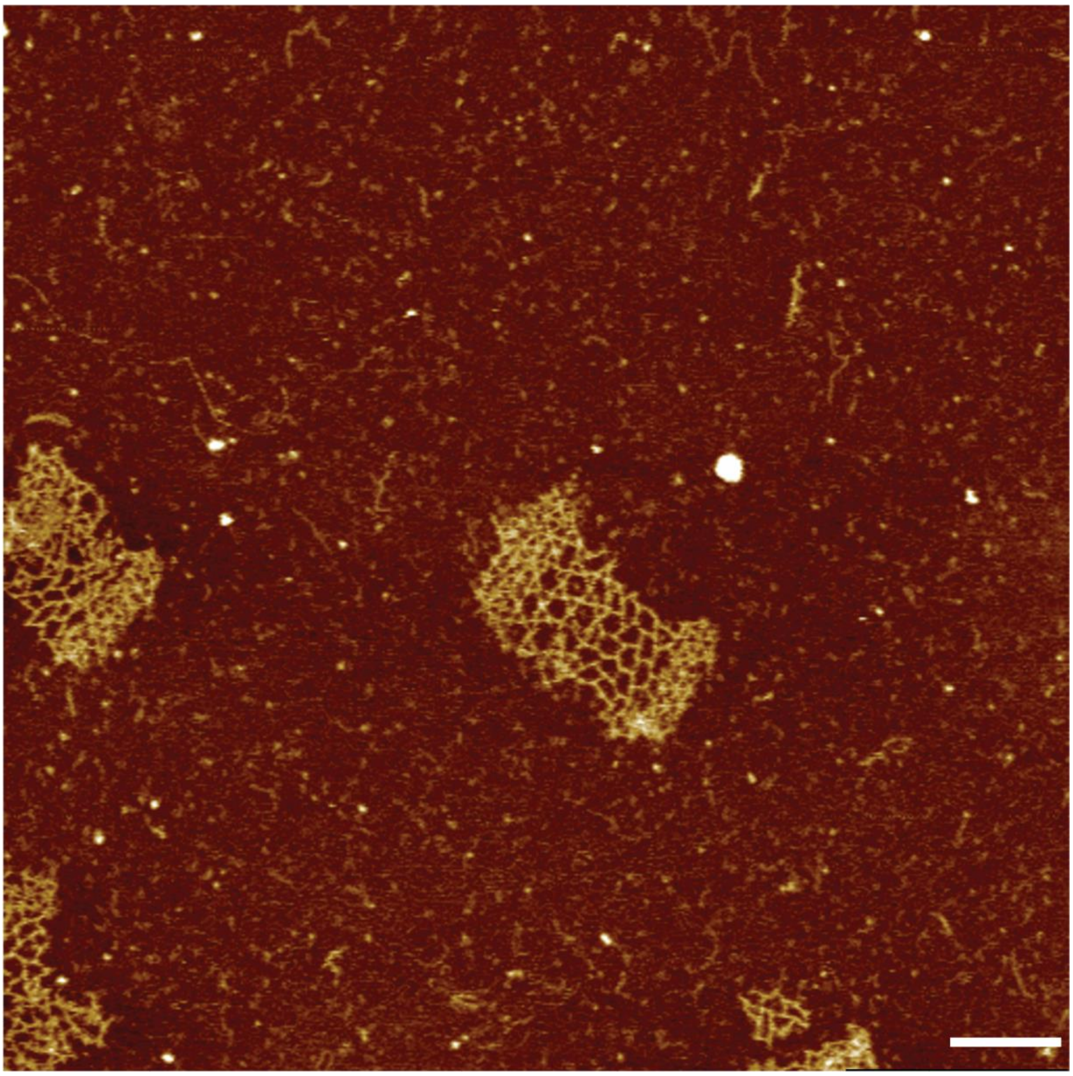

Supplementary Figure 8. Full size AFM image of chimeric pattern with individual blocks composed of 3-arm, 4-arm and 6-arm vertices (scale bar: 100 nm).

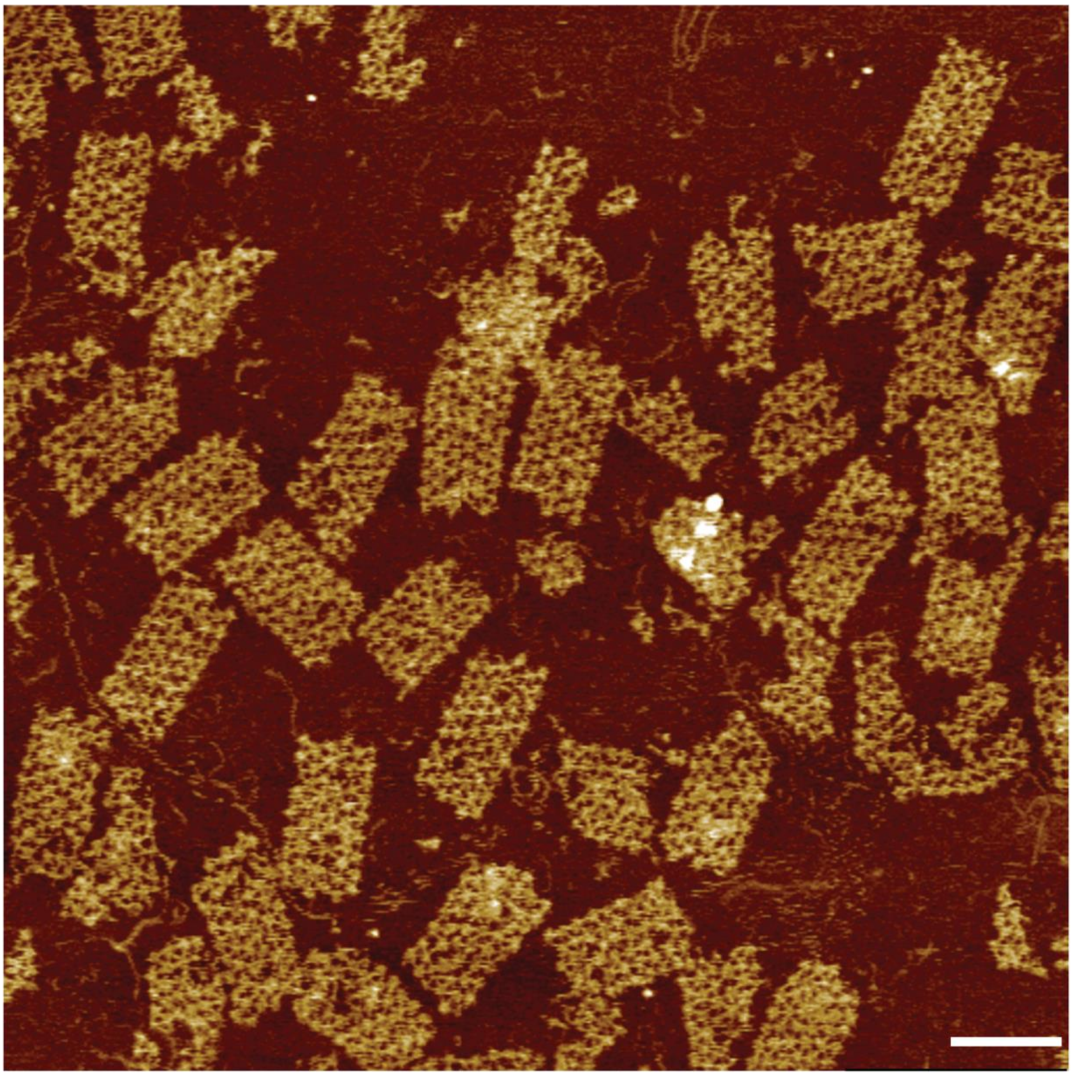

Supplementary Figure 9. Full size AFM image of irregular triangular pattern 1 with 6-arm vertices (scale bar: 100 nm).

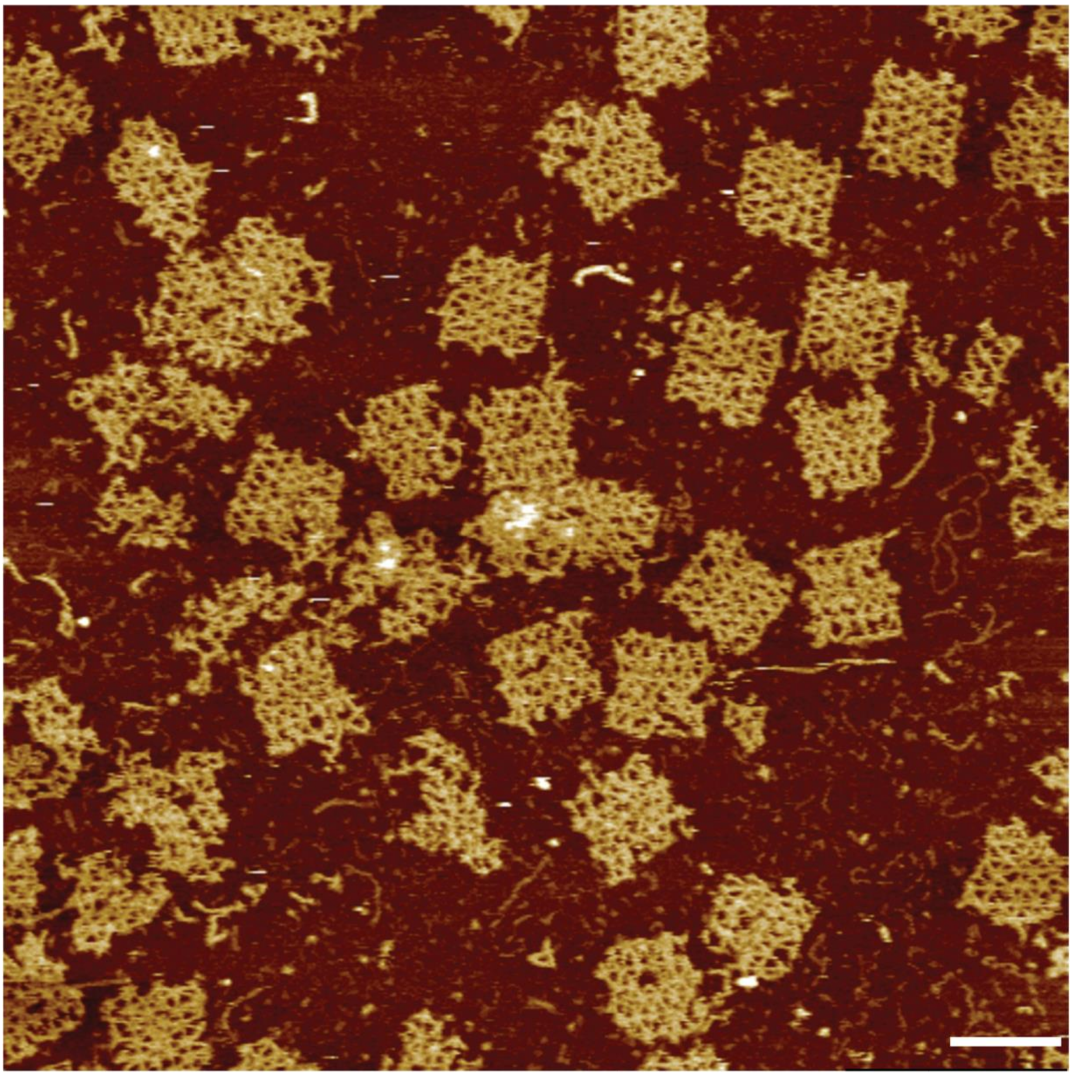

Supplementary Figure 10. Full size AFM image of irregular triangular pattern 2 with 6-arm vertices (scale bar: 100 nm).

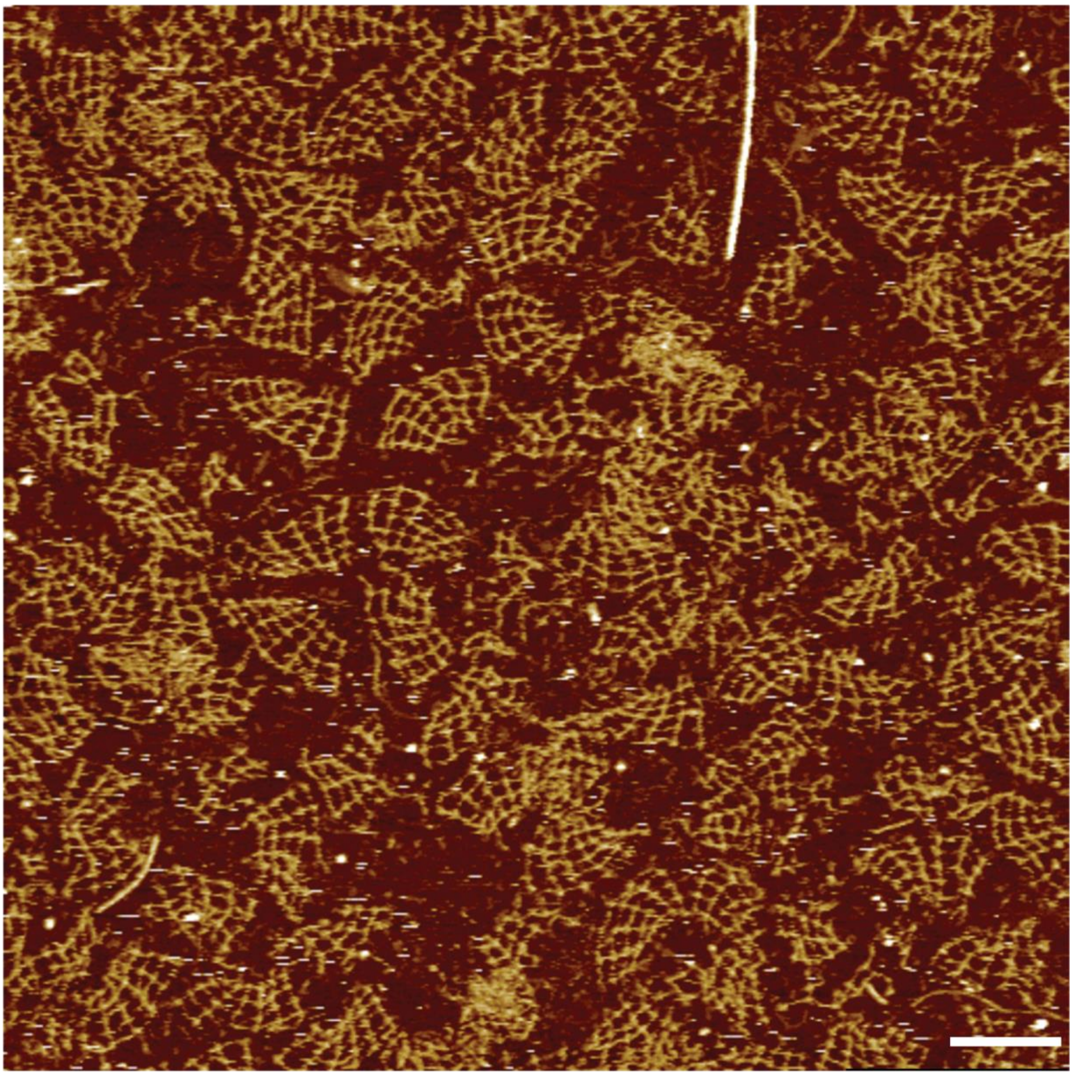

Supplementary Figure 11. Full size AFM image of cobweb-like pattern with 4-arm vertices (scale bar: 100 nm).

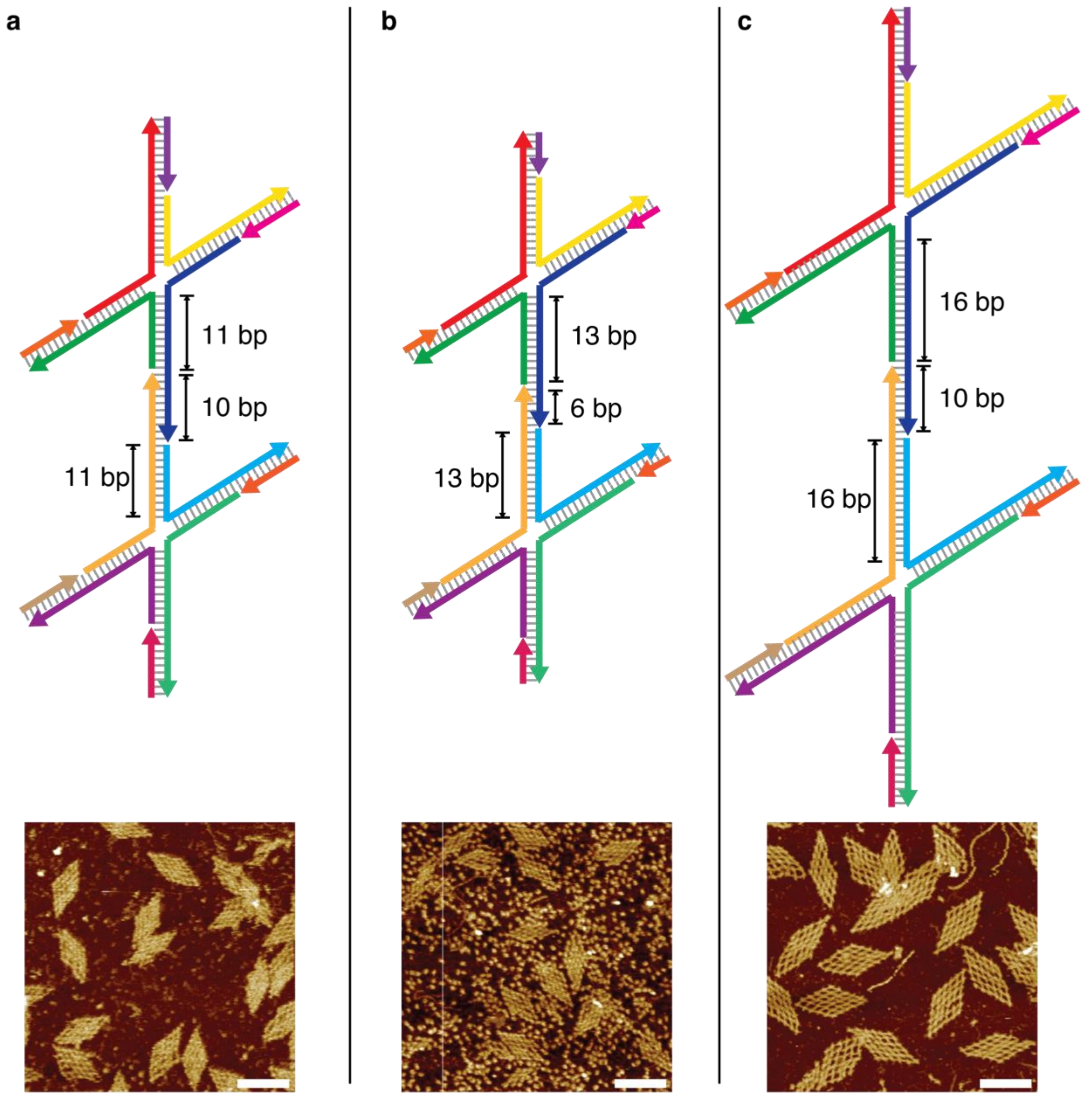

Supplementary Figure 12. Schematic diagrams of 4-arm vertices with different domain lengths and AFM images of their corresponding square tessellation patterns. (a) Square tessellation pattern formed by 4-arm vertices with 11-bp root domains and 10-bp stem domains. (b) Square tessellation pattern formed by 4-arm vertices with 13-bp root domains and 6-bp stem domains. (c) Square tessellation pattern formed by 4-arm vertices with 16-bp root domains and 10-bp stem domains. Panels from top to bottom: schematic diagrams of 4-arm vertices with different domain lengths and AFM images of the corresponding tessellation patterns (scale bars: 100 nm).

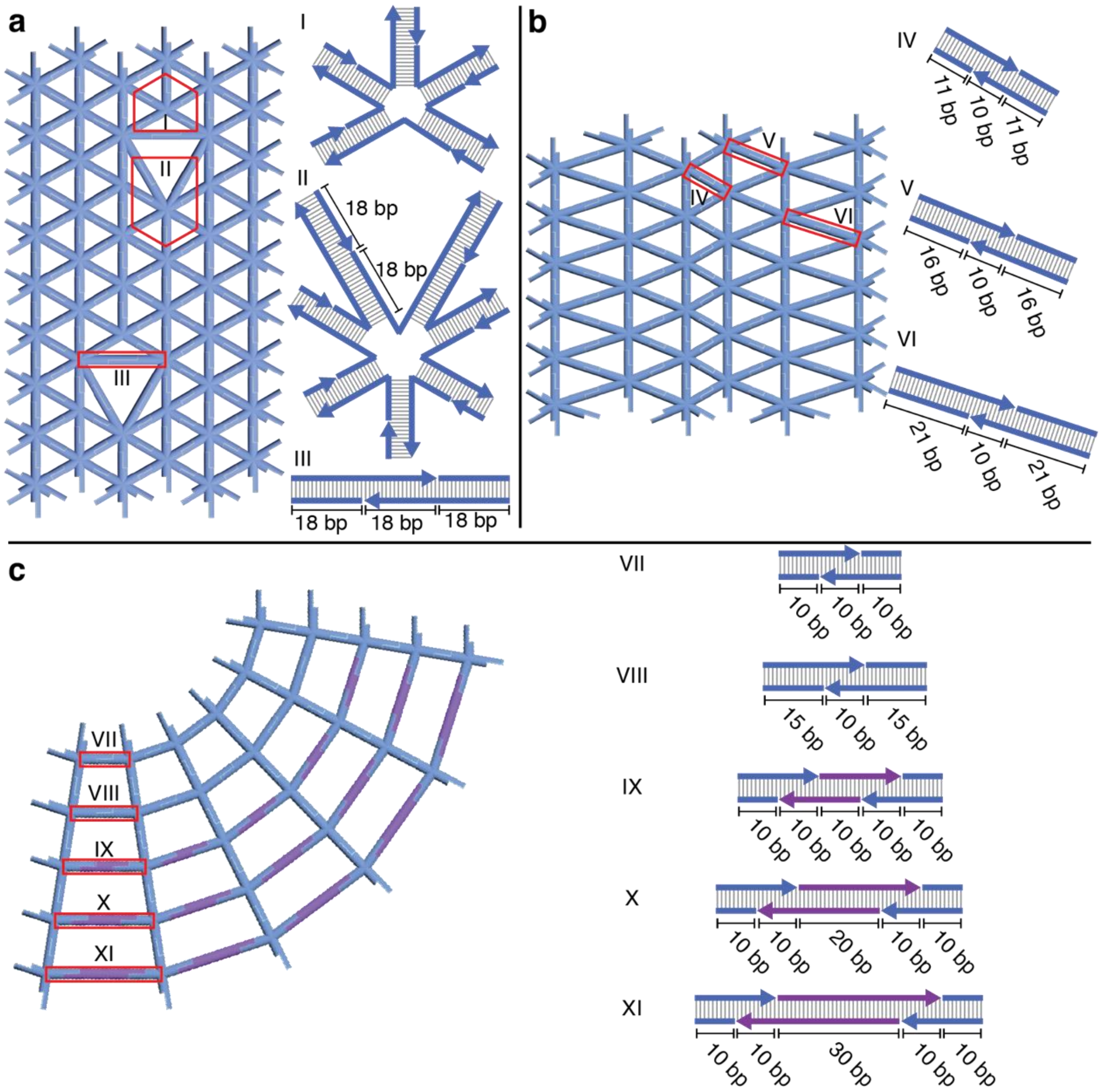

Supplementary Figure 13. Detailed designs of irregular tessellation patterns. (a) Irregular triangular pattern 1 with 6-arm vertices. Insets I and II show the strand diagrams of a 5-arm vertex and a 7-arm vertex that replace 6-vertices to form the irregular patterns. Inset III shows the domain lengths of a 54-bp edge. Domain lengths besides 11-bp root domains and 10-bp stem domains were indicated by numbers. (b) Irregular triangular pattern 2 with 6-arm vertices. Insets IV, V and VI show the strand diagrams of edges with lengths of 32-bp, 42-bp and 52-bp, respectively. (c) Cobweb-like pattern with 4-arm vertices (splinter segments shown in purple). Insets VII to XI show the strand diagrams of edges with lengths ranging from 30 bp to 70 bp, respectively.

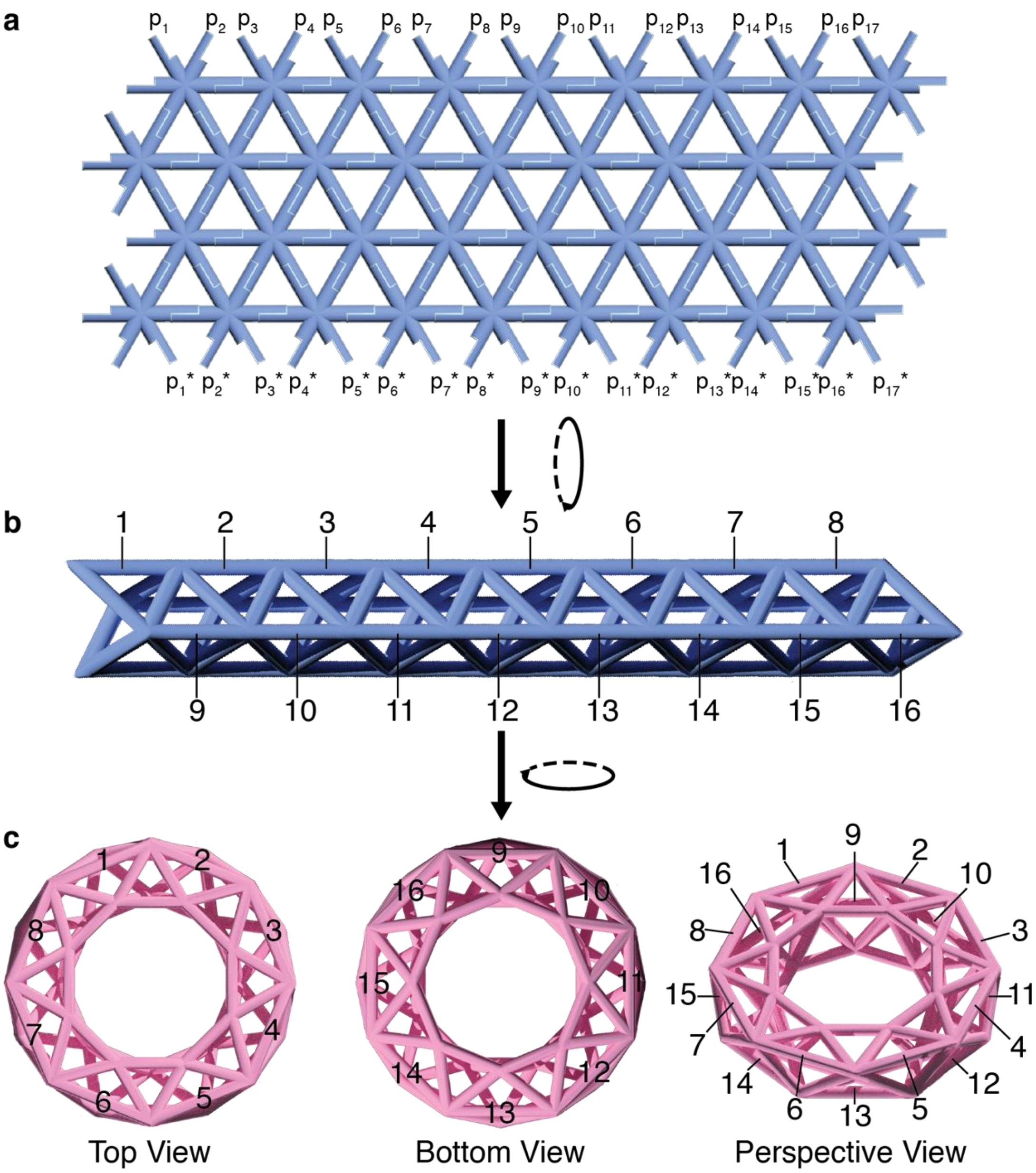

Supplementary Figure 14. A morphing from triangular tessellation pattern into straight tube and from straight tube into donut. A 9×4 structure of triangular tessellation pattern with 6-arm vertices (a) can be rolled up to form a straight tube (B) by cyclizing the top and bottom boundaries via the base pairings of the sticky ends p_i with p_i^*. By carefully adjusting the lengths of specific edges labeled with numbers from 32 bp to 52 bp, a straight tube (b) is morphed into a donut (c). Positions of 52-bp edges are labeled with numbers and can be found in the top, bottom and perspective views of a circular tube in (c).

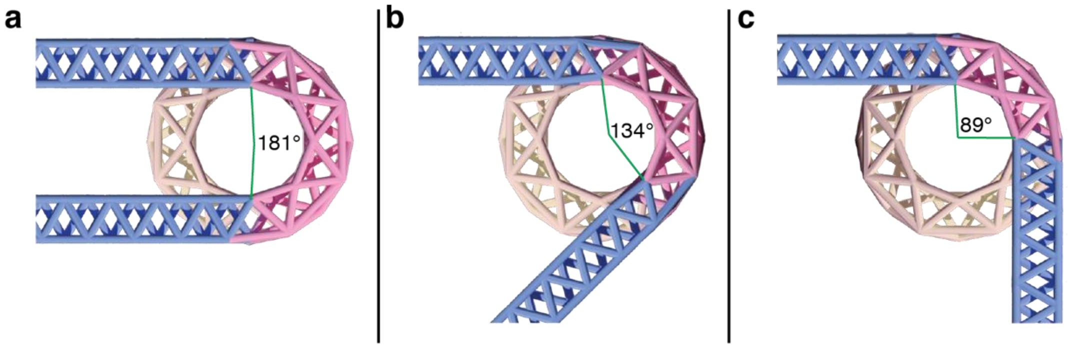

Supplementary Figure 15. Specific parts of donut (pink) taken to link two tubes to form the bent tubes with designated angles. (a) U-bent tube. (b) 135°-bent tube. (c) 90°-bent tube.

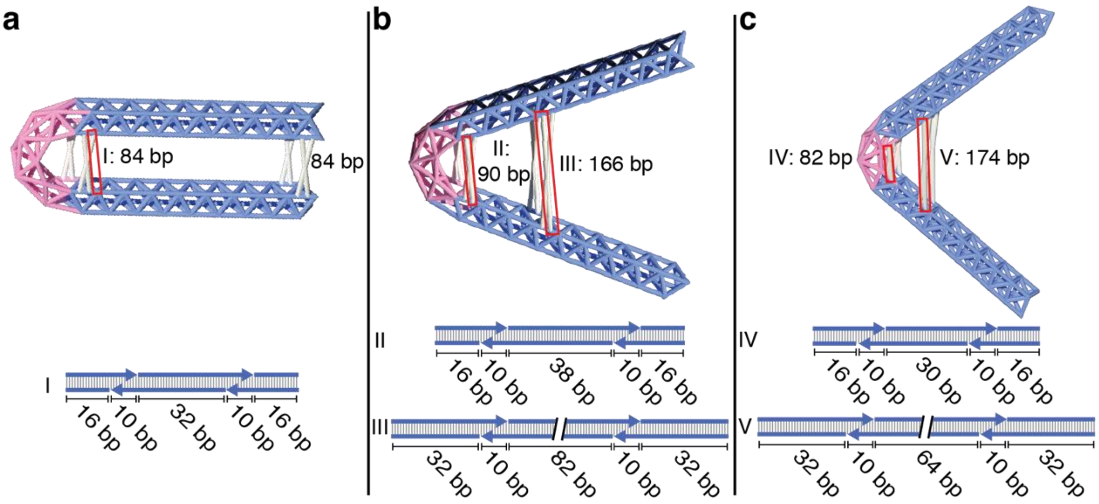

Supplementary Figure 16. Schematic diagrams and strand diagrams of reinforcement struts for (a) U-bent tube, (b) 135°-bent tube and (c) 90°-bent tube. Insets under the bent tubes show the strand diagrams of the corresponding struts structures.

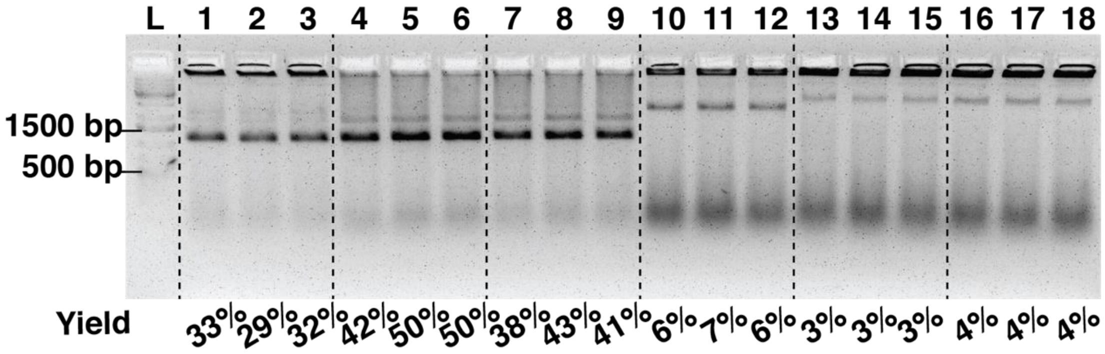

Supplementary Figure 17. Native agarose gel electrophoresis analysis for straight tube, donut and bent tubes. Lane L: 1kb DNA ladder; lanes 1-3: three replicates of donut; lanes 4-6: three replicates of straight tube 1; lanes 7-9: three replicates of straight tube 2; lanes 10-12: three replicates of U-bent tube; lanes 13-15: three replicates of 135°-bent tube; lanes 16-18: three replicates of 90°-bent tube. Numbers at the bottom indicate assembly yields.

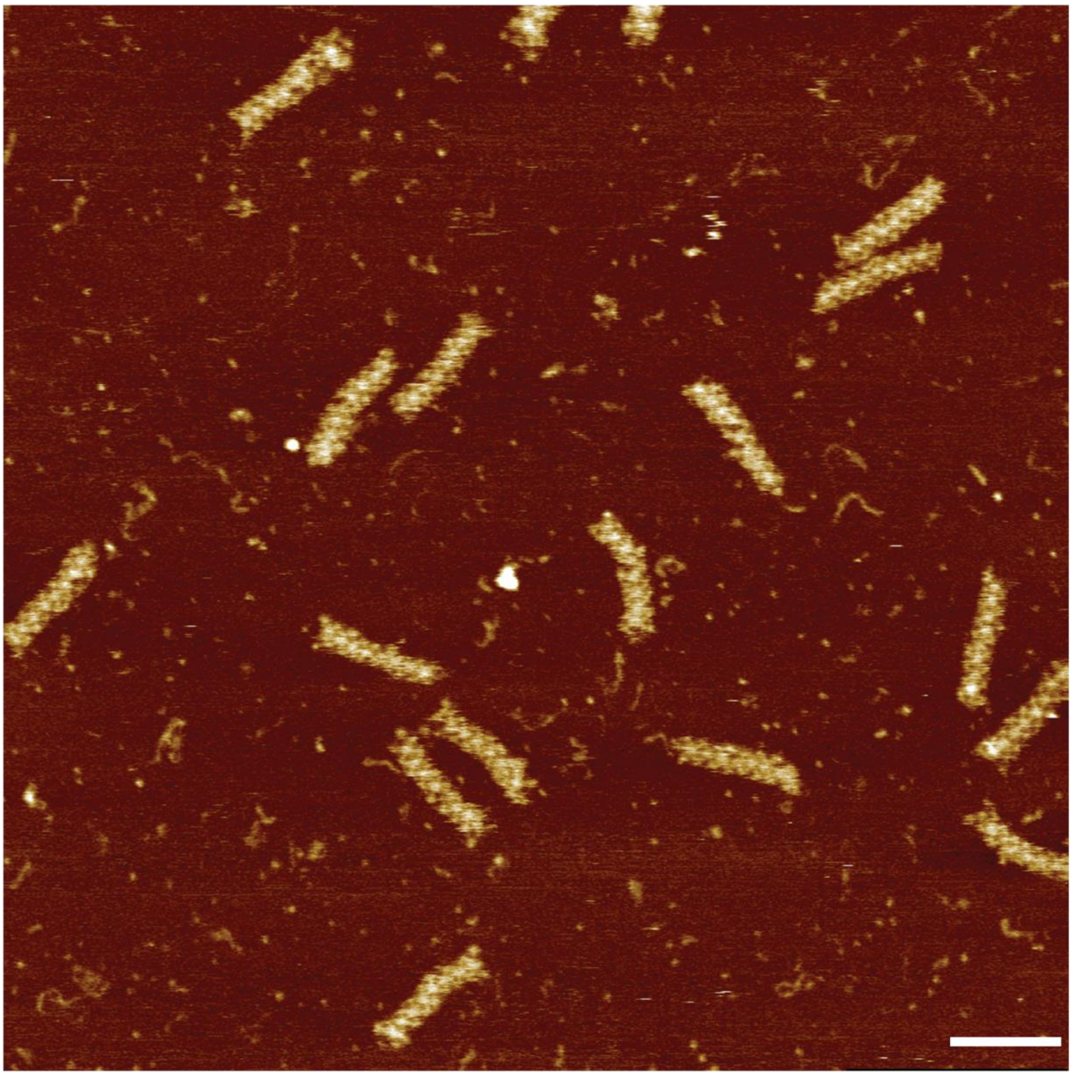

Supplementary Figure 18. Full size AFM image of straight tube (scale bar: 100 nm).

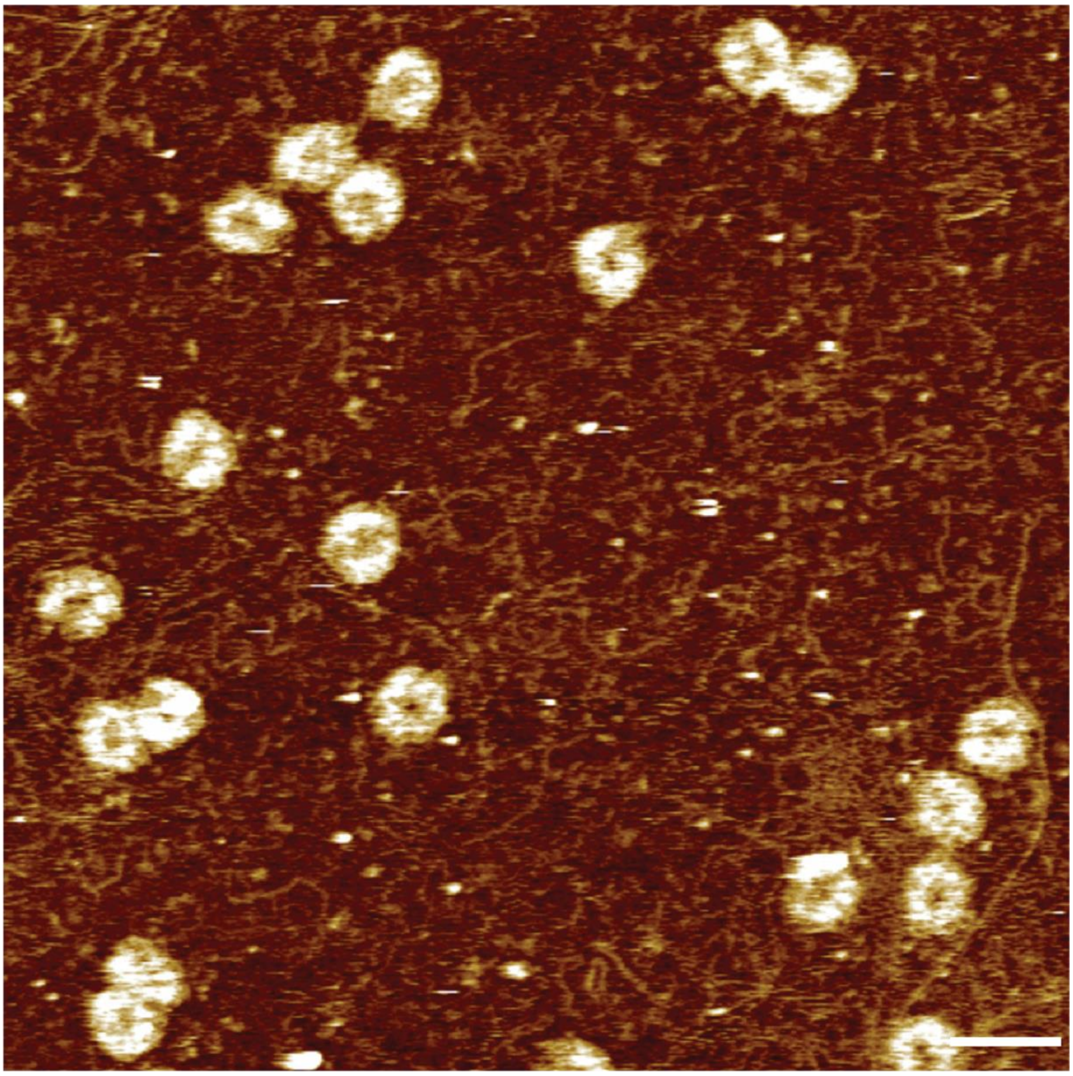

Supplementary Figure 19. Full size AFM image of donut (scale bar: 100 nm).

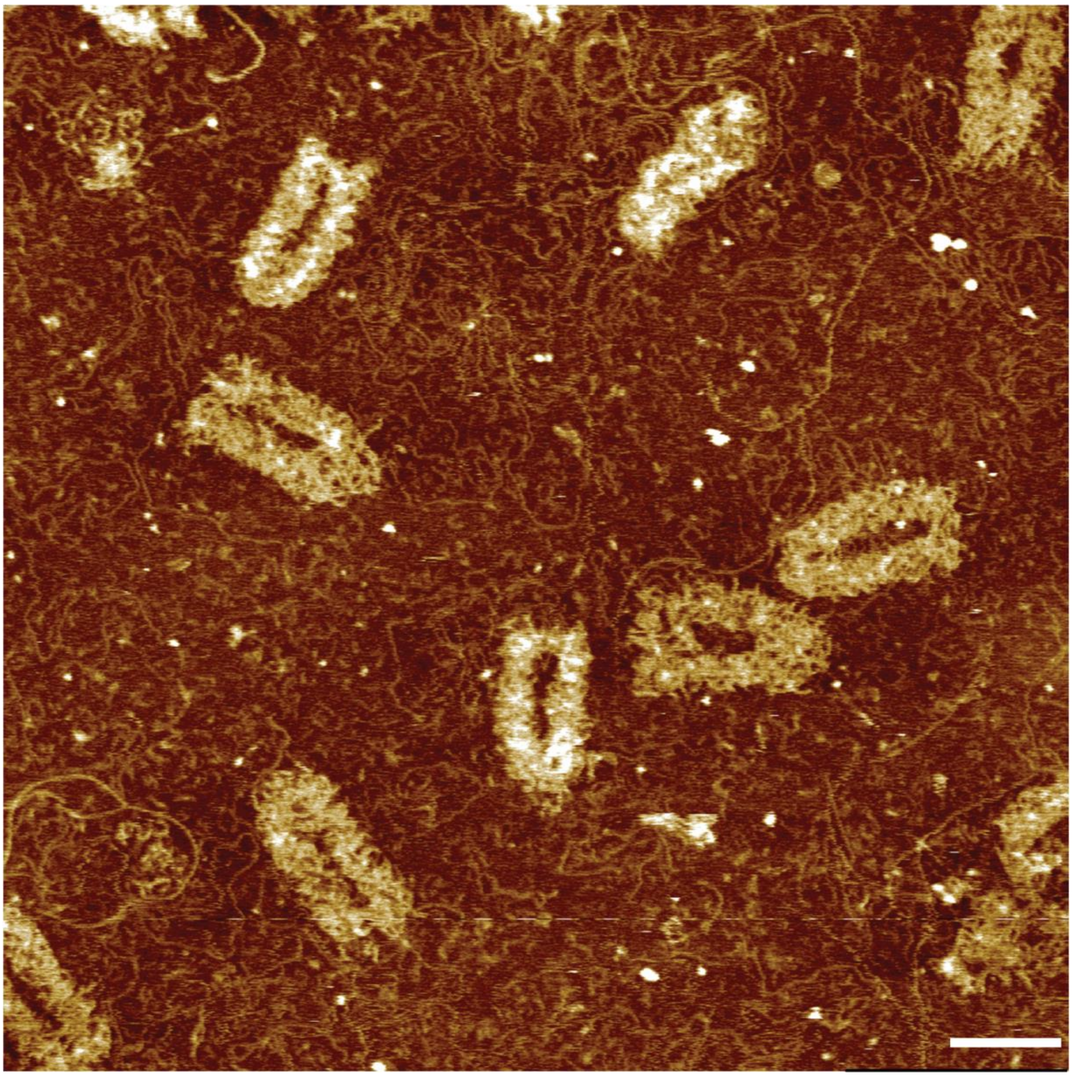

Supplementary Figure 20. Full size AFM image of U-bent tube (scale bar: 100 nm).

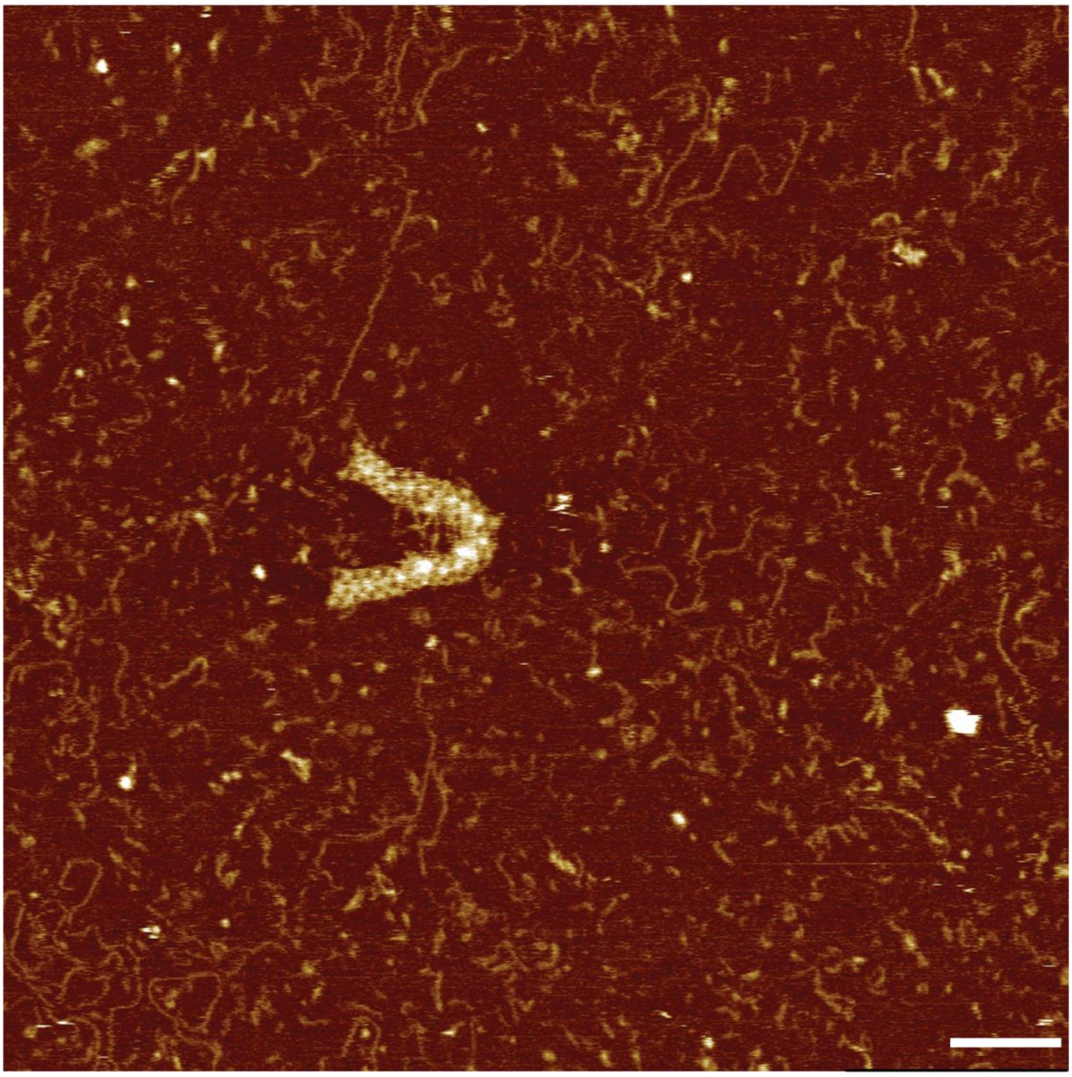

Supplementary Figure 21. Full size AFM image of 135°-bent tube (scale bar: 100 nm).

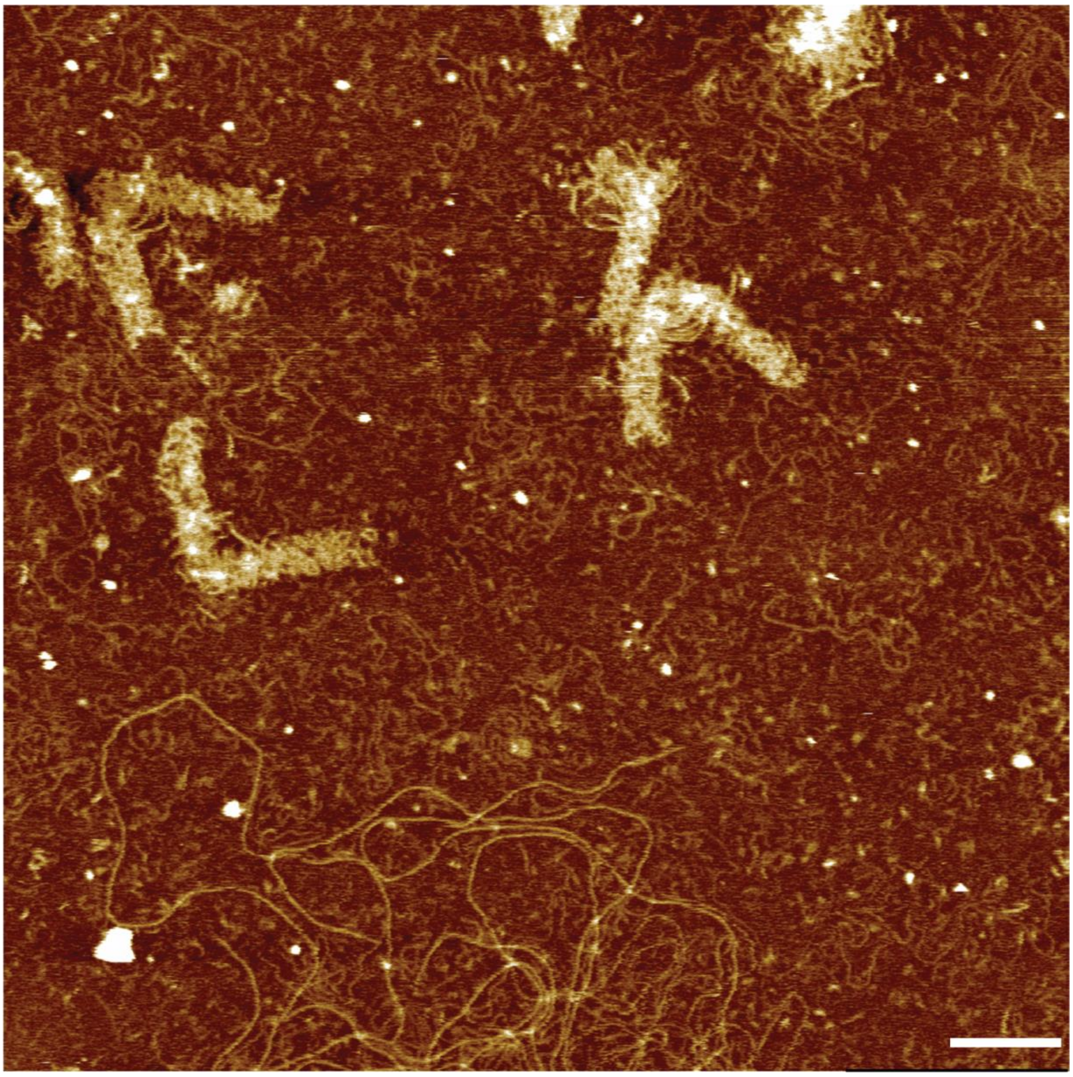

Supplementary Figure 22. Full size AFM image of 90°-bent tube (scale bar: 100 nm).

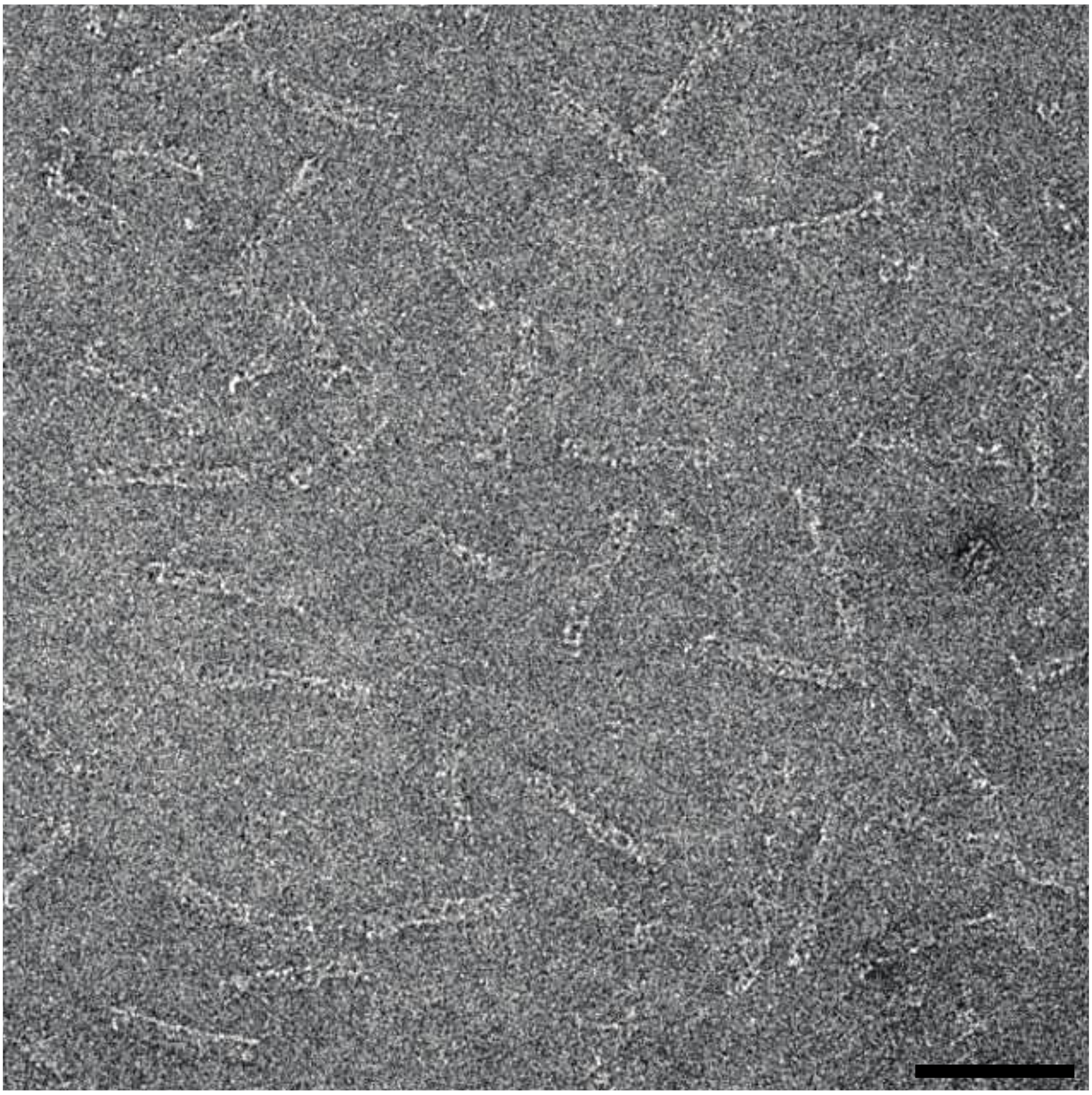

Supplementary Figure 23. Full size TEM image of straight tube (scale bar: 100 nm).

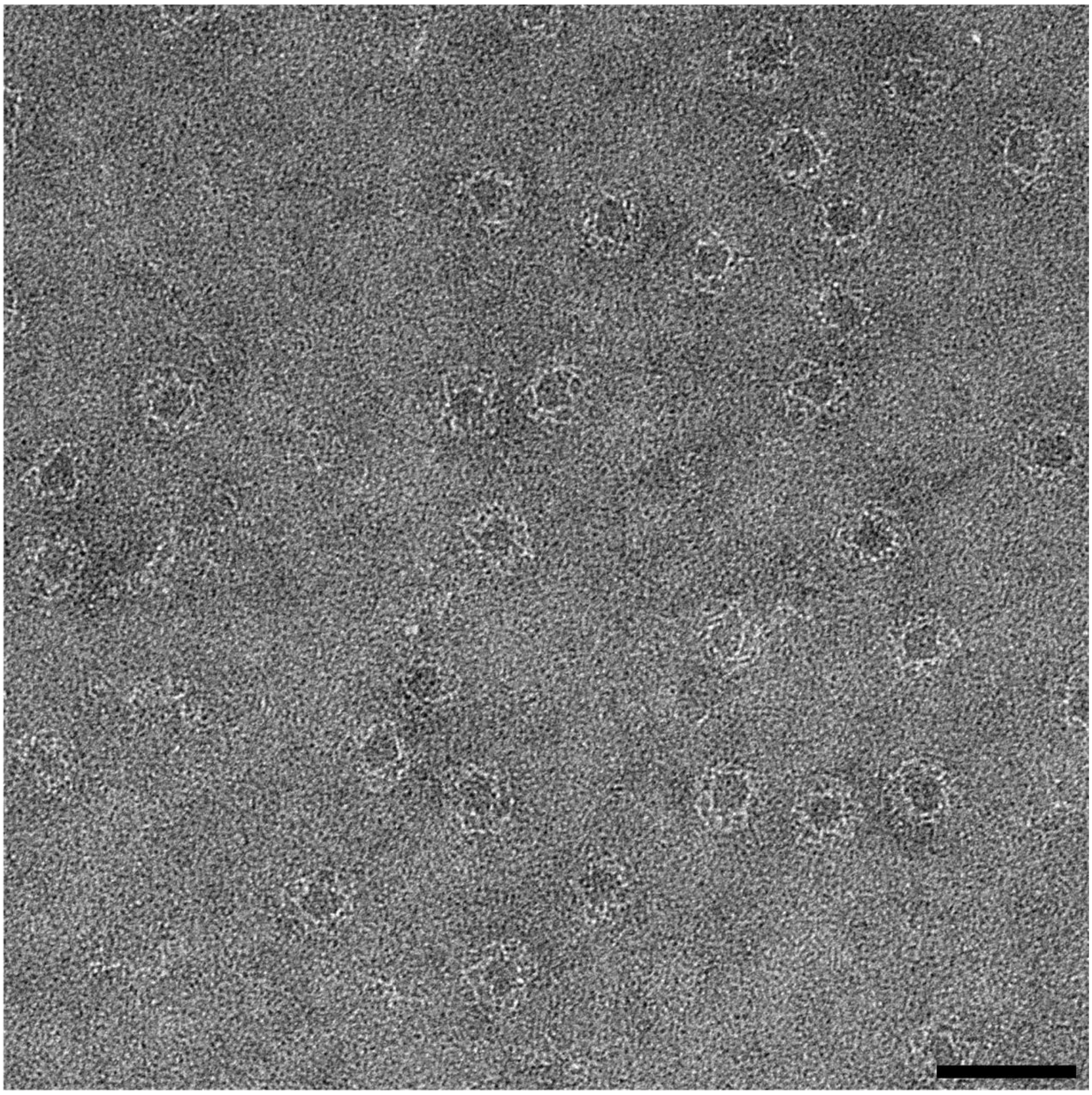

Supplementary Figure 24. Full size TEM image of donut (scale bar: 100 nm).

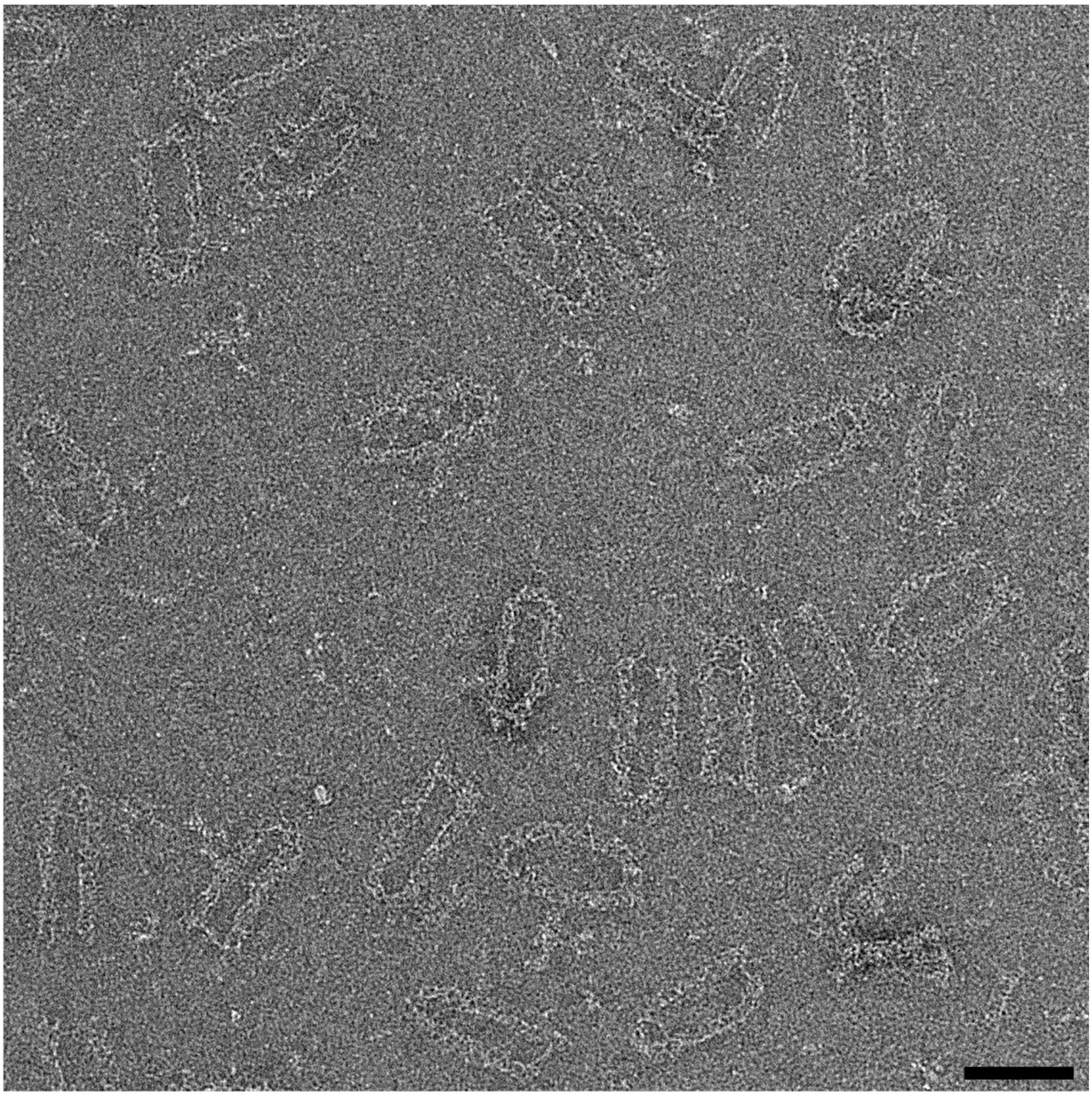

Supplementary Figure 25. Full size TEM image of U-bent tube (scale bar: 100 nm).

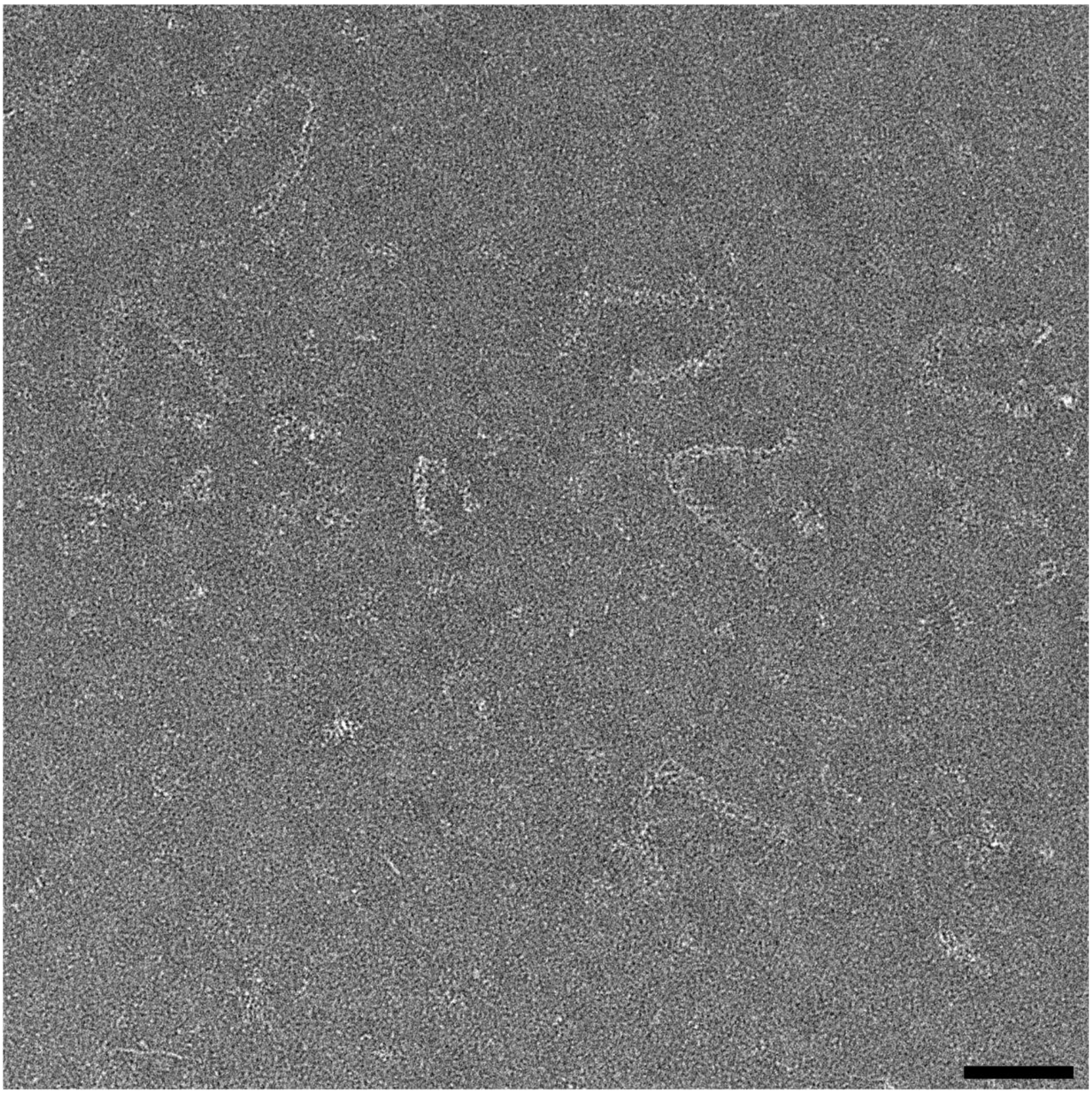

Supplementary Figure 26. Full size TEM image of 135°-bent tube (scale bar: 100 nm).

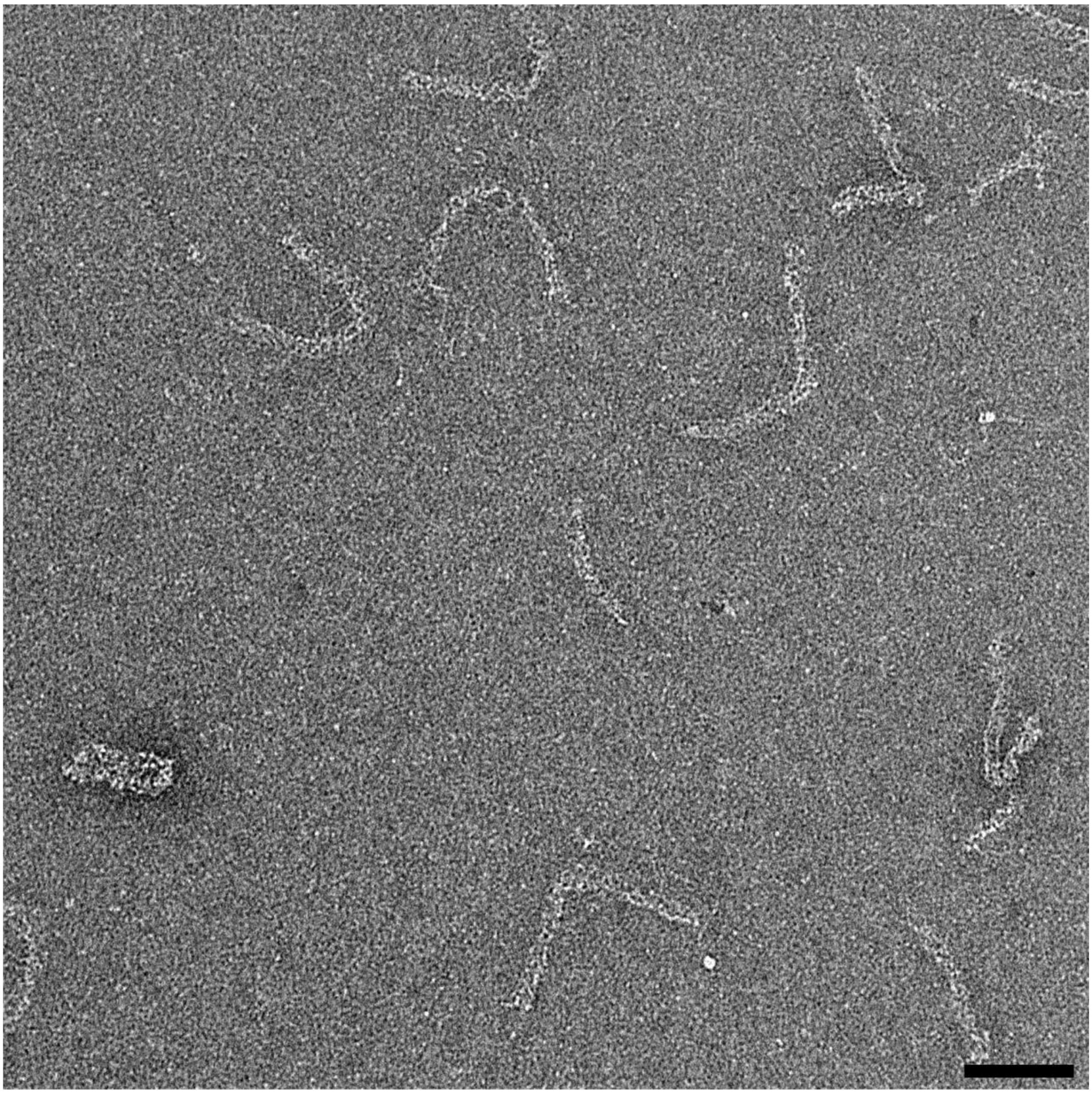

Supplementary Figure 27. Full size TEM image of 90°-bent tube (scale bar: 100 nm).

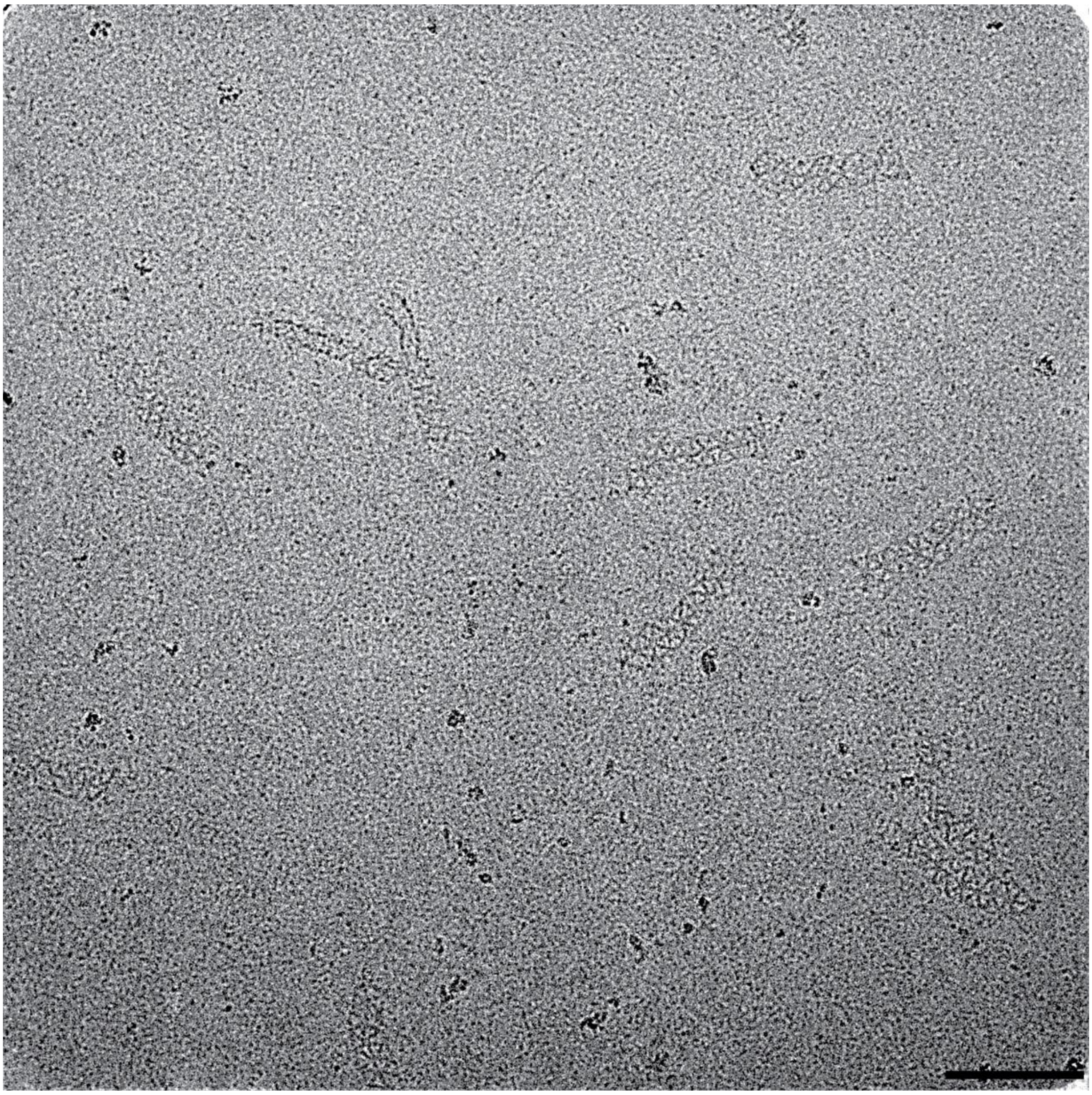

Supplementary Figure 28. Full size cryo-EM image of straight tube (scale bar: 100 nm).

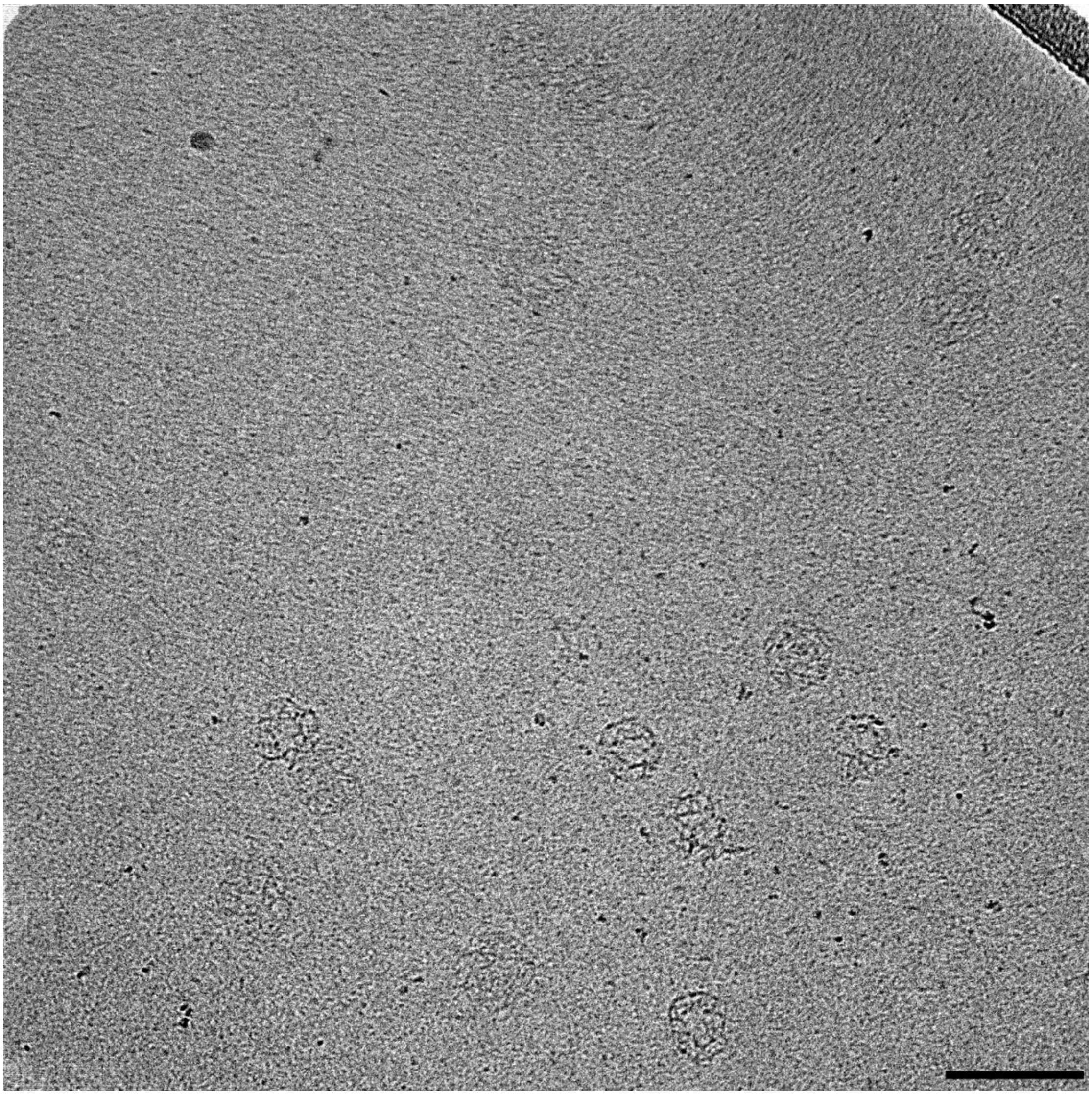

Supplementary Figure 29. Full size cryo-EM image of donut (scale bar: 100 nm).

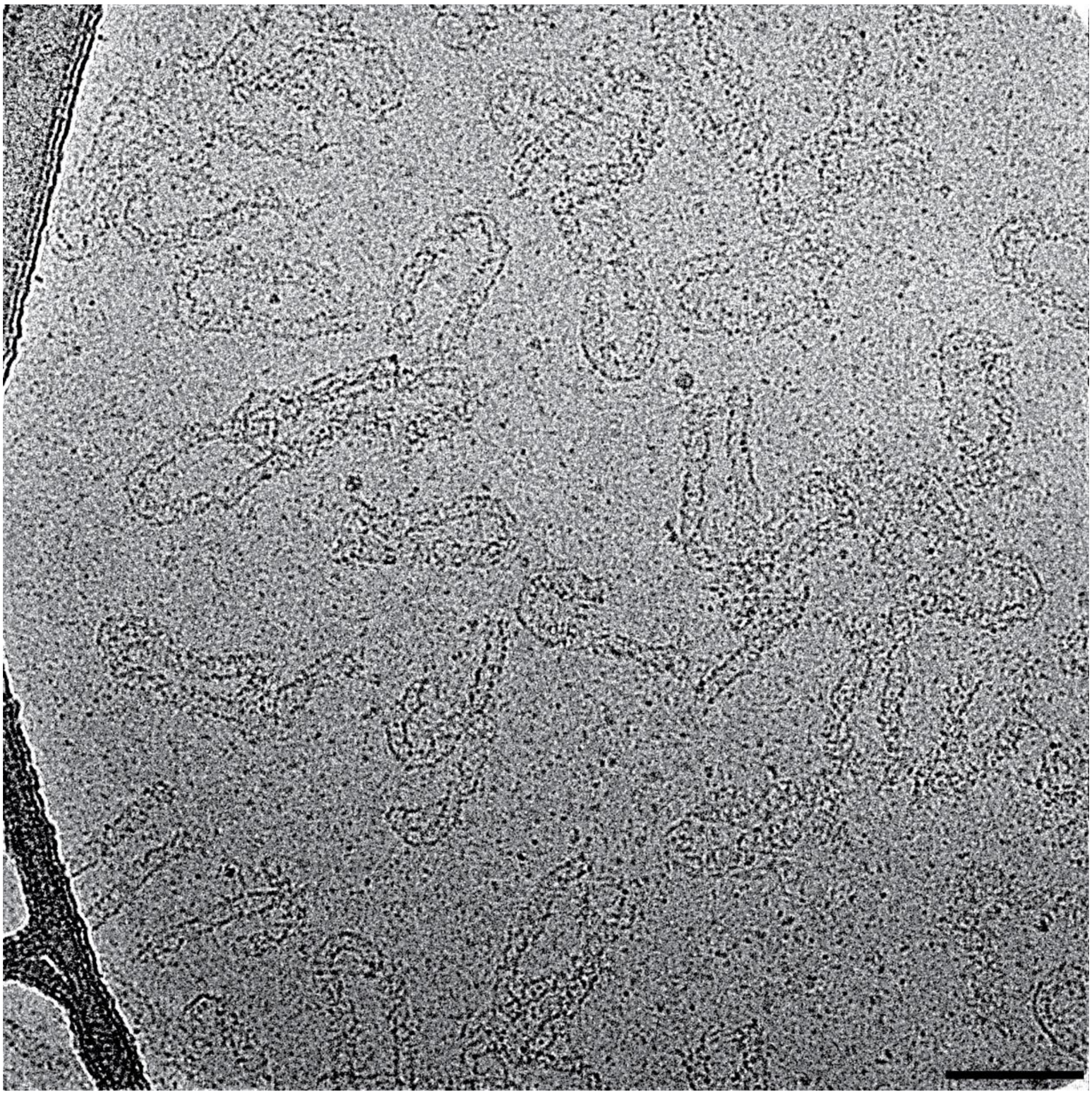

Supplementary Figure 30. Full size cryo-EM image of U-bent tube (scale bar: 100 nm).

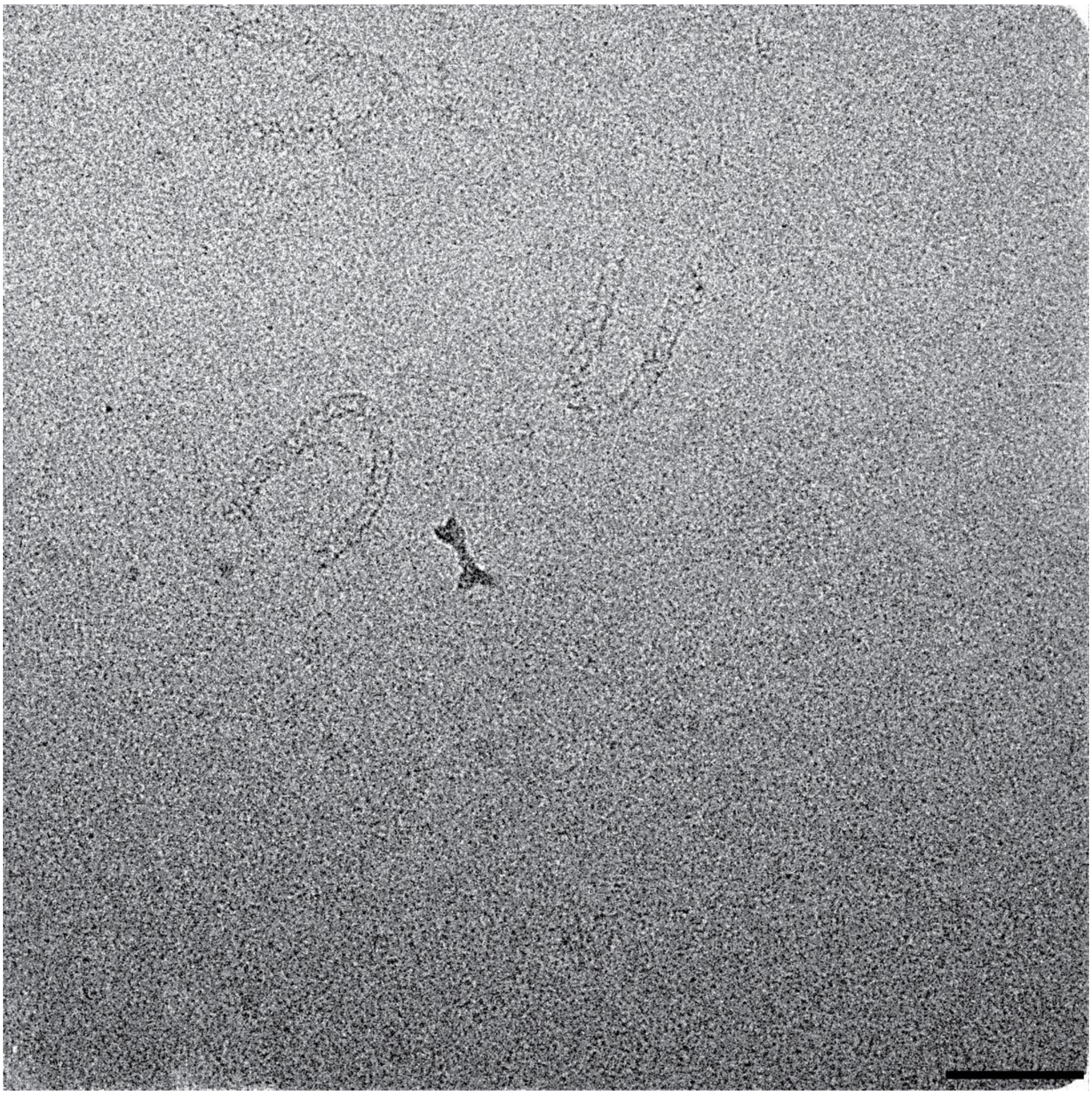

Supplementary Figure 31. Full size cryo-EM image of 135°-bent tube (scale bar: 100 nm).

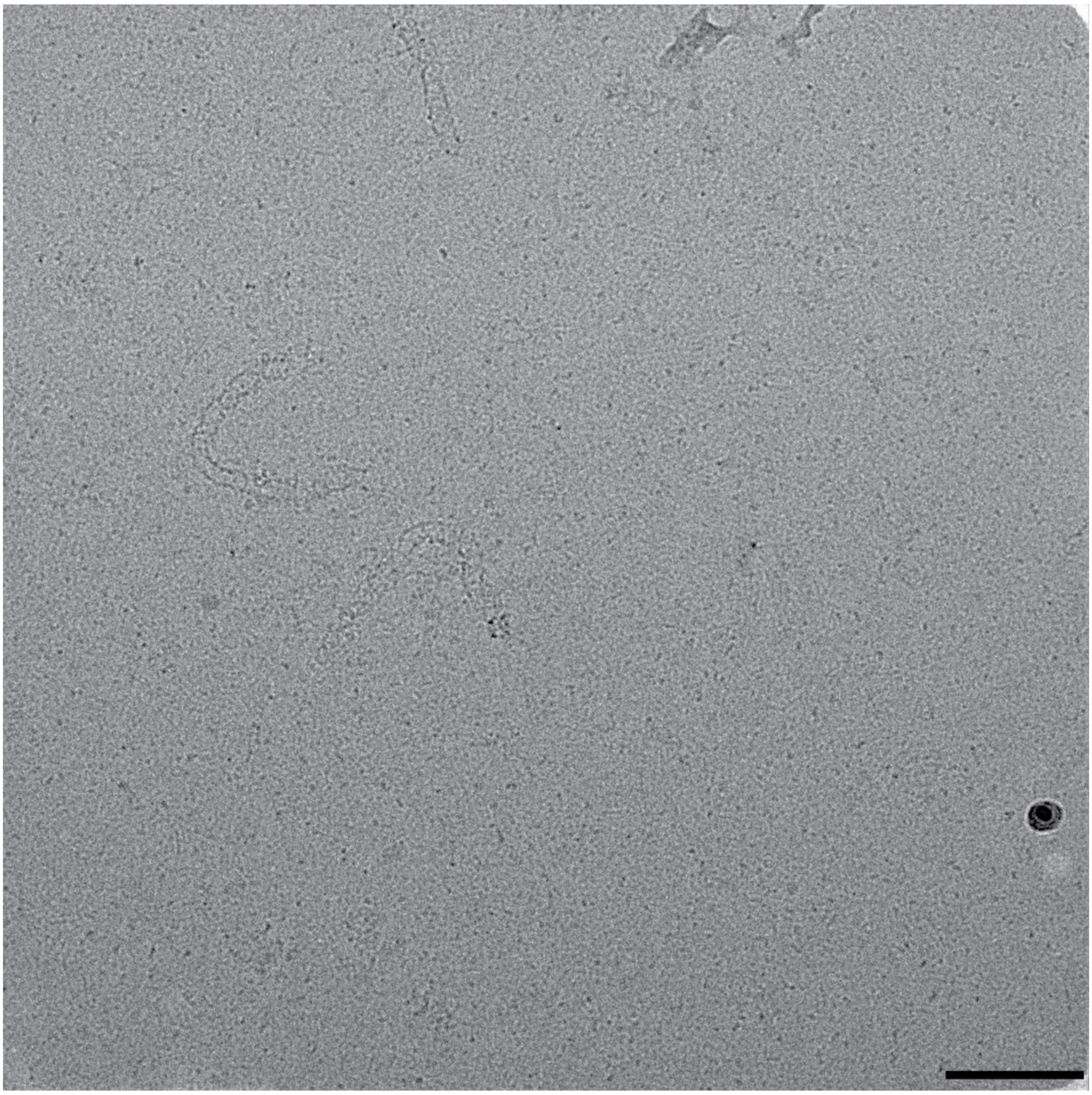

Supplementary Figure 32. Full size cryo-EM image of 90°-bent tube (scale bar: 100 nm).

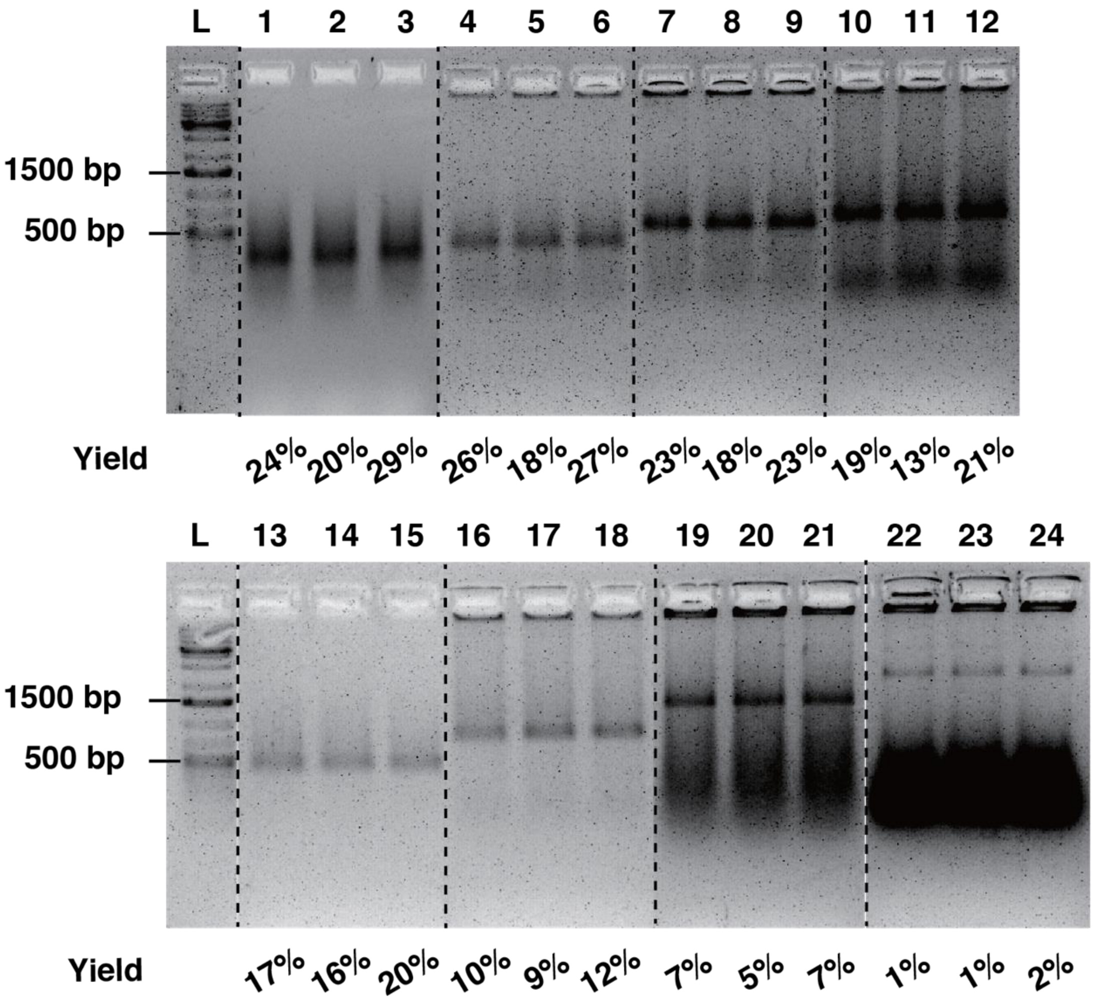

Supplementary Figure 33. Native agarose gel electrophoresis analysis for polyhedral nanostructures. Lane L: 1kb DNA ladder; lanes 1-3: three replicates of tetrahedron; lanes 4-6: three replicates of octahedron; lanes 7-9: three replicates of cuboctahedron; lanes 10-12: three replicates of icosahedron; lanes 13-15: three replicates of cube; lanes 16-18: three replicates of triangulated cube; lanes 19-21: three replicates of bucky ball; lanes 22-24: three replicates of triangulated Buckyball. Numbers at the bottom indicate assembly yields. (Results were merged from multiple gel images.)

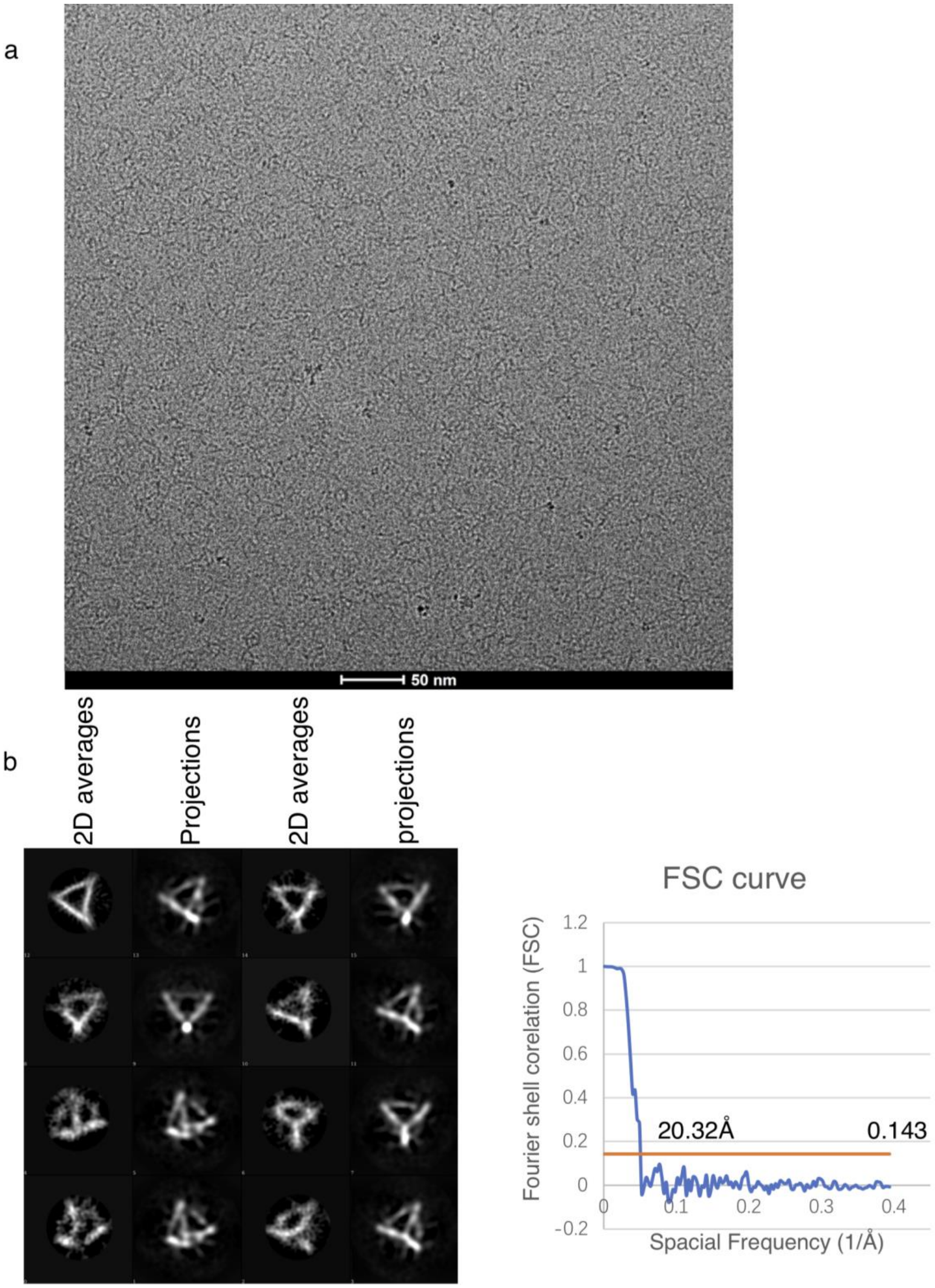

Supplementary Figure 34. Cryo-EM image and 3D reconstruction of tetrahedron. (a) A representative raw image. (b) Left: Representative 2D averages and corresponding projections from 3D reconstruction. Right: Gold-standard FSC plot of the 3D reconstruction.

a

b

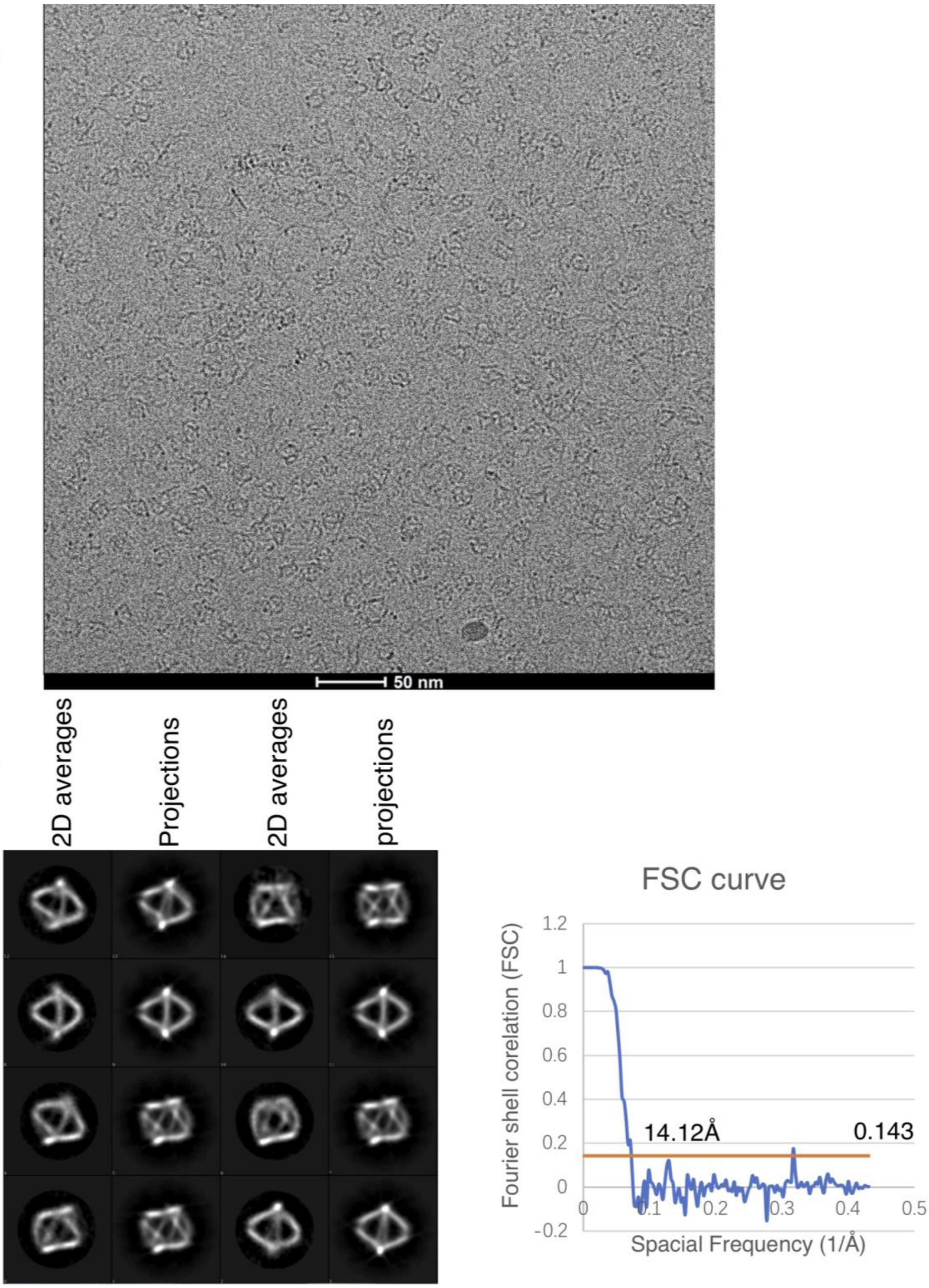

Supplementary Figure 35. Cryo-EM image and 3D reconstruction of octahedron. (a) A representative raw image. (b) Left: Representative 2D averages and corresponding projections from 3D reconstruction. Right: Gold-standard FSC plot of the 3D reconstruction.

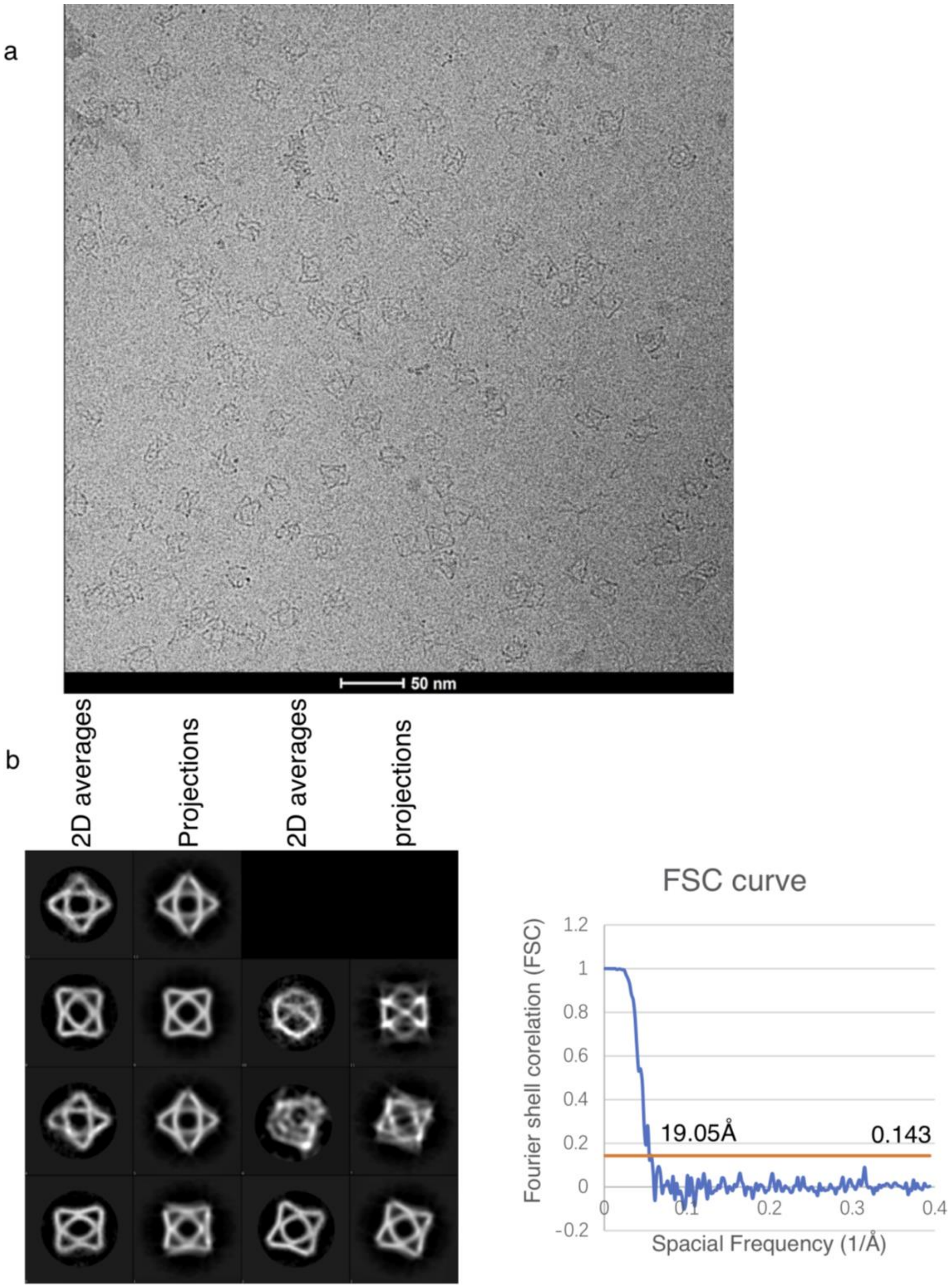

Supplementary Figure 36. Cryo-EM image and 3D reconstruction of cuboctahedron. (a) A representative raw image. (b) Left: Representative 2D averages and corresponding projections from 3D reconstruction. Right: Gold-standard FSC plot of the 3D reconstruction.

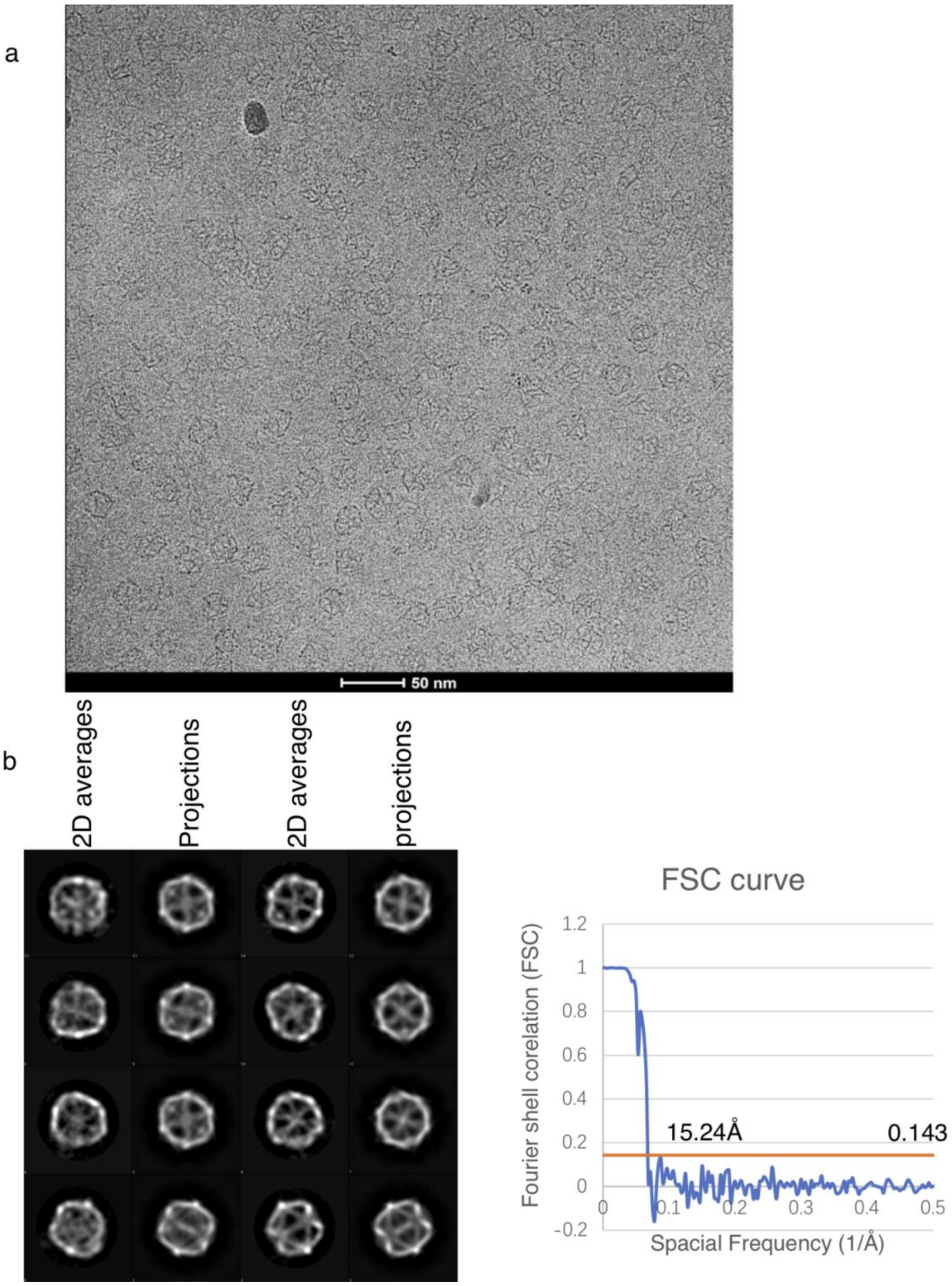

Supplementary Figure 37. Cryo-EM image and 3D reconstruction of icosahedron. (a) A representative raw image. (b) Left: Representative 2D averages and corresponding projections from 3D reconstruction. Right: Gold-standard FSC plot of the 3D reconstruction.

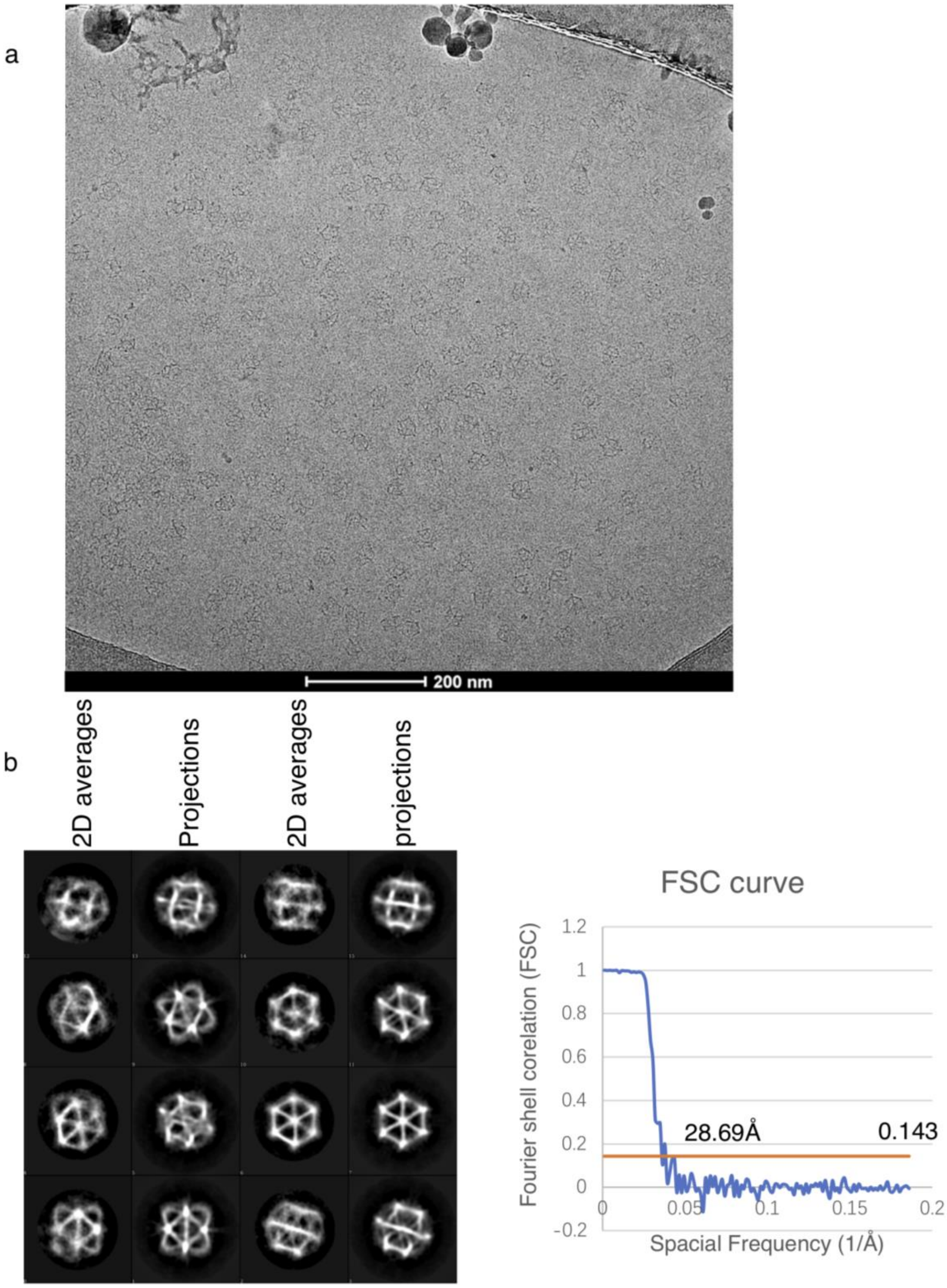

Supplementary Figure 38. Cryo-EM image and 3D reconstruction of triangulated cube. (a) A representative raw image. (b) Left: Representative 2D averages and corresponding projections from 3D reconstruction. Right: Gold-standard FSC plot of the 3D reconstruction.

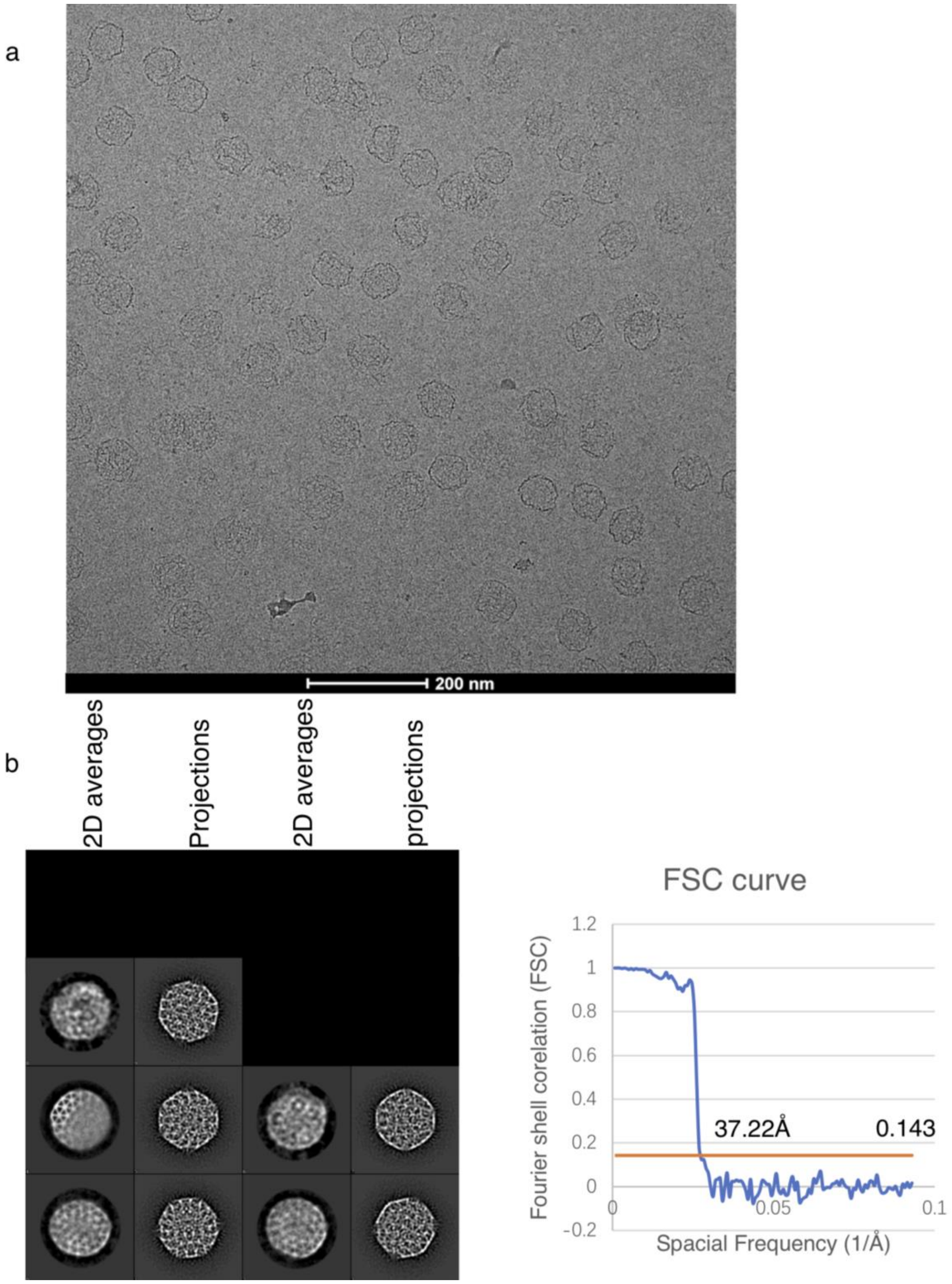

Supplementary Figure 39. Cryo-EM image and 3D reconstruction of triangulated Buckyball. (a) A representative raw image. (b) Left: Representative 2D averages and corresponding projections from 3D reconstruction. Right: Gold-standard FSC plot of the 3D reconstruction.

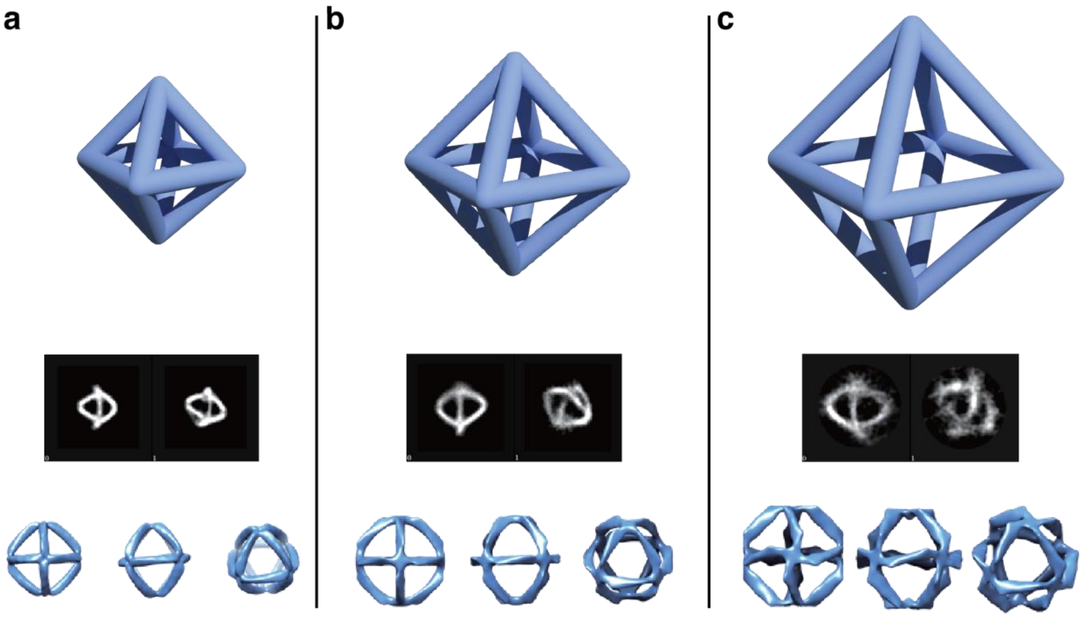

Supplementary Figure 40. Designs and cryo-EM images of octahedra with 32-bp edges (a), 42-bp edges (b) and 52-bp edges (c). Panels from top to bottom: models of octahedra, examples of 2D classification results and 3D maps.

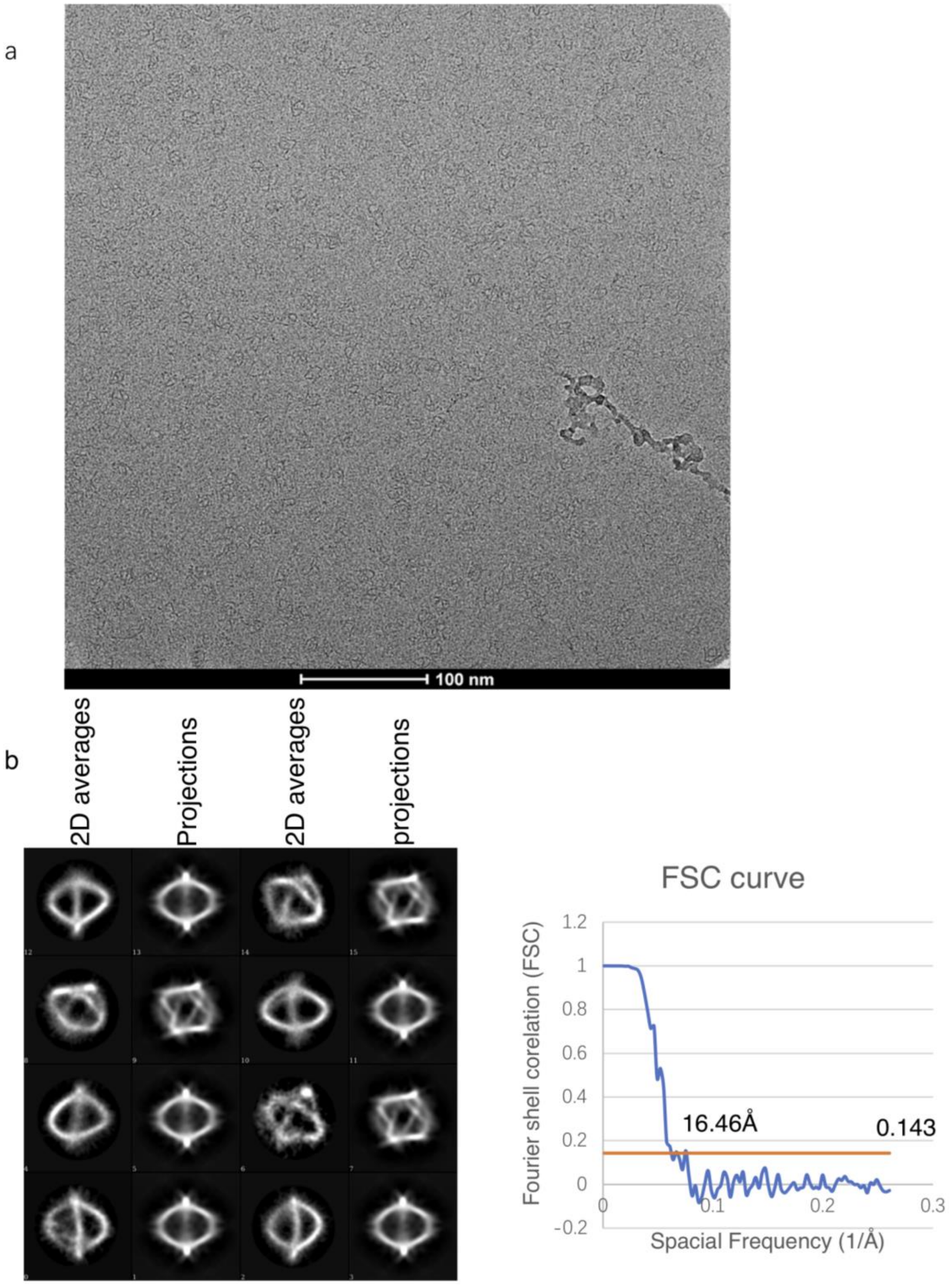

Supplementary Figure 41. Cryo-EM image and 3D reconstruction of octahedron with 42-bp edges. (a) A representative raw image. (b) Left: Representative 2D averages and corresponding projections from 3D reconstruction. Right: Gold-standard FSC plot of the 3D reconstruction.

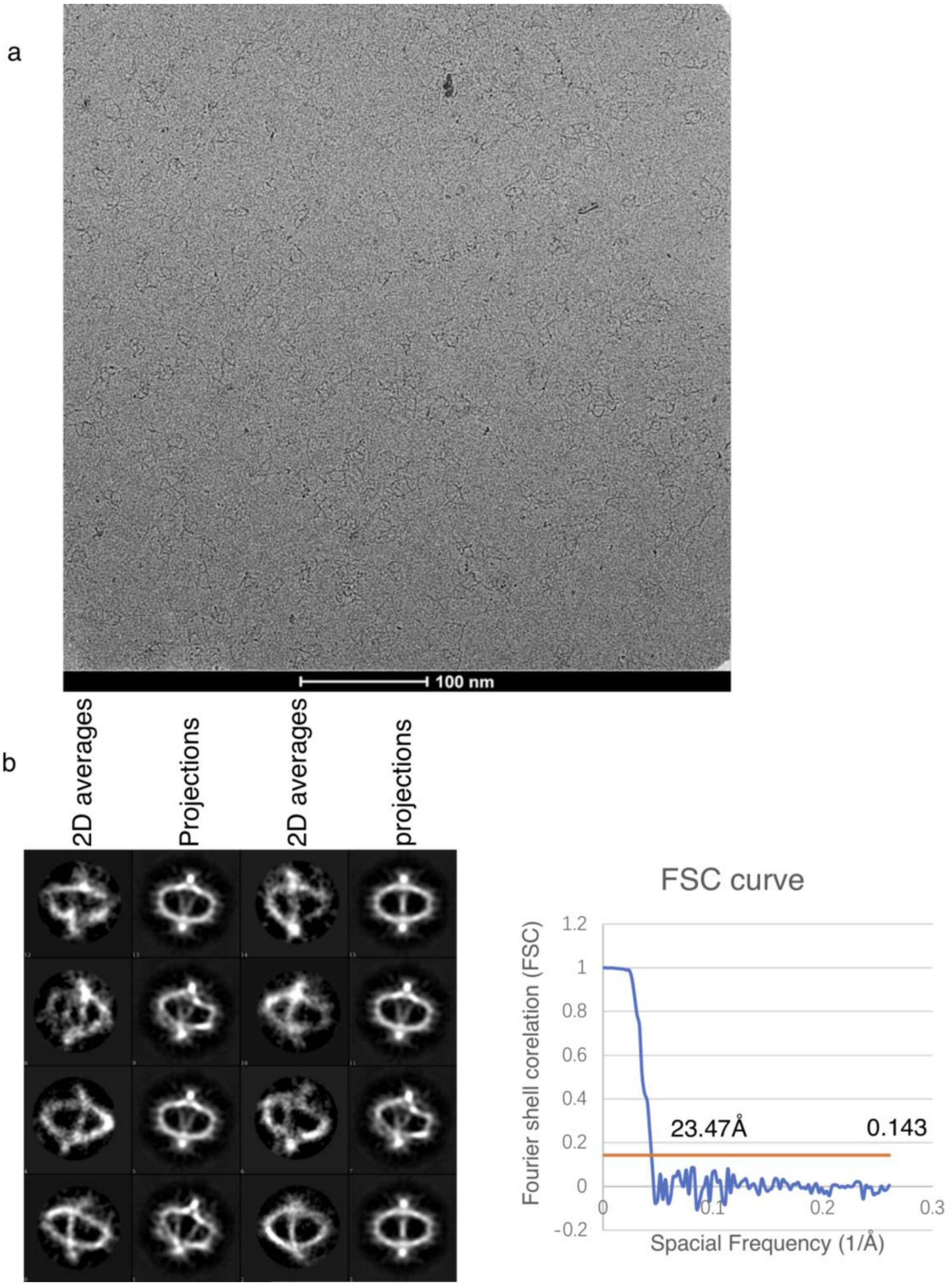

Supplementary Figure 42. Cryo-EM image and 3D reconstruction of octahedron with 52-bp edges. (a) A representative raw image. (b) Left: Representative 2D averages and corresponding projections from 3D reconstruction. Right: Gold-standard FSC plot of the 3D reconstruction.

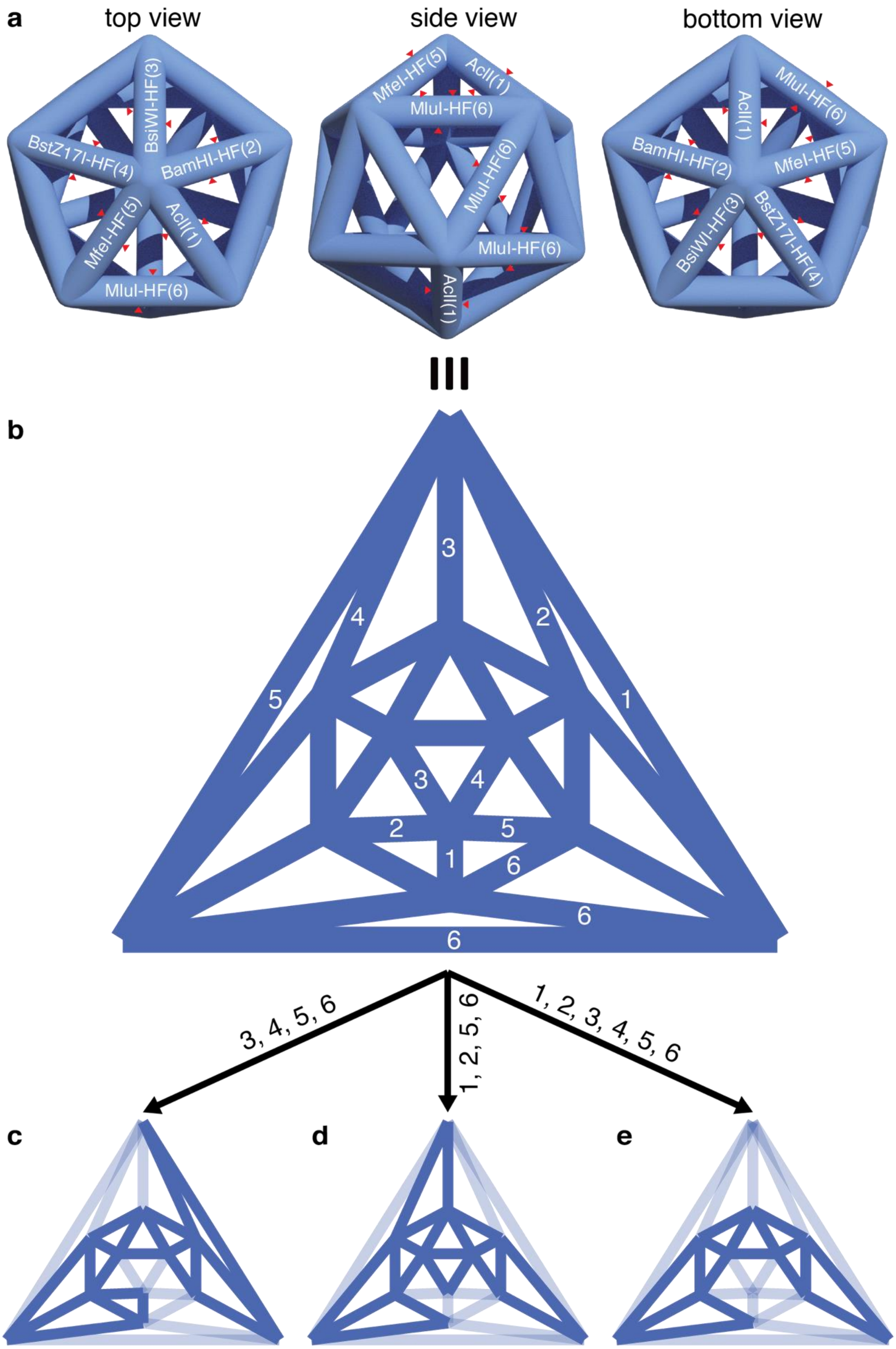

Supplementary Figure 43. Design of enzyme cleavage sites on an icosahedron. (a) Different views (from left to right: top view, side view and bottom view) of an icosahedron with restriction sites. All edges are composed of 42-bp double helices. In the middle of some edges of the icosahedron, different enzyme sites (*Acl*I, *Bam*HI-HF, *Bsi*WI-HF, *Bst*Z17I-HF, *Mfe*I-HF, *Mlu*I-HF marked in white) are designed. The restriction sites of the corresponding enzymes are

indicated by red arrowheads in the diagram. (b) Schlegel diagram of an icosahedron. Cleavage sites for different kinds of restriction enzymes are indicated by numbers on corresponding edges. (c-e) Cleavage patterns after treatments of specific cocktails of the chosen restriction enzyme collection. (c) Resulting structure pattern I with cleavage sites for *Bsi*WI-HF, *Bst*Z17I-HF, *Mfe*I-HF and *Mlu*I-HF. (d) Resulting structure pattern II with cleavage sites for *Acl*I, *Bam*HI-HF, *Mfe*I-HF, *Mlu*I-HF. (e) Resulting structure pattern III with cleavage sites for *Acl*I, *Bam*HI-HF, *Bsi*WI-HF, *Bst*Z17I-HF, *Mfe*I-HF, *Mlu*I-HF.

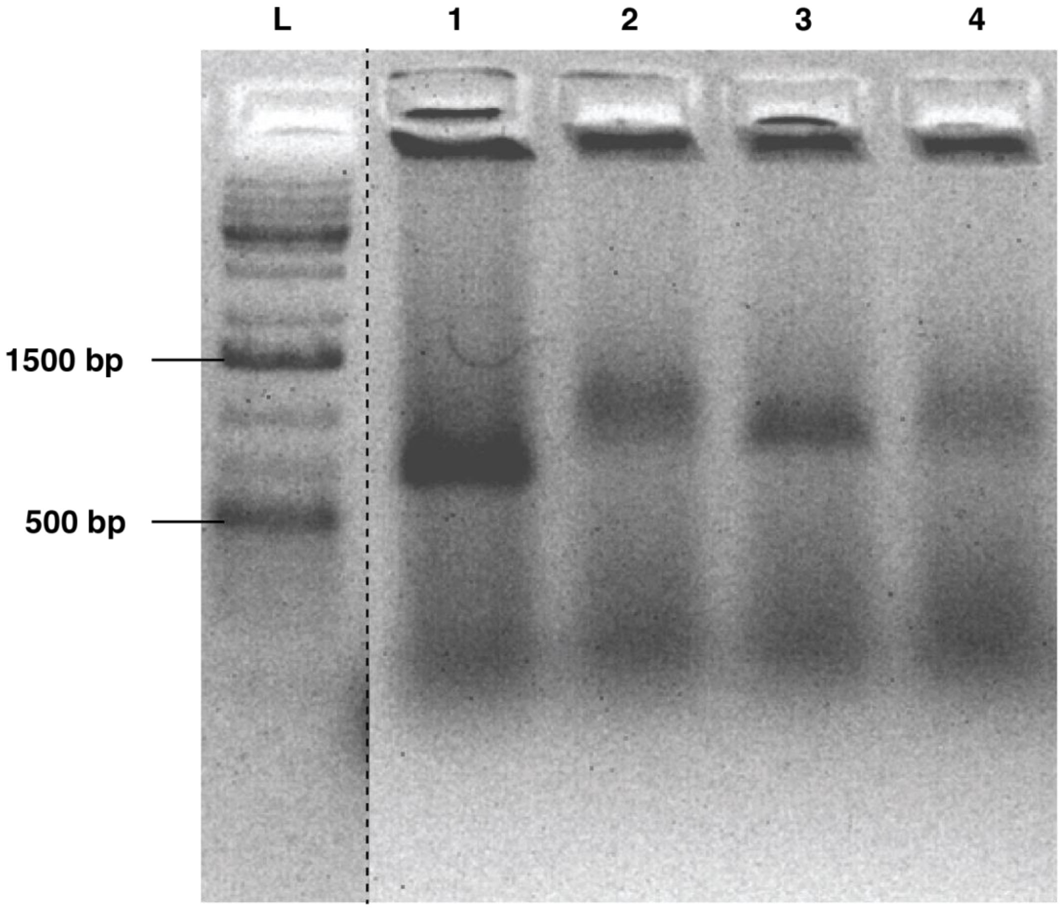

Supplementary Figure 44. Native agarose gel electrophoresis analysis for enzyme cleavage on an icosahedron with restriction sites. Lane L: 1kb DNA ladder; lane 1: icosahedron with restriction sites without cleavage; lane 2: pattern I resulted from cleavage by enzyme cocktail I; lane 3: pattern II resulted from cleavage by enzyme cocktail II; lane 4: pattern III resulted from cleavage by enzyme cocktail III.

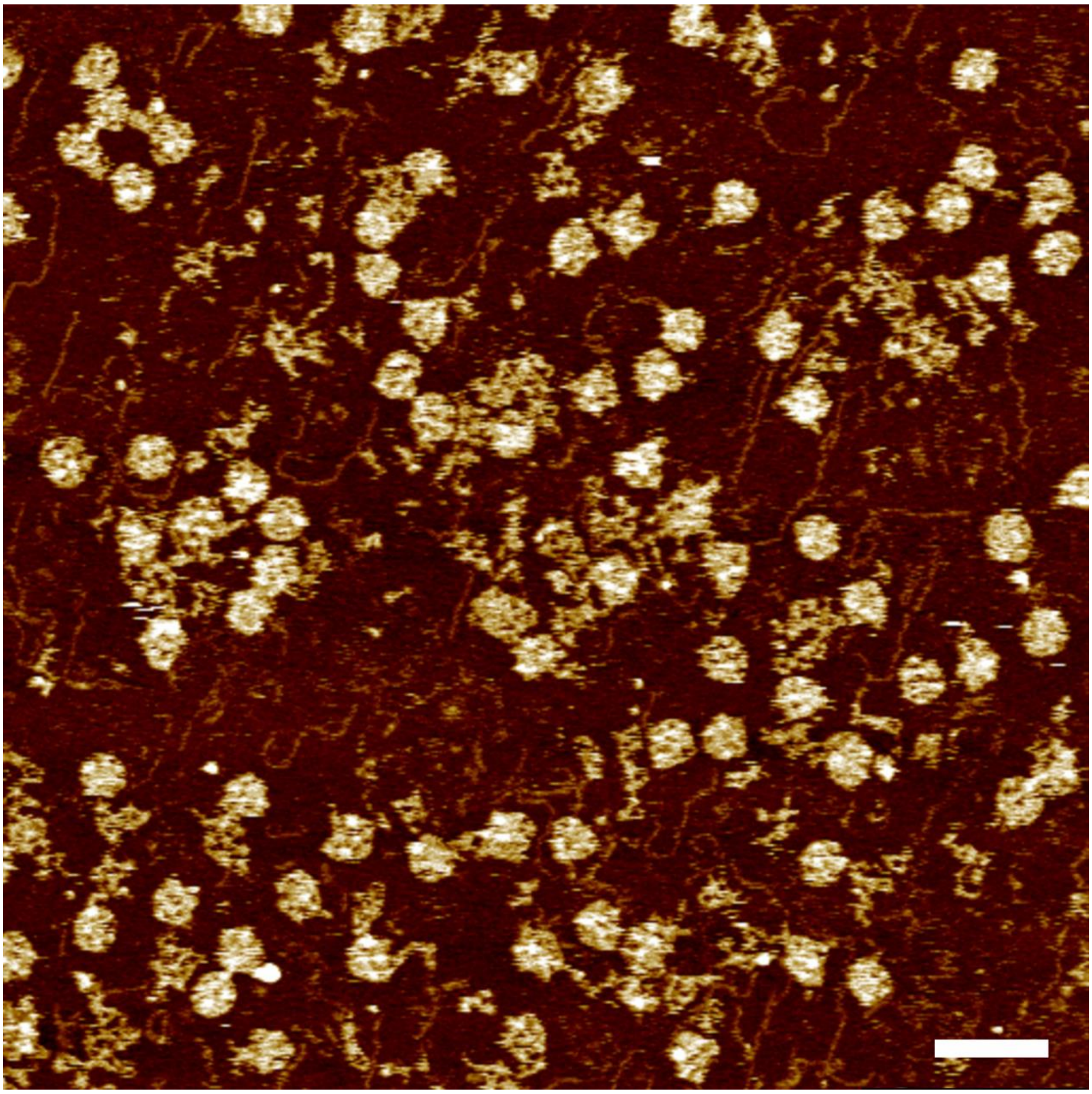

Supplementary Figure 45. Full size AFM image of icosahedron with restriction sites without cleavage (scale bar: 100 nm).

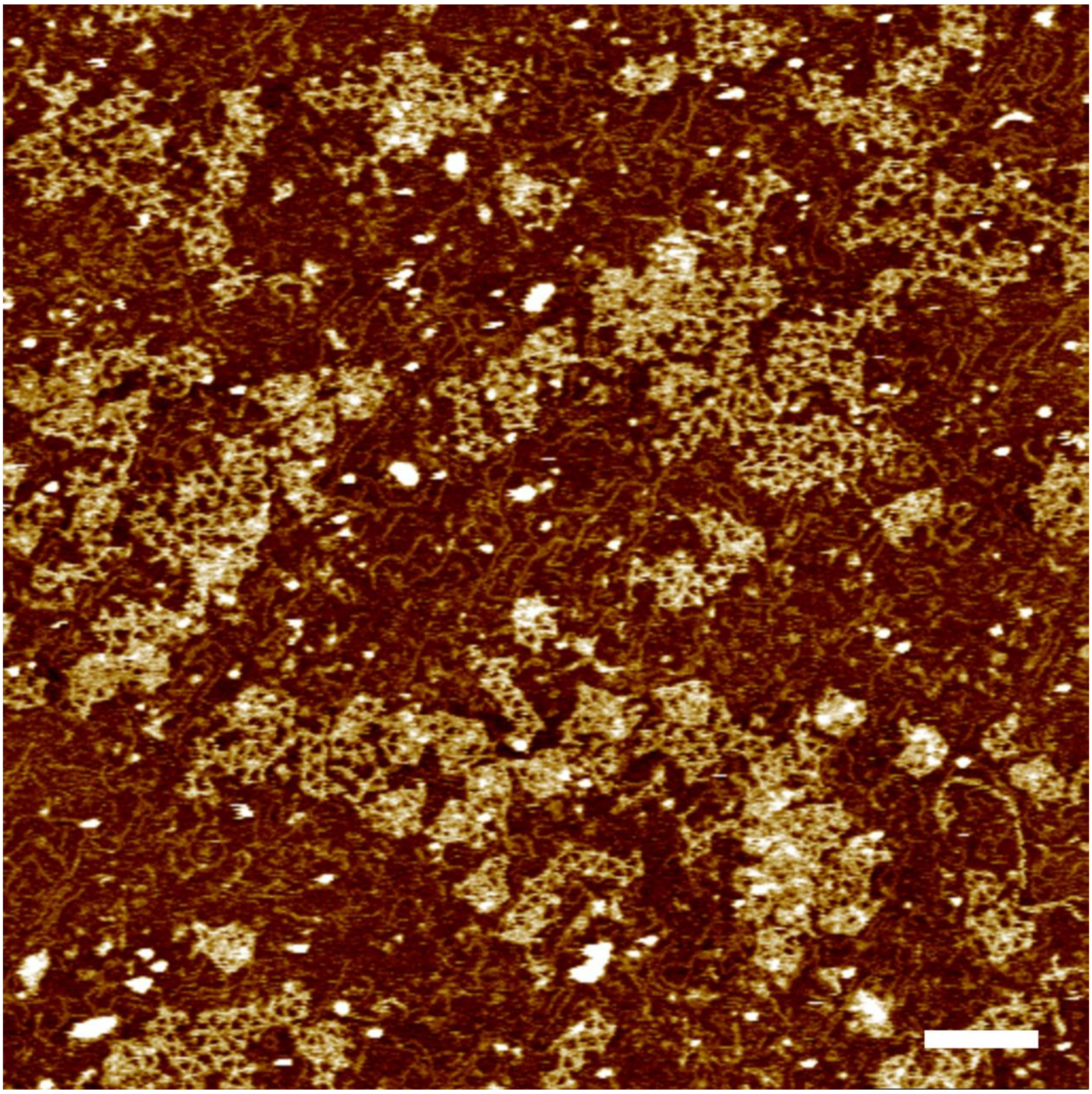

Supplementary Figure 46. Full size AFM image of pattern I resulted from cleavage by enzyme cocktail I (scale bar: 100 nm).

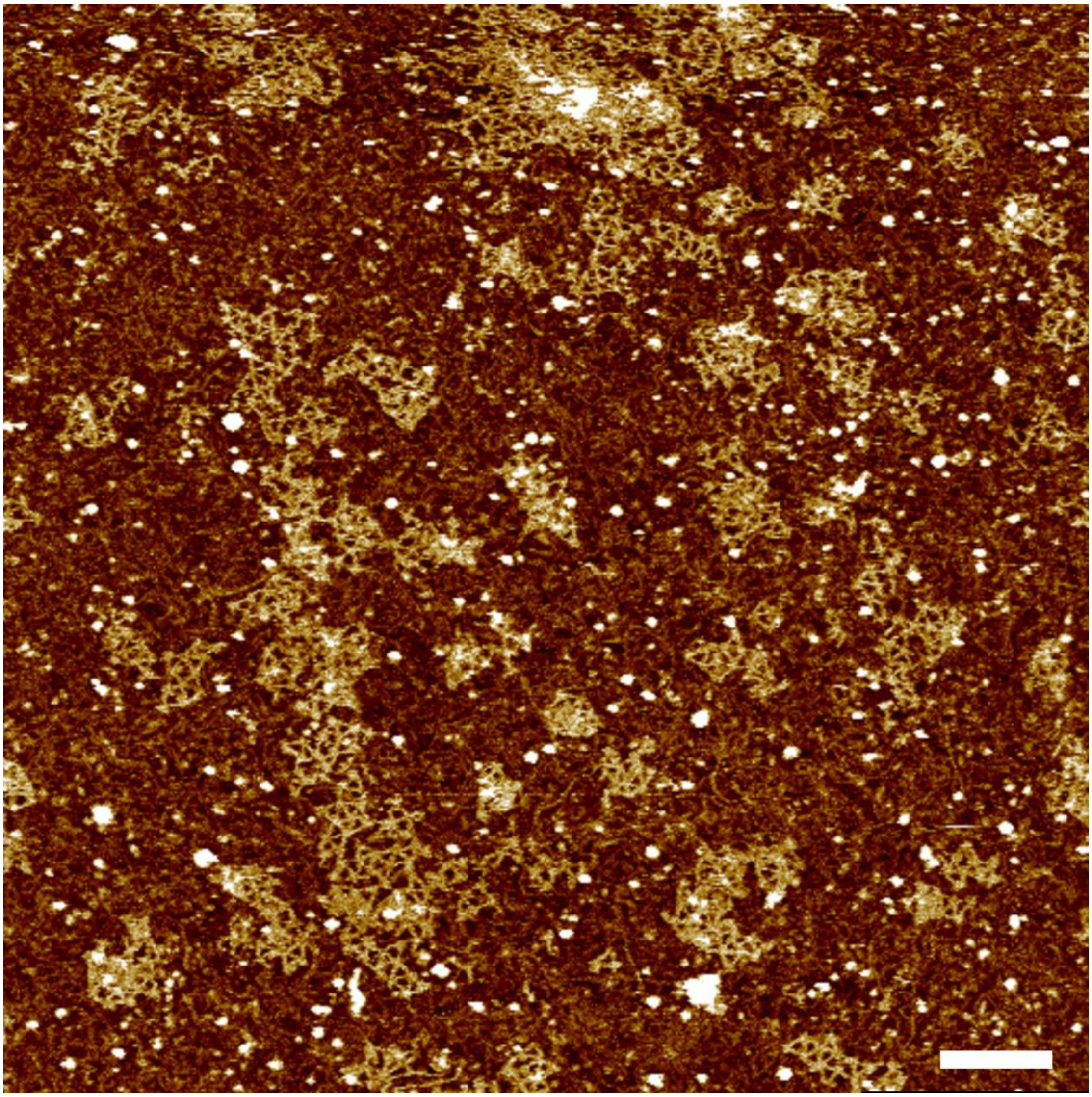

Supplementary Figure 47. Full size AFM image of pattern II resulted from cleavage by enzyme cocktail II (scale bar: 100 nm).

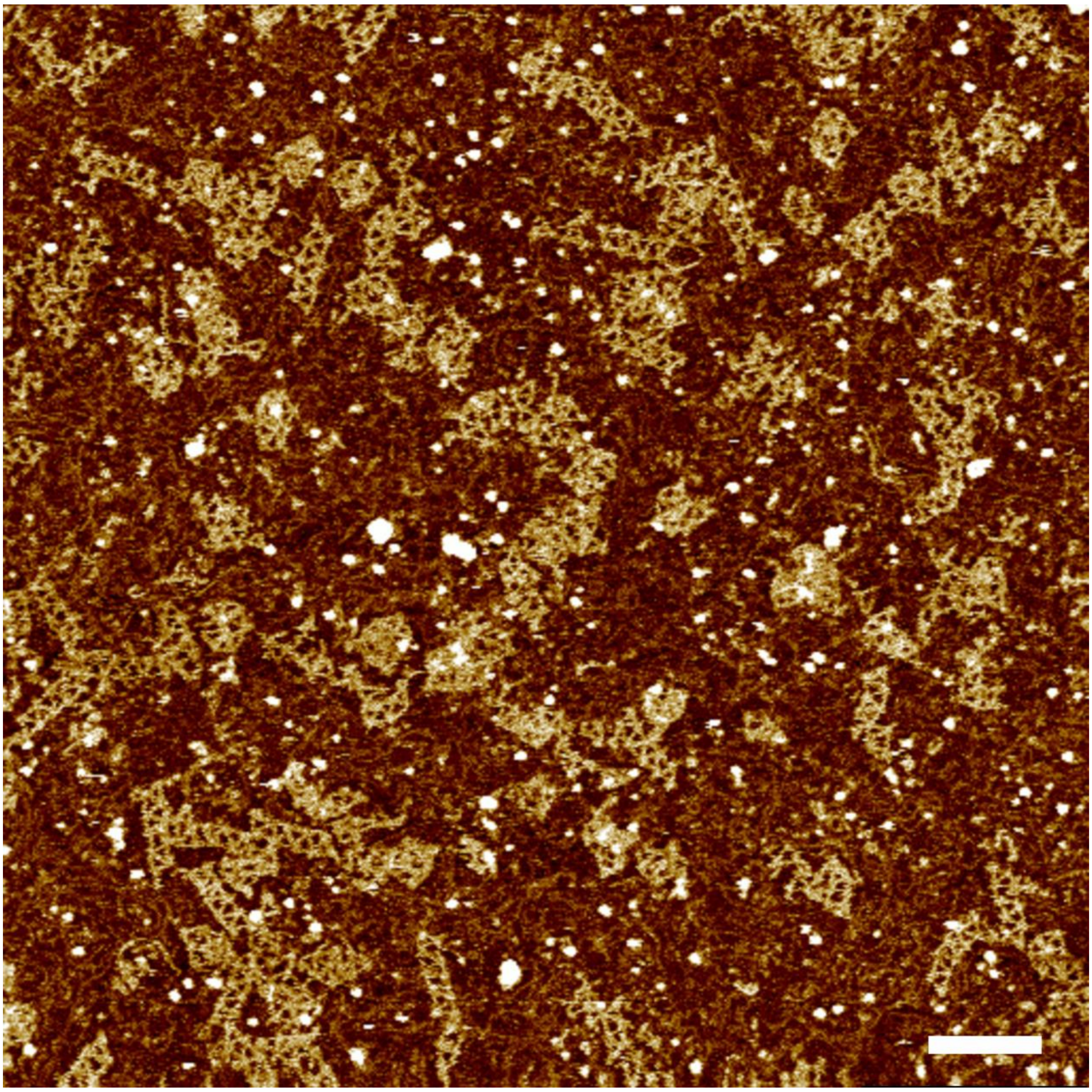

Supplementary Figure 48. Full size AFM image of pattern III resulted from cleavage by enzyme cocktail III (scale bar: 100 nm).

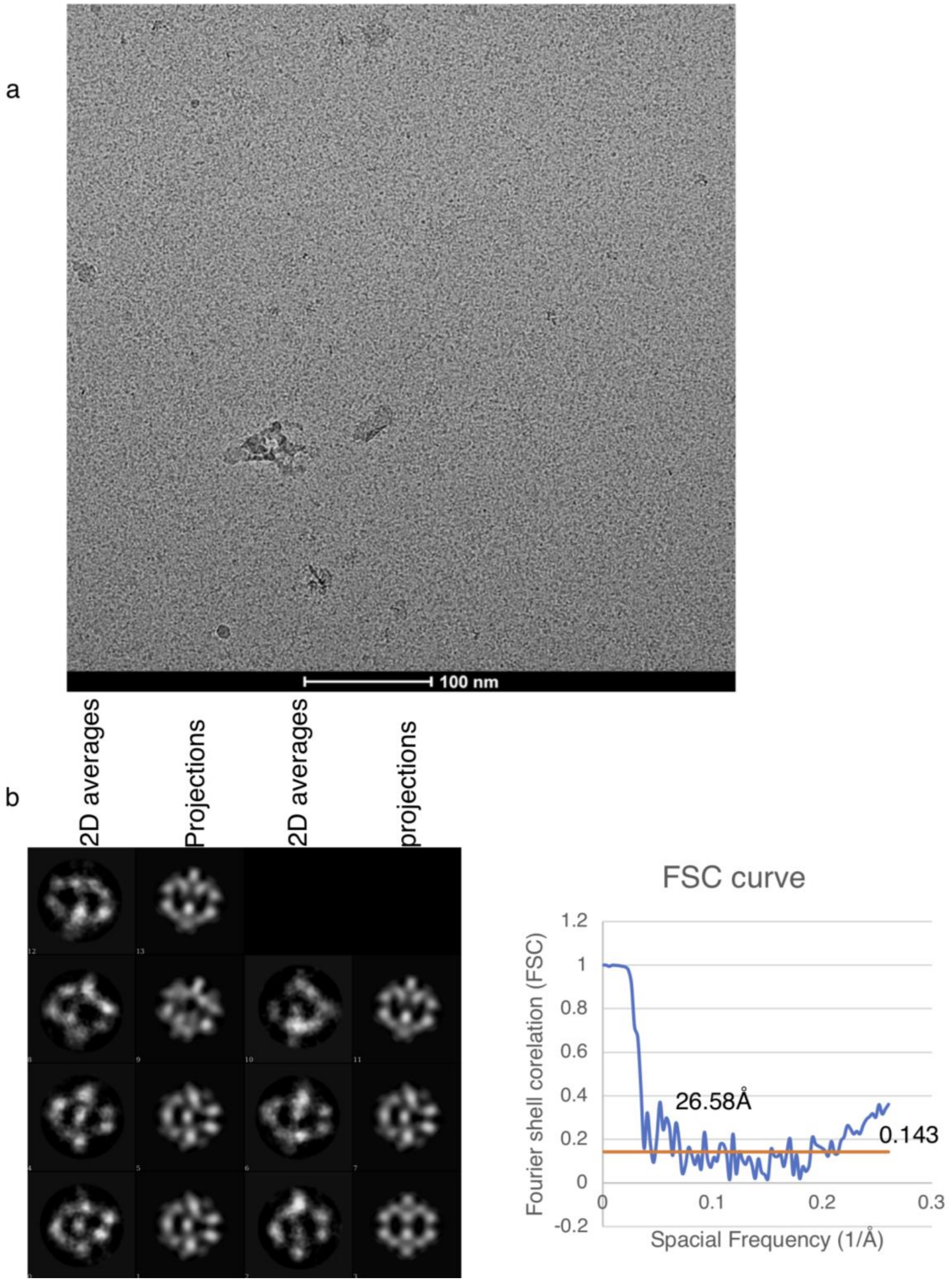

Supplementary Figure 49. Cryo-EM image and 3D reconstruction of Octahedron-MBP tagged streptavidin monomer complex. (a) A representative raw image. (b) Left: Representative 2D averages and corresponding projections from 3D reconstruction. Right: Gold-standard FSC plot of the 3D reconstruction.

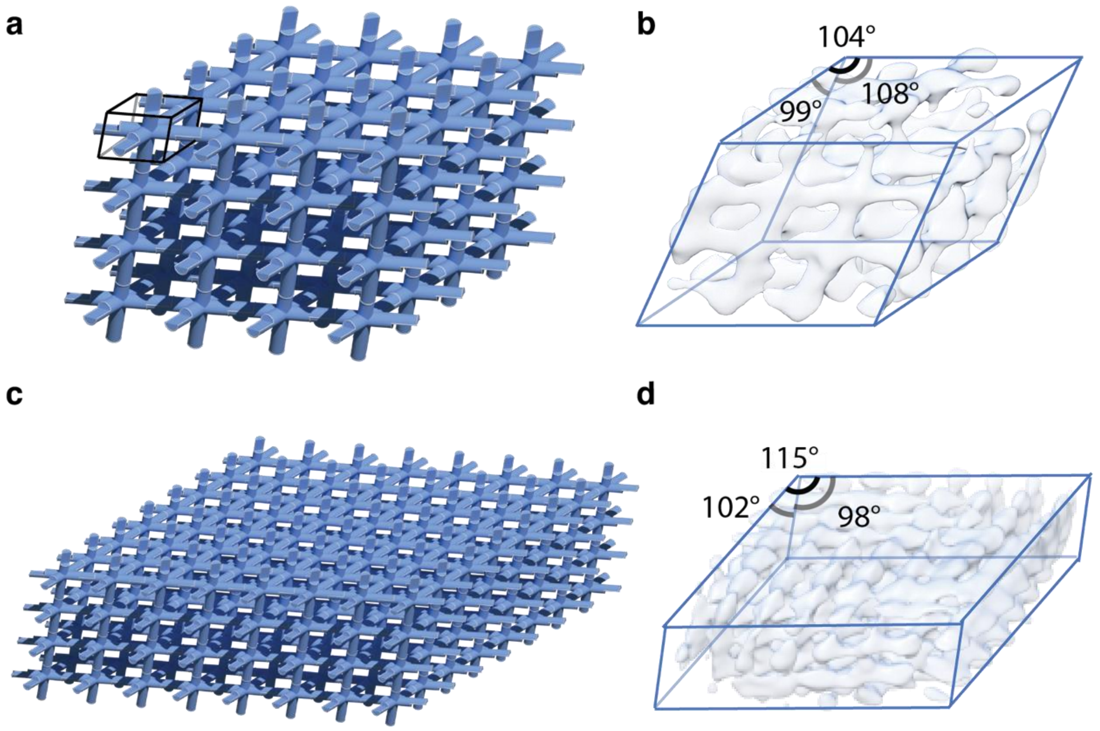

Supplementary Figure 50. Schematic diagrams of a 4×4×4 array and an 8×8×4 array. (a) Design of a 4×4×4 array. A unit cell is marked with black box. (b) 3D map of a 4×4×4 array. (c) Design of an 8×8×4 array. (d) 3D map of an 8×8×4 array. Angles between different edges of a 4×4×4 array and an 8×8×4 array were indicated in 3D maps in (b) and (d), respectively. The average angle α was provided in the main text.

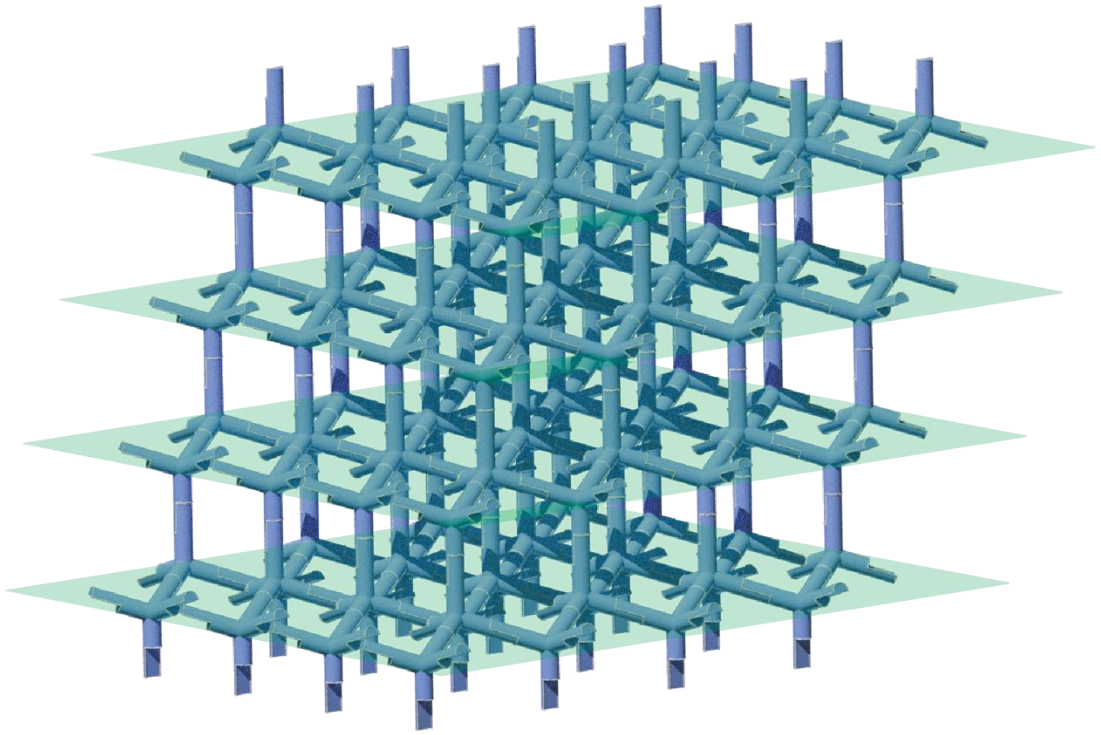

Supplementary Figure 51. Schematic diagram of a diamond cubic array. This array consists of four layers (highlighted in green) of 7×4 4-arm vertices with arms of each vertex branching out tetrahedrally. Three of the four arms of a vertex connect to neighboring vertices

of the same layer and the fourth arm branching out upward or downward to connect to a vertex of another layer.

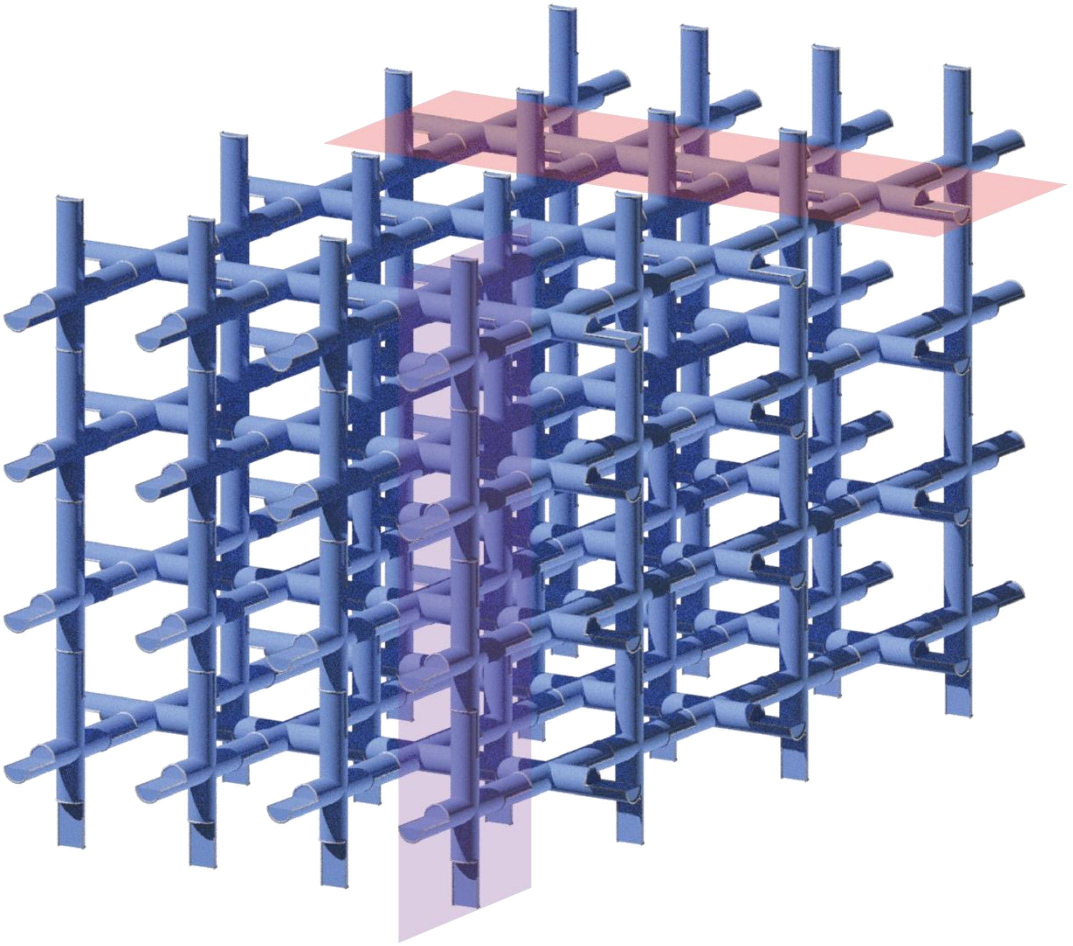

Supplementary Figure 52. Schematic diagram of a cross-like array. This array consisted of four layers of 7×4 planar 4-arm vertices of two classes of branching orientations in alternating columns (a column of horizontal branching orientation is highlighted in red and a column of vertical branching orientation is highlighted in purple.). The four arms of a vertex in the horizontal column and two arms branching out front and back of a vertex in the vertical column connect to neighboring vertices within the same layer and the other two arms branching out upward and downward of a vertex in the vertical column connect to vertices of another layers.

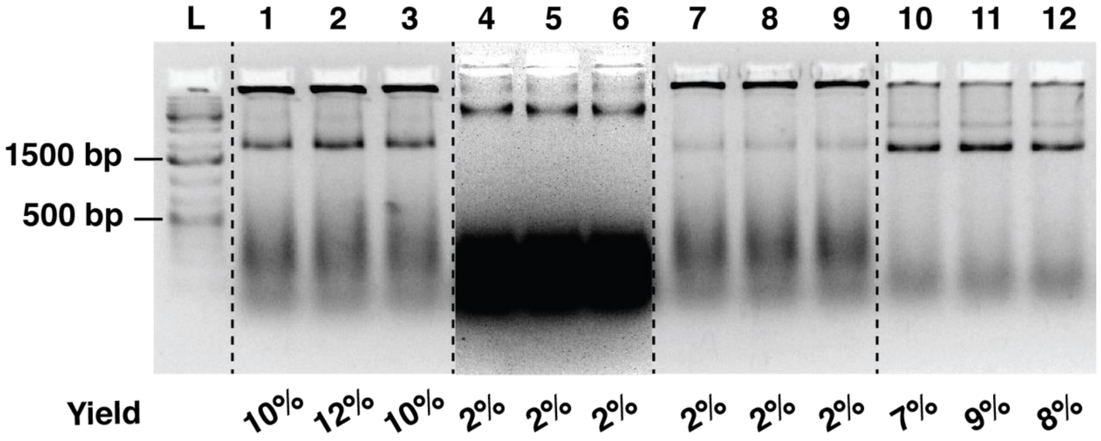

Supplementary Figure 53. Native agarose gel electrophoresis analysis for 4×4×4 array, 8×8×4 array, and diamond cubic array. Lane L: 1kb DNA ladder; lanes 1-3: three replicates of 4×4×4 array; lanes 4-6: three replicates of 8×8×4 array; lanes 7-9: three replicates of cross-like array; lanes 10-12: three replicates of diamond cubic array. Numbers at the bottom indicate assembly yields. (Results were merged from multiple gel images.)

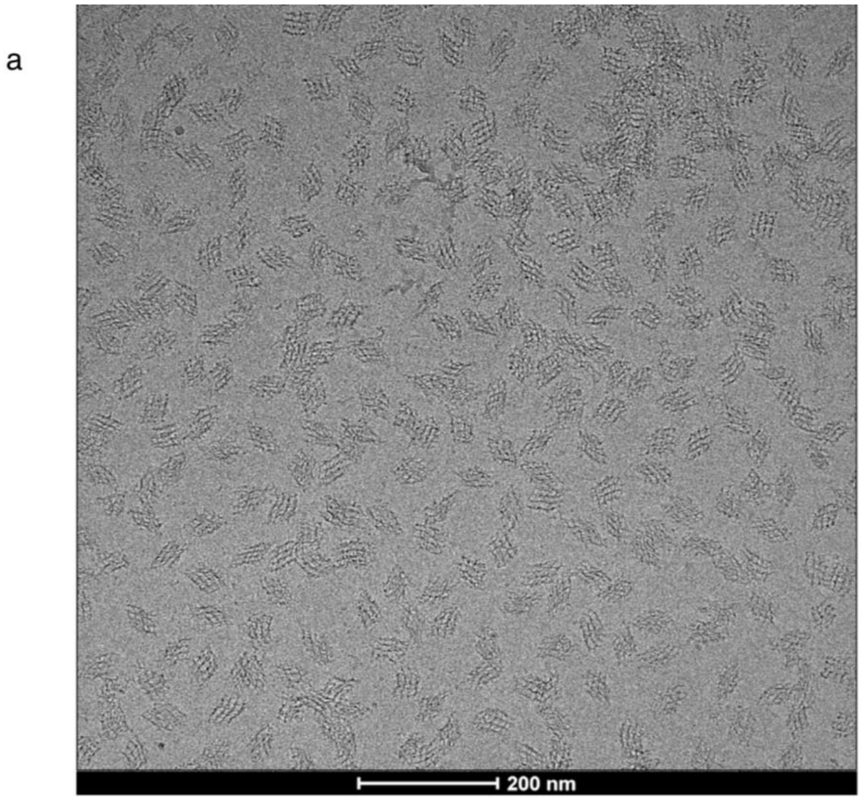

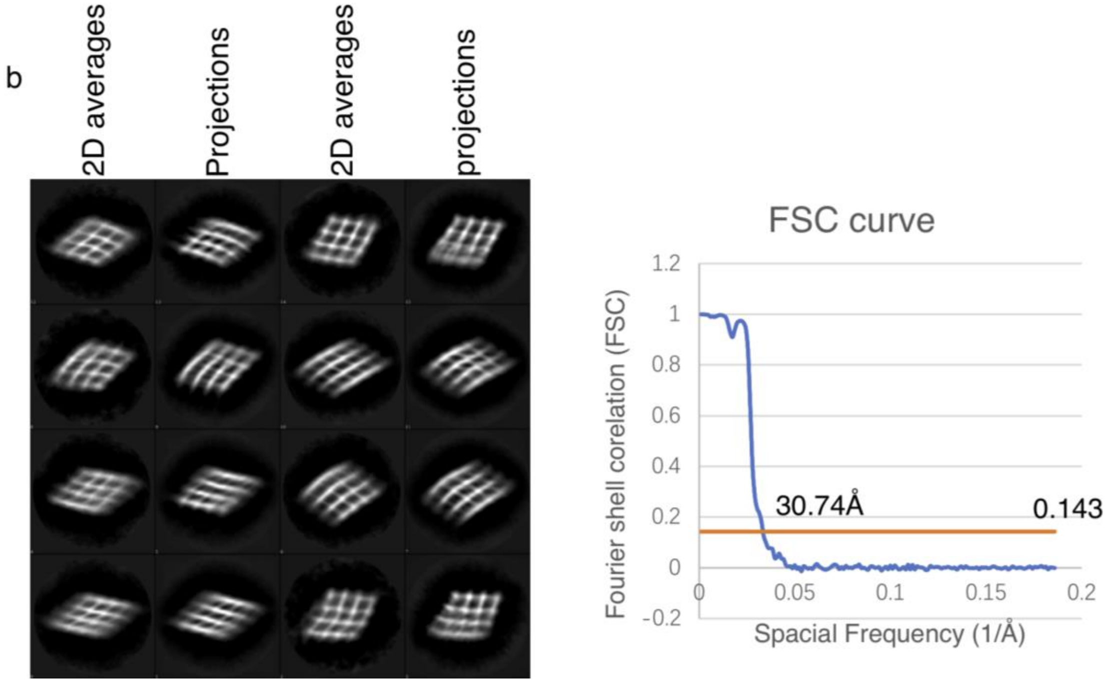

Supplementary Figure 54. Cryo-EM image and 3D reconstruction of 4×4×4 array. (a) A representative raw image. (b) Left: Representative 2D averages and corresponding projections from 3D reconstruction. Right: Gold-standard FSC plot of the 3D reconstruction.

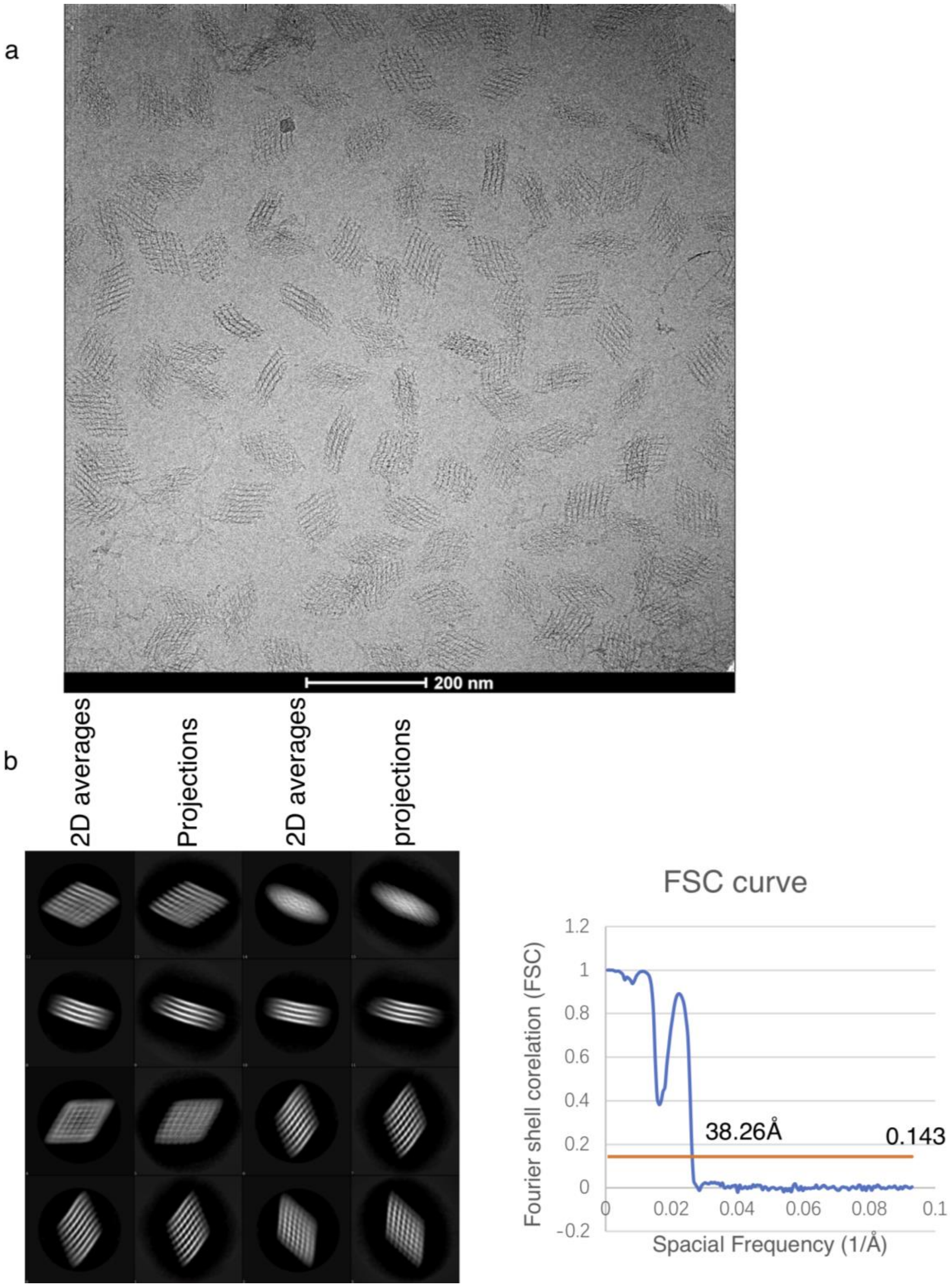

Supplementary Figure 55. Cryo-EM image and 3D reconstruction of 8×8×4 array. (a) A representative raw image. (b) Left: Representative 2D averages and corresponding projections from 3D reconstruction. Right: Gold-standard FSC plot of the 3D reconstruction.

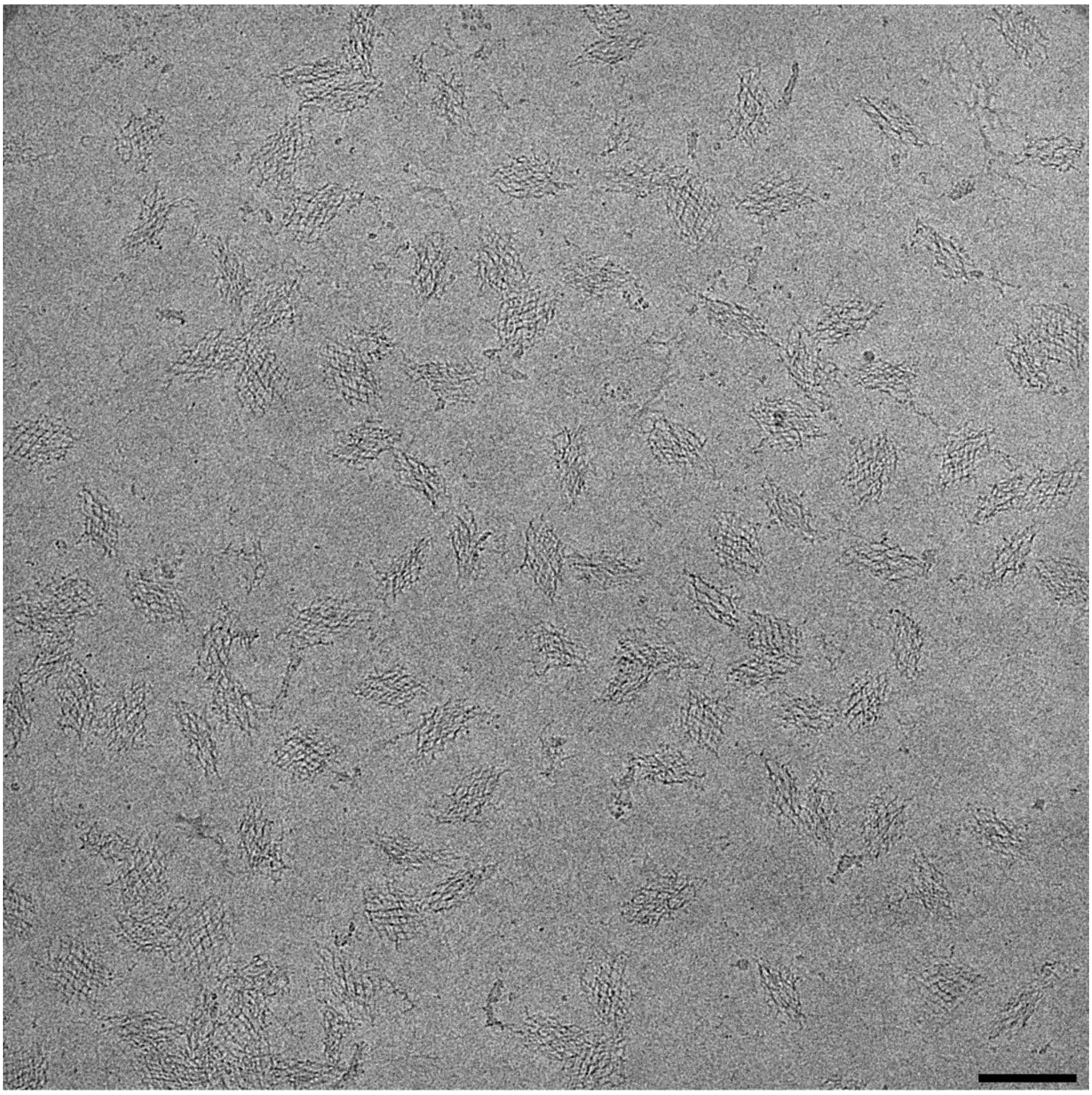

Supplementary Figure 56. Full size Cryo-EM image of diamond cubic array (scale bar: 100 nm).

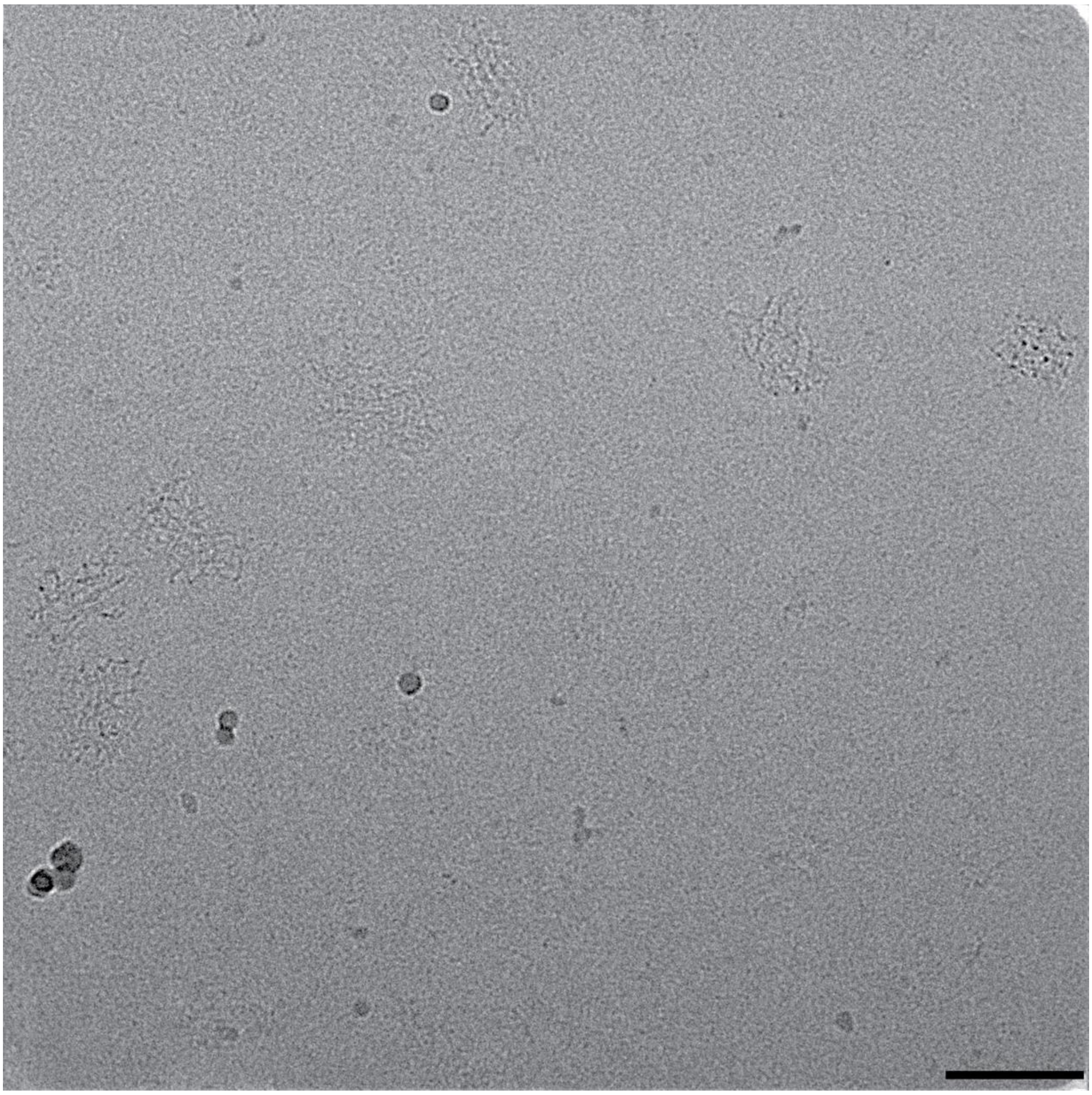

Supplementary Figure 57. Full size CryoEM image of cross-like array (scale bar: 100 nm).

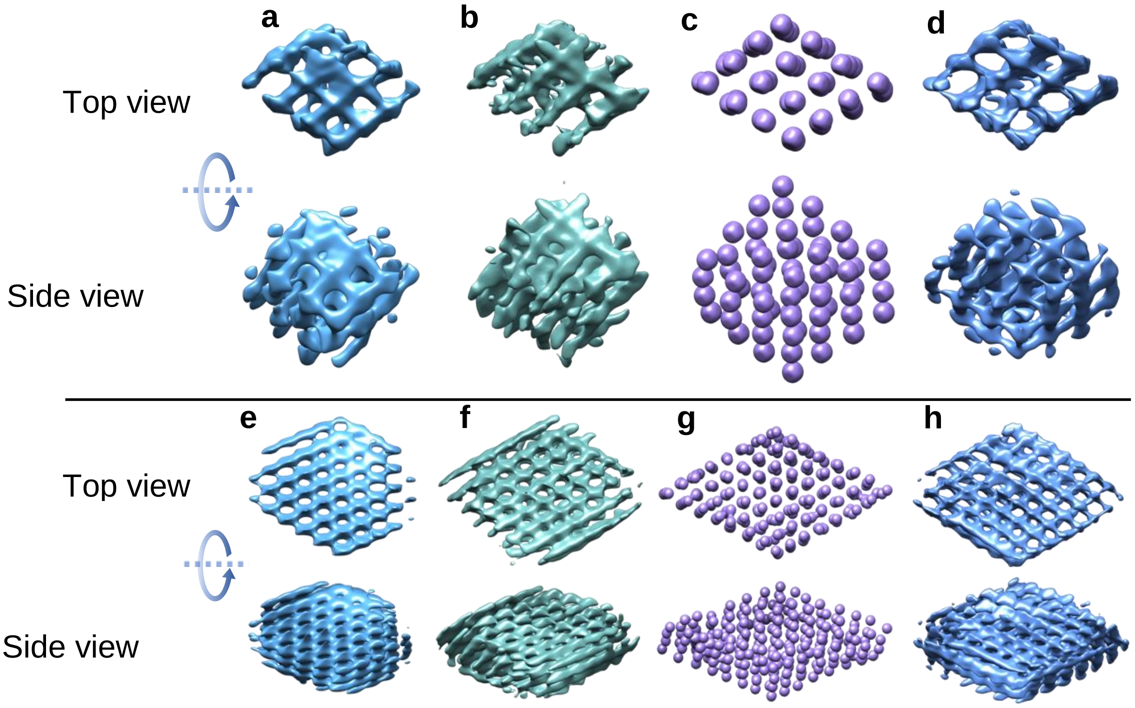

Supplementary Figure 58. Solution of the missing edges and corners problem. (a-d) Top and side views of the 4×4×4 array's 3D maps. (e-h) Top and side views of the 8×8×4 array's 3D maps. (a, e) Initial models generated by EMAN2. (b, f) Refined 3D maps with (a, e) as references. (c, g) Improved initial models. (d, f) Refined 3D maps with (c, g) as references.

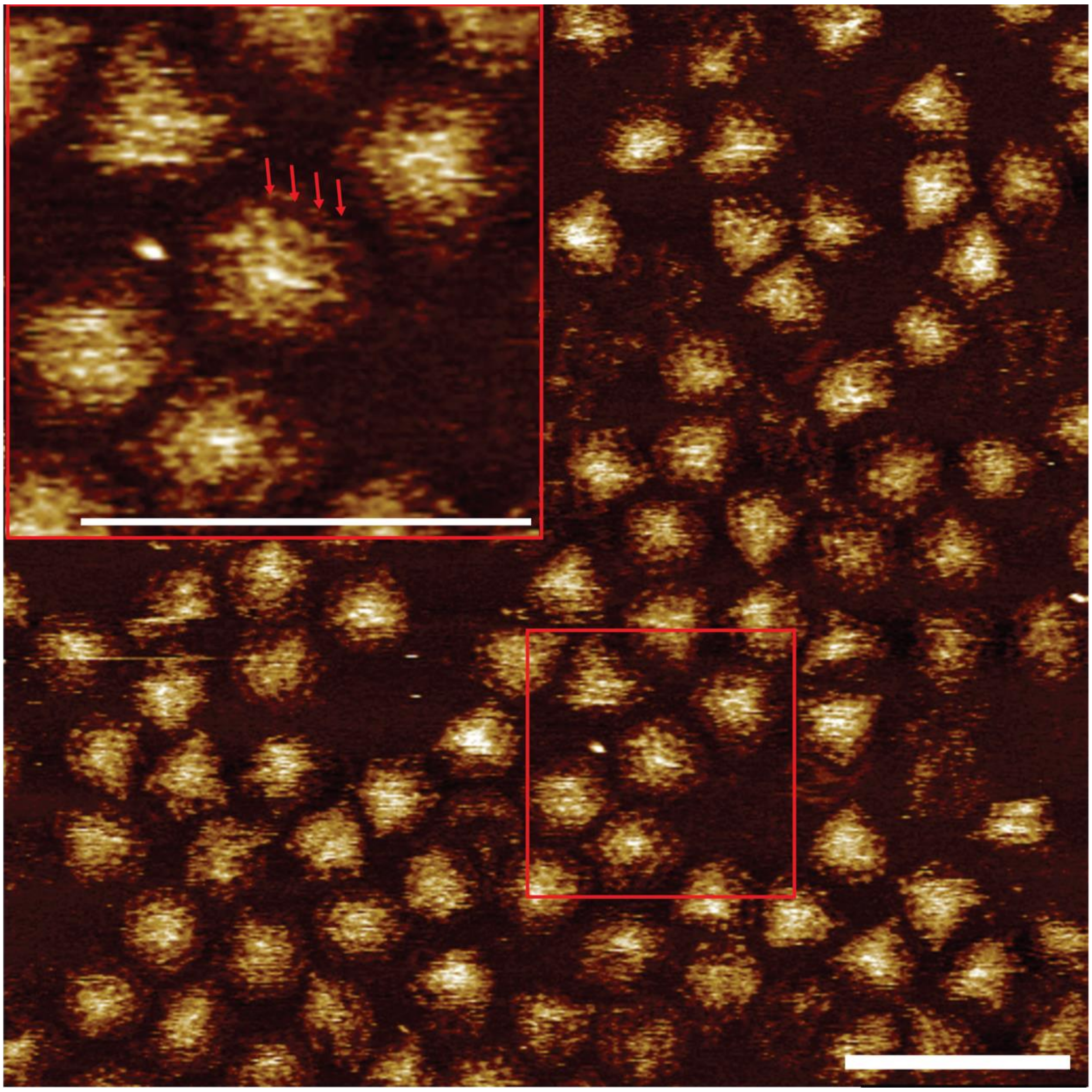

Supplementary Figure 59. Full size AFM image of 4×4×4 array. Inset shows a zoom-in image of 4×4×4 array with four layers indicated by red arrows. (Scale bars: 100 nm)

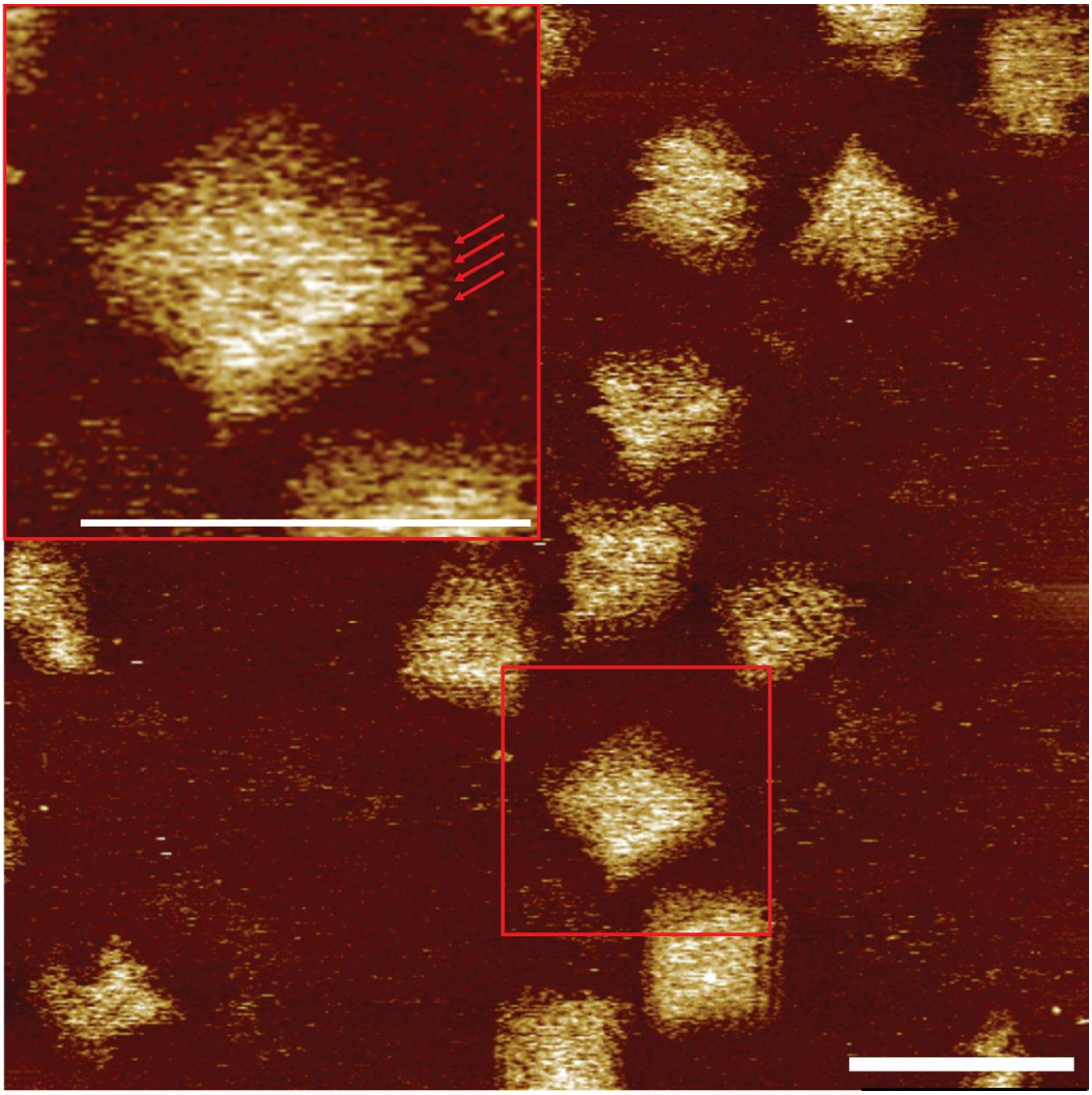

Supplementary Figure 60. Full size AFM image of 8×8×4 array. Inset shows a zoom-in image of 8×8×4 array with four layers indicated by red arrows. (Scale bars: 100 nm)

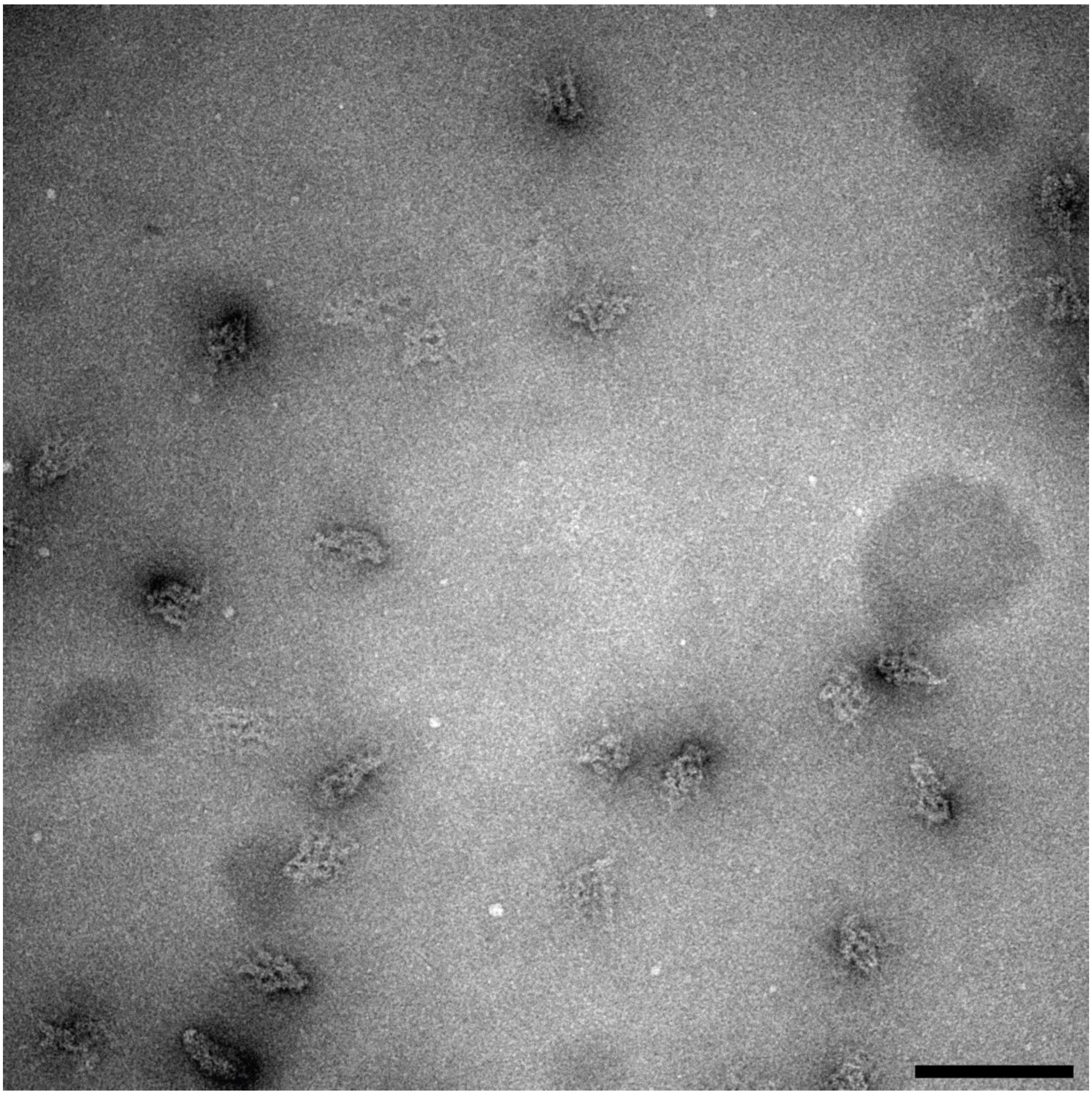

Supplementary Figure 61. Full size TEM image of 4×4×4 array.

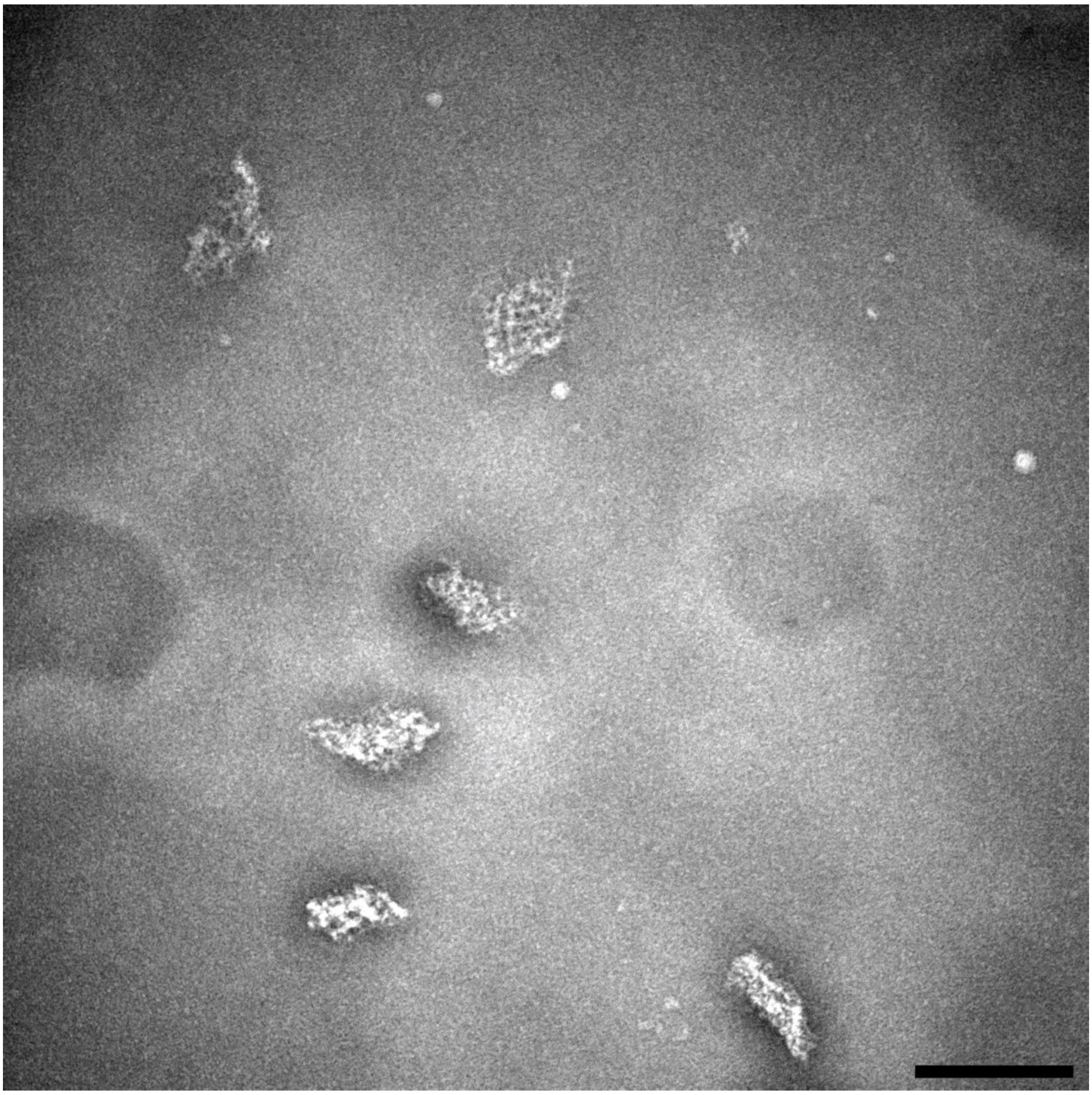

Supplementary Figure 62. Full size TEM image of 8×8×4 array.

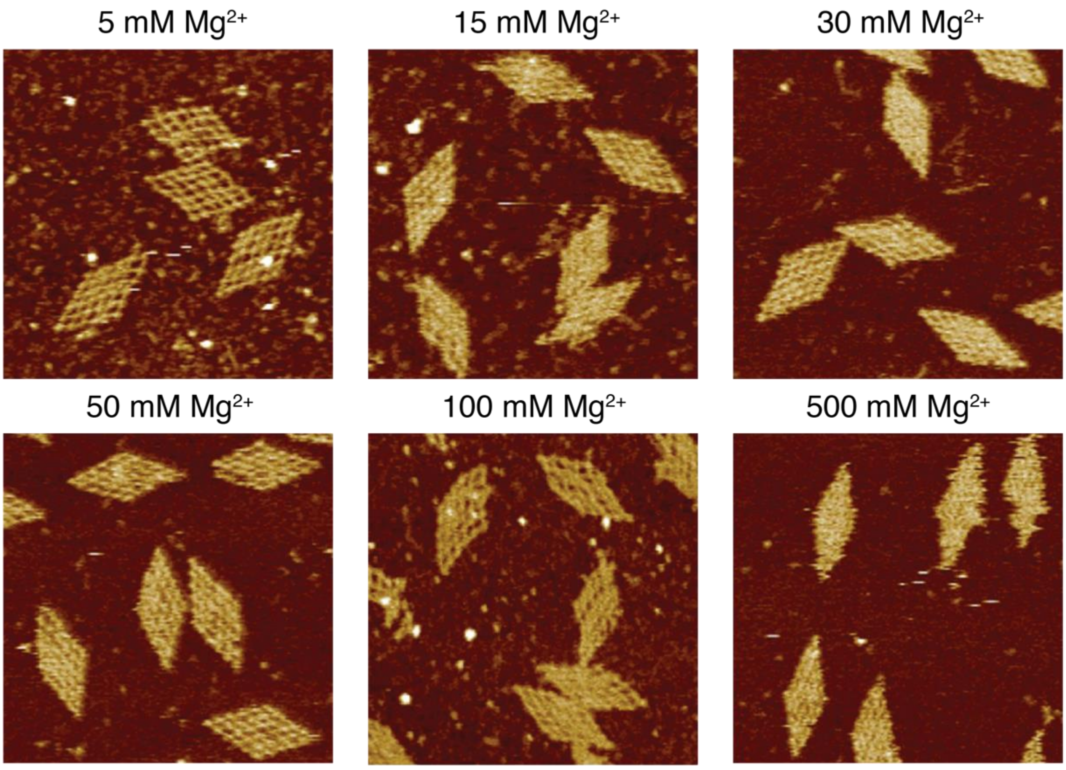

Supplementary Figure 63. AFM images of tessellation patterns from 4-arm junction vertices in different Mg^{2+} concentrations.

Supplementary Tables

Supplementary Table 1. Numbers of unpaired T base(s) added to individual structures. For structures have low assembly yields without implementation of Tn (n=1, 2 or 3) linkers, extra unpaired base(s) (e.g. 1T, 2T or 3T) were added to the crossover points at certain vertices to decrease the electrostatic repulsion caused by the neighboring arms.

Structure	Number of T base(s)
Triangular tessellation pattern with 6-arm vertices	2
Trihexagonal tessellation pattern with 4-arm vertices	2
Snub trihexagonal tessellation pattern with 5-arm vertices	2
Chimeric pattern with individual blocks composed of 3-arm, 4-arm and 6-arm vertices	2
Irregular triangular pattern 1 with 6-arm vertices	2
Irregular triangular pattern 2 with 6-arm vertices	2
Cobweb-like pattern with 4-arm vertices	2
Straight tube	3
Donut	3
U-bent tube	3
135°-bent tube	3
90°-bent tube	3
Tetrahedron	1
Octahedron	2
Cuboctahedron	2
Icosahedron	2
Triangulated cube	2

Triangulated Buckyball	1
4×4×4 array	2
8×8×4 array	2
Cross-like array	2

Supplementary Table 2. Assembly yields of 2D wireframe structures of different tessellation patterns.

Tessellation pattern type	Assembly yield (mean±SD, N=3)
Hexagonal tessellation pattern with 3-arm vertices	58%±2%
Square tessellation pattern with 4-arm vertices	70%±1%
Triangular tessellation pattern with 6-arm vertices	12%±3%
Trihexagonal tessellation pattern with 4-arm vertices	20%±1%
Snub trihexagonal tessellation pattern with 5-arm vertices	18%±1%
Chimeric pattern with individual blocks composed of 3-arm, 4-arm and 6-arm vertices	1%±0.1%
Irregular triangular pattern 1 with 6-arm vertices	18%±0.3%
Irregular triangular pattern 2 with 6-arm vertices	11%±3%
Cobweb-like pattern with 4-arm vertices	15%±2%

Supplementary Table 3. Length measurements of tessellation patterns from 4-arm vertices with alternative edge segmentation arrangements. The lengths of tessellation patterns with 4-arm vertices of different domain length combinations were measured using the 'Straight' function of ImageJ. 35 sample points were collected and measured from AFM images for each length combination. The means and standard deviations (N=35) were estimated and summarized.

Edge segmentation arrangement	Length (nm)
11-bp root domain and10-bp stem domain (edge length 32 bp)	64±4
13-bp root domain and 6-bp stem domain (edge length 32 bp)	64 ±3
16-bp root domain and10-bp stem domain (edge length 42 bp)	76±2

Supplementary Table 4. Irregular patterns with variable edge lengths.

Pattern	Edge length/bp	Root domain length/bp	Stem domain length/bp	Splinter segment
Irregular triangular pattern 1	54	18	18	N/A
Irregular triangular pattern 2	42	16	10	N/A
	52	21	10	N/A
Cobweb-like pattern	30	10	10	N/A
	40	15	10	N/A
	50	10	10	10-10-10*
	60	10	10	10-20-10*
	70	10	10	10-30-10*

*Note: A 10-10 (20/30)-10 segment means the root domain of the segment is 10 bp (20 bp/30 bp) and the stem domain of the segment is 10 bp (purple splinter segments shown in Supplementary Figure 13c).

Supplementary Table 5. Assembly yields of straight tube, donut and bent tubes.

**Tube type**	**Assembly yield (mean±SD, N=3)**
Straight tube	47%±4%
Donut	31%±2%
U-bent tube	6%±0.2%
135°-bent tube	3%±0.2%
90°-bent tube	4%±0.1%

Supplementary Table 6. Angle measurements of bent tubes. The angles of bent tubes were measured using the 'Angle tool' function of ImageJ. Different numbers of sample points were collected and measured from AFM images for each tube type. The means and standard deviations were estimated and summarized.

**Bent tube**	**Angle (°)**
U-bent tube (0 strut) (N=100)	73±33
U-bent tube (4 struts) (N=100)	139±26
U- bent tube (8 struts) (N=100)	177±6
135°-bent tube (8 struts) (N=195)	137±18
90°-bent tube (8 struts) (N=300)	104±19

Supplementary Table 7. Assembly yields of polyhedra.

**Polyhedron type**	**Assembly yield (mean±SD, N=3)**
Tetrahedron	24%±5%
Octahedron	24 %±5%
Cuboctahedron	21%±3%
Icosahedron	18%±4%
Cube	18%±2%
Triangulated cube	10%±1%
Buckyball	6%±1%
Triangulated Buckyball	1%±0.2%

Supplementary Table 8. Statistics for EM data collection and refinement of polyhedral structures.

**DNA samples**	**Microscope (FEI)**	**Magnification**	**Number of images**	**Apix (Å)**	**Total particles**	**Particles used for final refinements**	**Resolution (Å)**
Triangulated cube	Arctica	39000	223	2.69	55498	7943	28.7
Cuboctahedron	Arctica	78000	352	1.27	42044	4564	19.0
Icosahedron	Arctica	78000	184	1.27	7104	1803	15.2
Octahedron with 32-bp edges	Titan Krios	75000	434	1.16	118673	5758	14.1
Octahedron with 42-bp edges	Arctica	53000	163	1.92	101234	10428	16.5
Octahedron with 52-bp edges	Arctica	53000	54	1.92	21557	1968	23.5
Tetrahedron	Arctica	78000	88	1.27	46626	1838	20.3
Triangulated Buckyball	Arctica	39000	410	2.69	18311	1740	37.2
Octahedron-protein	Arctica	53000	120	1.92	71455	236	26.6

Supplementary Table 9. Assembly yields of 4×4×4 array, 8×8×4 array, diamond cubic array and cross-like array.

Array type	Assembly yield (mean±SD, N=3)
4×4×4 array	11%±1%
8×8×4 array	2%±0.1%
diamond cubic array	8%±1%
cross-like array	2%±0.2%

Supplementary Table 10. Statistics for EM data collection and refinement of 4×4×4 and 8×8×4 arrays.

DNA samples	Microscope (FEI)	Magnification	Number of images	Apix (Å)	Total particles	Particles used for final refinements	Resolution (Å)
4×4×4 array	Arctica	39000	569	2.69	61731	45976	30.7
8×8×4 array	Arctica	39000	1968	2.69	56278	47493	38.2

Supplementary Table 11. Packing efficiencies of different structures. Most of the resulted wireframe structures are porous, so the packing efficiency are much lower than the counterparts with compact helices.

Structure	Packing efficiency
Hexagonal tessellation pattern with 3-arm vertices	12%
Square tessellation pattern with 4-arm vertices (32-bp edge)	24%
Square tessellation pattern with 4-arm vertices (42-bp edge)	18%
Triangular tessellation pattern with 6-arm vertices	36%
4×4×4 array	11%
Square packed 2D origami	52%
Square packed 3D origami	50%
Honeycomb packed 3D origami	39%

Supplementary Table 12. Angle measurements of tessellation patterns with 4-arm vertices in different Mg^{2+} concentrations. The angles of tessellation patterns from 4-arm junction vertices were measured using the 'Angle tool' function of ImageJ. 30-90 sample points were collected and measured from AFM images. The means and standard deviations were estimated and summarized.

Mg^{2+} (mM)	Angle (°)	Number of samples
5	50±3	57
15	48±3	54
30	46±2	67
50	44±3	90
100	43±4	30
500	37±2	31

Supplementary Notes

Supplementary Note 1. Density subtraction

The extra density was isolated by density subtraction using the program relion_image_handler of the RELION2 package. The position and size of the positive part of the extra density (Figure 4h, bottom right, green) matched with our design of the binding protein well. However, the negative part of the extra density (Figure 4h, bottom right, blue) which belongs to the free DNA octahedron structure indicated that the DNA scaffold in the complex shrank a little compared with the free octahedron. And this distortion might be caused by the binding of the protein. 3D

maps of the complex showed the attachment of the MBP proteins at designed sites and in desired orientations (Supplementary Figure 49).

Supplementary Note 2. Improvement of the 4×4×4 and 8×8×4 array models

The initial model of the 4×4×4 or 8×8×4 array generated with e2initialmodel.py in EMAN2 package was acceptable but there were missing densities at edges and corners of the rhomboid-shaped structure (Supplementary Figure 58, a and e). This led to improper alignment of 2D particle images against the initial models, which in turn resulted in missing edges or corners after 3D refinement (Supplementary Figure 58, b and f). To improve the initial model, we used Coot[1] to generate a new coordinate file by placing atoms at the desired vertices according to the design. Then the improved initial model (Supplementary Figure 58, c and g) was generated from the coordinate file using e2pdb2mrc.py in the EMAN2 package. Another round of 3D refinement was performed (Supplementary Figure 58, d and h) with the improved model as a reference.

Supplementary Note 3. Influence of cation (Mg^{2+}) concentration on structural morphology

On the flip side, the structural flexibility indicates that the resulted structures are dynamic and structural reconfiguration can be designed. For example, higher magnesium ion concentration can alleviate the repulsion of DNA helices and hence reduce the angle of the tessellation patterns from 4-arm vertices.

The influence of Mg^{2+} concentration on the morphology of tessellation patterns from 4-arm junction vertices was visualized by AFM. For each tested $MgCl_2$ concentration (from 5 mM to 500 mM), 5 μL purified sample (sample annealed in 0.5×TE buffer supplemented with 15 mM $MgCl_2$) was mixed with 40 μL 0.5×TE buffer supplemented with the tested $MgCl_2$ concentration and left for 30 mins. Then the mixture was applied to a freshly cleaved mica surface and left for approximately 2 mins. Supplementary 10 μL 10 mM $NiCl_2$ was added to increase the strength of DNA–mica binding. Samples were imaged under liquid ScanAsyst mode, with C-type triangular tips from the SNL-10 silicon nitride cantilever chip. AFM images of tessellation patterns from 4-arm junction vertices in different Mg^{2+} concentrations are shown in Supplementary Figure 63.

Supplementary Reference

1 Emsley, P., Lohkamp, B., Scott, W. G. & Cowtan, K. Features and development of Coot. *Acta Crystallographica* **66**, 486-501(2010).
