# Supplementary material for: Complex wireframe DNA nanostructures from simple building blocks
Source: Nat Commun. 2019 Mar 6;10:1067. doi: 10.1038/s41467-019-08647-7 (PMC6403373; doi:10.1038/s41467-019-08647-7)
Supplement: Supplementary file 13 — Description of Additional Supplementary Files [file 41467_2019_8647_MOESM13_ESM.docx]

**Title:** Supplementary Data
**Description:** DNA Sequences

**Title:** Supplementary Movie 1
**Description:** Tetrahedron with 3-arm vertices.

**Title:** Supplementary Movie 2
**Description:** Octahedron with 32-bp edges.

**Title:** Supplementary Movie 3
**Description:** Octahedron with 42-bp edges.

**Title:** Supplementary Movie 4
**Description:** Octahedron with 52-bp edges.

**Title:** Supplementary Movie 5
**Description:** Cuboctahedron with 4-arm vertices.

**Title:** Supplementary Movie 6
**Description:** Icosahedron with 5-arm vertices.

**Title:** Supplementary Movie 7
**Description:** Triangulated cube with 6-arm vertices.

**Title:** Supplementary Movie 8
**Description:** Triangulated Bucky ball with 5-arm and 6-arm vertices.

**Title:** Supplementary Movie 9
**Description:** Protein display on an octahedron.

**Title:** Supplementary Movie 10
**Description:** 4×4×4 array. Supplementary Movie 11: 8×8×4 array
